# Global, regional, and national prevalence of adult overweight and obesity, 1990–2021, with forecasts to 2050: a forecasting study for the Global Burden of Disease Study 2021

**DOI:** 10.1016/S0140-6736(25)00355-1

**Published:** 2025-03-08

**Authors:** GBD 2021 Adult BMI Collaborators, Marie Ng, Emmanuela Gakidou, Justin Lo, Yohannes Habtegiorgis Abate, Cristiana Abbafati, Nasir Abbas, Mohammadreza Abbasian, Samar Abd ElHafeez, Wael M Abdel-Rahman, Sherief Abd-Elsalam, Arash Abdollahi, Meriem Abdoun, Deldar Morad Abdulah, Rizwan Suliankatchi Abdulkader, Auwal Abdullahi, Armita Abedi, Hansani Madushika Abeywickrama, Alemwork Abie, Richard Gyan Aboagye, Shady Abohashem, Dariush Abtahi, Hasan Abualruz, Bilyaminu Abubakar, Rana Kamal Abu Farha, Hana J Abukhadijah, Niveen ME Abu-Rmeileh, Salahdein Aburuz, Ahmed Abu-Zaid, Lisa C Adams, Mesafint Molla Adane, Isaac Yeboah Addo, Kamoru Ademola Adedokun, Nurudeen A Adegoke, Abiola Victor Victor Adepoju, Ridwan Olamilekan Adesola, Temitayo Esther Adeyeoluwa, Usha Adiga, Qorinah Estiningtyas Sakilah Adnani, Siamak Afaghi, Saira Afzal, Muhammad Sohail Afzal, Thilini Chanchala Agampodi, Shahin Aghamiri, César Agostinis Sobrinho, Williams Agyemang-Duah, Austin J Ahlstrom, Danish Ahmad, Sajjad Ahmad, Aqeel Ahmad, Muayyad M Ahmad, Fuzail Ahmad, Noah Ahmad, Haroon Ahmed, Muktar Beshir Ahmed, Ayman Ahmed, Meqdad Saleh Ahmed, Mehrunnisha Sharif Ahmed, Syed Anees Ahmed, Marjan Ajami, Samina Akhtar, Mohammed Ahmed Akkaif, Ashley E Akrami, Tariq A Alalwan, Ziyad Al-Aly, Khurshid Alam, Rasmieh Mustafa Al-amer, Amani Alansari, Fahmi Y Al-Ashwal, Mohammed Albashtawy, Wafa A Aldhaleei, Bezawit Abeje Alemayehu, Abdelazeem M Algammal, Khalid F Alhabib, Hanadi Al Hamad, Syed Mahfuz Al Hasan, Dari Alhuwail, Rafat Ali, Abid Ali, Waad Ali, Mohammed Usman Ali, Sheikh Mohammad Alif, Samah W Al-Jabi, Syed Mohamed Aljunid, Ahmad Alkhatib, Sabah Al-Marwani, Mahmoud A Alomari, Saleh A Alqahtani, Rajaa M Mohammad Al-Raddadi, Ahmad Alrawashdeh, Intima Alrimawi, Sahel Majed Alrousan, Najim Z Alshahrani, Omar Al Ta'ani, Zain Al Ta'ani, Zaid Altaany, Awais Altaf, Yazan Al Thaher, Nelson Alvis-Guzman, Mohammad Al-Wardat, Yaser Mohammed Al-Worafi, Safwat Aly, Hany Aly, Hosam Alzahrani, Abdallah Alzoubi, Karem H Alzoubi, Md. Akib Al-Zubayer, Sohrab Amiri, Hubert Amu, Dickson A Amugsi, Ganiyu Adeniyi Amusa, Roshan A Ananda, Robert Ancuceanu, Catalina Liliana Andrei, Ranjit Mohan Anjana, Sumbul Ansari, Mohammed Tahir Ansari, Catherine M Antony, Iyadunni Adesola Anuoluwa, Boluwatife Stephen Anuoluwa, Saeid Anvari, Saleha Anwar, Anayochukwu Edward Anyasodor, Geminn Louis Carace Apostol, Juan Pablo Arab, Jalal Arabloo, Mosab Arafat, Aleksandr Y Aravkin, Demelash Areda, Hidayat Arifin, Mesay Arkew, Benedetta Armocida, Johan Ärnlöv, Mahwish Arooj, Anton A Artamonov, Kurnia Dwi Artanti, Ashokan Arumugam, Mohammad Asghari-Jafarabadi, Tahira Ashraf, Bernard Kwadwo Yeboah Asiamah-Asare, Anemaw A Asrat, Thomas Astell-Burt, Seyyed Shamsadin Athari, Prince Atorkey, Alok Atreya, Zaure Maratovna Aumoldaeva, Hamzeh Awad, Mamaru Ayenew Awoke, Adedapo Wasiu Awotidebe, Setognal Birara Aychiluhm, Ali Azargoonjahromi, Amirali Azimi, Sadat Abdulla Aziz, Shahkaar Aziz, Ahmed Y. Azzam, Domenico Azzolino, Peter S Azzopardi, Mina Babashahi, Giridhara Rathnaiah Babu, Ashish D Badiye, Nasser Bagheri, Yogesh Bahurupi, Ruhai Bai, Atif Amin Baig, Shankar M Bakkannavar, Senthilkumar Balakrishnan, Ovidiu Constantin Baltatu, Kiran Bam, Maciej Banach, Rajon Banik, Mainak Bardhan, Hiba Jawdat Barqawi, Simon Barquera, Lingkan Barua, Zarrin Basharat, Shahid Bashir, Mohammad-Mahdi Bastan, Saurav Basu, Reza Bayat, Mulat Tirfie Bayih, Narasimha M Beeraka, Tahmina Begum, Umar Muhammad Bello, Abdulrahman Babatunde Bello, Luis Belo, Isabela M Bensenor, Maria Bergami, Kidanemaryam Berhe, Abiye Assefa Berihun, Ajeet Singh Bhadoria, Akshaya Srikanth Bhagavathula, Neeraj Bhala, Jaideep Singh Bhalla, Ravi Bharadwaj, Pankaj Bhardwaj, Nikha Bhardwaj, Sonu Bhaskar, Ajay Nagesh Bhat, Priyadarshini Bhattacharjee, Shuvarthi Bhattacharjee, Jasvinder Singh Bhatti, Gurjit Kaur Bhatti, Andras Bikov, Cem Bilgin, Catherine Bisignano, Bijit Biswas, Bruno Bizzozero Peroni, Espen Bjertness, Tone Bjørge, Srinivasa Rao Bolla, Hamed Borhany, Samuel Adolf Bosoka, Souad Bouaoud, Edward J Boyko, Dejana Braithwaite, Javier Brazo-Sayavera, Hermann Brenner, Gabrielle Britton, Dana Bryazka, Raffaele Bugiardini, Linh Phuong Bui, Felix Busch, Yasser Bustanji, Nadeem Shafique Butt, Zahid A Butt, Daniela Calina, Luciana Aparecida Campos, Ismael Campos-Nonato, Si Cao, Yin Cao, Angelo Capodici, Andre F Carvalho, Márcia Carvalho, Alberico L Catapano, Monica Cattafesta, Maria Sofia Cattaruzza, Luca Cegolon, Francieli Cembranel, Edina Cenko, Ester Cerin, Achille Cernigliaro, Joshua Chadwick, Chiranjib Chakraborty, Raymond N C Chan, Jung-Chen Chang, Vijay Kumar Chattu, Anis Ahmad Chaudhary, Akhilanand Chaurasia, Guangjin Chen, An-Tian Chen, Haowei Chen, Esther T W Cheng, Nicholas WS Chew, Gerald Chi, Ritesh Chimoriya, Patrick R Ching, Dong-Woo Choi, Bryan Chong, Hitesh Chopra, Shivani Chopra, Hou In Chou, Sonali Gajanan Choudhari, Dinh-Toi Chu, Sunghyun Chung, Sheng-Chia Chung, Muhammad Chutiyami, Karly I Cini, Iolanda Cioffi, Rebecca M Cogen, Daniel Collado-Mateo, Alyssa Columbus, Nathalie Conrad, Michael H Criqui, Natalia Cruz-Martins, Steven Cummins, Emanuele D'Amico, Lucio D'Anna, Mario D'Oria, Omid Dadras, Xiaochen Dai, Mayank Dalakoti, Rakhi Dandona, Lalit Dandona, Pojsakorn Danpanichkul, Samuel Demissie Darcho, Reza Darvishi Cheshmeh Soltani, Alanna Gomes da Silva, Kairat Davletov, Ivan Delgado-Enciso, Edgar Denova-Gutiérrez, Meseret Derbew Molla, Ismail Dergaa, Aragaw Tesfaw Desale, Vinoth Gnana Chellaiyan Devanbu, Devananda Devegowda, Syed Masudur Rahman Dewan, Arkadeep Dhali, Samath Dhamminda Dharmaratne, Meghnath Dhimal, Bibha Dhungel, Daniel Diaz, Monica Dinu, Milad Dodangeh, Sushil Dohare, Klara Georgieva Dokova, Neda Dolatkhah, Camila Bruneli do Prado, Fariba Dorostkar, Ojas Prakashbhai Doshi, Rajkumar Prakashbhai Doshi, Robert Kokou Dowou, Viola Savy Dsouza, Mi Du, Samuel C Dumith, Dorothea Dumuid, Bruce B Duncan, Sulagna Dutta, Arkadiusz Marian Dziedzic, Alireza Ebrahimi, Behrad Eftekhari, Ashkan Eighaei Sedeh, Michael Ekholuenetale, Mohamed Ahmed Eladl, Rabie Adel El Arab, Said El-Ashker, Iffat Elbarazi, Ibrahim Farahat El Bayoumy, Islam Y Elgendy, Muhammed Elhadi, Waseem El-Huneidi, Ashraf A El-Metwally, Mohamed A Elmonem, Mohamed Hassan Elnaem, Randa Elsheikh, Ibrahim Elsohaby, Chadi Eltaha, Theophilus I Emeto, Maysa Eslami, Ugochukwu Anthony Eze, Heidar Fadavian, Adeniyi Francis Fagbamigbe, Ildar Ravisovich Fakhradiyev, Seyed Nooreddin Faraji, Carla Sofia e Sá Farinha, MoezAlIslam Ezzat Mahmoud Faris, Umar Farooque, Hossein Farrokhpour, Samuel Aanuoluwapo Fasusi, Patrick Fazeli, Timur Fazylov, Alireza Feizkhah, Ginenus Fekadu, Xiaoqi Feng, João C Fernandes, Rodrigo Fernandez-Jimenez, Nuno Ferreira, Bikila Regassa Feyisa, Florian Fischer, David Flood, Nataliya A Foigt, Morenike Oluwatoyin Folayan, Artem Alekseevich Fomenkov, Roham Foroumadi, Celia Fortuna Rodrigues, Matteo Foschi, Maryam Fotouhi, Kate Louise Francis, Richard Charles Franklin, Aleš Gába, Muktar A Gadanya, Abhay Motiramji Gaidhane, Yaseen Galali, Silvano Gallus, Balasankar Ganesan, Shivaprakash Gangachannaiah, Wendy Paola Gastélum Espinoza, Miglas Welay Gebregergis, Teferi Gebru Gebremeskel, Lemma Getacher, Fataneh Ghadirian, Amir Ghaffari Jolfayi, Seyyed-Hadi Ghamari, Ramy Mohamed Ghazy, Artyom Urievich Gil, Tiffany K Gill, Elena V Gnedovskaya, Mahaveer Golechha, Davide Golinelli, Michal Grivna, Ashna Grover, Zhongyang Guan, Shi-Yang Guan, Giovanni Guarducci, Mohammed Ibrahim Mohialdeen Gubari, Avirup Guha, Damitha Asanga Gunawardane, Zheng Guo, Rajeev Gupta, Anish Kumar Gupta, Rahul Gupta, Sapna Gupta, Vivek Kumar Gupta, Roberth Steven Gutiérrez-Murillo, Jose Guzman-Esquivel, Najah R Hadi, Zahra Hadian, Nadia M Hamdy, Sajid Hameed, Samer Hamidi, Mohammad Hamiduzzaman, Asif Hanif, Nasrin Hanifi, Graeme J Hankey, Allie Haq, Netanja I Harlianto, Josep Maria Haro, Risky Kusuma Hartono, Faizul Hasan, Mohammad Hashem Hashempur, Md Saquib Hasnain, Amr Hassan, Nageeb Hassan, Soheil Hassanipour, Afagh Hassanzade Rad, Rasmus J Havmoeller, Simon I Hay, Wen-Qiang He, Jeffrey J Hebert, Golnaz Heidari, Mehdi Hemmati, Yuta Hiraike, Nguyen Quoc Hoan, Mai Hoang, Ramesh Holla, Praveen Hoogar, Ashley Mark Hopkins, Alamgir Hossain, Hassan Hosseinzadeh, Sorin Hostiuc, Mihaela Hostiuc, Zin Wai Htay, Chengxi Hu, Junjie Huang, Tsegaye Gebreyes Hundie, Mohamed Ibrahim Husseiny, Hong-Han Huynh, Ivo Iavicoli, Anel Ibrayeva, Olayinka Stephen Ilesanmi, Irena M Ilic, Milena D Ilic, Mohammad Tarique Imam, Leeberk Raja Inbaraj, Arit Inok, Lalu Muhammad Irham, Md. Rabiul Islam, Sheikh Mohammed Shariful Islam, Rakibul M Islam, Nahlah Elkudssiah Ismail, Hiroyasu Iso, Gaetano Isola, Mosimah Charles Ituka, Masao Iwagami, Chinwe Juliana Iwu-Jaja, Assefa N Iyasu, Vinothini J, Louis Jacob, Shabbar Jaffar, Haitham Jahrami, Akhil Jain, Ammar Abdulrahman Jairoun, Mihajlo Jakovljevic, Mohamed Lamrana Jalloh, Syed Sarmad Javaid, Sathish Kumar Jayapal, Umesh Jayarajah, Shubha Jayaram, Rime Jebai, Felix K Jebasingh, Alelign Tasew Jema, Mohammad Jokar, Jost B Jonas, Jobinse Jose, Nitin Joseph, Charity Ehimwenma Joshua, Jacek Jerzy Jozwiak, Mikk Jürisson, Billingsley Kaambwa, Ali Kabir, Zubair Kabir, Ashish Kumar Kakkar, Sanjay Kalra, Sivesh Kathir Kamarajah, Saddam Fuad Kanaan, Samuel Berchi Kankam, Kehinde Kazeem Kanmodi, Neeti Kapoor, Mehrdad Karajizadeh, Paschalis Karakasis, Reema A Karasneh, Yeganeh Karimi, Arman Karimi Behnagh, Nicholas J Kassebaum, Joonas H Kauppila, Gbenga A Kayode, Dimitrios Kehagias, Jessica A Kerr, Ariz Keshwani, Emmanuelle Kesse-Guyot, Mohammad Keykhaei, Inn Kynn Khaing, Himanshu Khajuria, Pantea Khalili, Alireza Khalilian, Mohamed Khalis, Mohammad Jobair Khan, Maseer Khan, Nusrat Khan, Md Abdullah Saeed Khan, Ajmal Khan, Moien AB Khan, Shaghayegh Khanmohammadi, Khaled Khatab, Moawiah Mohammad Khatatbeh, Maryam Khayamzadeh, Feriha Fatima Khidri, Fatemeh Khorashadizadeh, Atulya Aman Khosla, Sepehr Khosravi, Mahmood Khosrowjerdi, Jagdish Khubchandani, Helda Khusun, Jinho Kim, Kwanghyun Kim, Min Seo Kim, Yun Jin Kim, Ruth W Kimokoti, Adnan Kisa, Ladli Kishore, Mika Kivimäki, Michail Kokkorakis, Ali-Asghar Kolahi, Farzad Kompani, Oleksii Korzh, Karel Kostev, Sindhura Lakshmi Koulmane Laxminarayana, Irene Akwo Kretchy, Kewal Krishan, Chong-Han Kua, Barthelemy Kuate Defo, Mukhtar Kulimbet, Vishnutheertha Kulkarni, Ashish Kumar, Vijay Kumar, G Anil Kumar, Satyajit Kundu, Setor K Kunutsor, Om P Kurmi, Maria Dyah Kurniasari, Dian Kusuma, Ville Kytö, Ben Lacey, Chandrakant Lahariya, Daphne Teck Ching Lai, Hanpeng Lai, Iván Landires, Bagher Larijani, Kamaluddin Latief, Carlo La Vecchia, Nhi Huu Hanh Le, Munjae Lee, Sang-woong Lee, Wei-Chen Lee, Seung Won Lee, Paul H Lee, Ming-Chieh Li, Yongze Li, Weilong Li, Stephen S Lim, Queran Lin, Jialing Lin, Daniel Lindholm, Paulina A Lindstedt, Simin Liu, Erand Llanaj, José Francisco López-Gil, Stefan Lorkowski, Giancarlo Lucchetti, Alessandra Lugo, Angelina M Lutambi, Lei Lv, Ellina Lytvyak, Zheng Feei Ma, Monika Machoy, Javier A Magaña Gómez, Nastaran Maghbouli, Mehrdad Mahalleh, Nozad H Mahmood, Elham Mahmoudi, Rituparna Maiti, Konstantinos Christos C Makris, Kashish Malhotra, Ahmad Azam Malik, Iram Malik, Deborah Carvalho Malta, Abdullah A Mamun, Marjan Mansourian, Emmanuel Manu, Hamid Reza Marateb, Mirko Marino, Abdoljalal Marjani, Ramon Martinez-Piedra, Santi Martini, Miquel Martorell, Sammer Marzouk, Stefano Masi, Soroush Masrouri, Yasith Mathangasinghe, Manu Raj Mathur, Fernanda Penido Matozinhos, Thushara Matthias, Rita Mattiello, Mohsen Mazidi, Steven M McPhail, Enkeleint A Mechili, Riffat Mehboob, Asim Mehmood, Man Mohan Mehndiratta, Kamran Mehrabani-Zeinabad, Tesfahun Mekene Meto, Hadush Negash Meles, Walter Mendoza, Ritesh G Menezes, Emiru Ayalew Mengistie, Sultan Ayoub Meo, Tomislav Mestrovic, Sachith Mettananda, Chamila Dinushi Kukulege Mettananda, Ana Carolina Micheletti Gomide Nogueira de Sá, Ted R Miller, GK Mini, Erkin M Mirrakhimov, Awoke Misganaw, Madhukar Mittal, Ahmed Ismail Mohamed, Mona Gamal Mohamed, Nouh Saad Mohamed, Jama Mohamed, Taj Mohammad, Sakineh Mohammad-Alizadeh-Charandabi, Ibrahim Mohammadzadeh, Shafiu Mohammed, Mustapha Mohammed, Ali H Mokdad, Stefania Mondello, Mohammad Ali Moni, Maryam Moradi, Shane Douglas Morrison, Elias Mossialos, Rohith Motappa, Francesk Mulita, Erin C Mullany, Yanjinlkham Munkhsaikhan, Efren Murillo-Zamora, Sani Musa, Ghulam Mustafa, Sathish Muthu, Woojae Myung, Pirouz Naghavi, Mohsen Naghavi, Ganesh R Naik, Hiten Naik, Gopal Nambi, Vinay Nangia, Jobert Richie Nansseu, Gustavo G Nascimento, Mahmoud Nassar, Zuhair S Natto, Javaid Nauman, Zakira Naureen, Samidi Nirasha Kumari Navaratna, Biswa Prakash Nayak, Md Fahad Shahariar Nayon, Athare Nazri-Panjaki, Masoud Negahdary, Ruxandra Irina Negoi, Ionut Negoi, Seyed Aria Nejadghaderi, Soroush Nematollahi, Samata Nepal, Henok Biresaw Netsere, Josephine W Ngunjiri, Cuong Tat Nguyen, Dang Nguyen, Trang Nguyen, Duc Hoang Nguyen, Phuong The Nguyen, Robina Khan Niazi, Luciano Nieddu, Mahdieh Niknam, Ali Nikoobar, Jan Rene Nkeck, Shuhei Nomura, Syed Toukir Ahmed Noor, Mamoona Noreen, Masoud Noroozi, Nawsherwan, Jean Jacques Noubiap, Mehran Nouri, Chisom Adaobi Nri-Ezedi, Fred Nugen, Dieta Nurrika, Ogochukwu Janet Nzoputam, Erin M O'Connell, Bogdan Oancea, James Odhiambo Oguta, In-Hwan Oh, Hassan Okati-Aliabad, Akinkunmi Paul Okekunle, Osaretin Christabel Okonji, Andrew T Olagunju, Oladotun Victor Olalusi, Timothy Olusegun Olanrewaju, Omotola O Olasupo, Gláucia Maria Moraes Oliveira, Arão Belitardo Oliveira, Yinka Doris Oluwafemi, Hany A Omar, Ahmed Omar Bali, Marcel Opitz, Michal Ordak, Alberto Ortiz, Augustus Osborne, Wael M S Osman, Alaa A M Osman, Uchechukwu Levi Osuagwu, Adrian Otoiu, Abdu Oumer, Amel Ouyahia, Mayowa O Owolabi, Irene Amoakoh Owusu, Kolapo Oyebola, Mahesh Padukudru P A, Alicia Padron-Monedero, Jagadish Rao Padubidri, Sujogya Kumar Panda, Songhomitra Panda-Jonas, Anamika Pandey, Seithikurippu R Pandi-Perumal, Shahina Pardhan, Utsav Parekh, Pragyan Paramita Parija, Romil R Parikh, Eun-Cheol Park, Ava Pashaei, Roberto Passera, Hemal M Patel, Aslam Ramjan Pathan, Dimitrios Patoulias, George C Patton, Susan Paudel, Hamidreza Pazoki Toroudi, Umberto Pensato, Prince Peprah, Gavin Pereira, Marcos Pereira, Arokiasamy Perianayagam, Norberto Perico, Simone Perna, Ionela-Roxana Petcu, Fanny Emily Petermann-Rocha, Hoang Nhat Pham, Roman V Polibin, Djordje S Popovic, Farzad Pourghazi, Akram Pourshams, Jalandhar Pradhan, Pranil Man Singh Pradhan, Manya Prasad, Akila Prashant, Elton Junio Sady Prates, I Gusti Ngurah Edi Putra, Jagadeesh Puvvula, Ibrahim Qattea, Jia-Yong Qiu, Venkatraman Radhakrishnan, Maja R Radojčić, Catalina Raggi, Muhammad Aziz Rahman, Fryad Majeed Rahman, Mohammad Hifz Ur Rahman, Mosiur Rahman, Saeed Rahmani, Vahid Rahmanian, Setyaningrum Rahmawaty, Rajesh Kumar Rai, Ivano Raimondo, Jeffrey Pradeep Raj, Prashant Rajput, Mahmoud Mohammed Ramadan, Chitra Ramasamy, Shakthi Kumaran Ramasamy, Sheena Ramazanu, Kritika Rana, Chhabi Lal Ranabhat, Mithun Rao, Sowmya J Rao, Sina Rashedi, Mohammad-Mahdi Rashidi, Ashkan Rasouli-Saravani, Devarajan Rathish, Santosh Kumar Rauniyar, Ilari Rautalin, David Laith Rawaf, Salman Rawaf, Elrashdy M. Moustafa Mohamed Redwan, Sanika Rege, Ana Reis-Mendes, Giuseppe Remuzzi, Nazila Rezaei, Mohsen Rezaeian, Hossein Rezazadeh, Taeho Gregory Rhee, João Rocha Rocha-Gomes, Mónica Rodrigues, Thales Philipe Rodrigues da Silva, Jefferson Antonio Buendia Rodriguez, Leonardo Roever, Peter Rohloff, Debby Syahru Romadlon, Moustaq Karim Khan Rony, Gholamreza Roshandel, Himanshu Sekhar Rout, Nitai Roy, Godfrey M Rwegerera, Aly M A Saad, Maha Mohamed Saber-Ayad, Cameron John Sabet, Kabir P Sadarangani, Basema Ahmad Saddik, Masoumeh Sadeghi, Mohammad Reza Saeb, Umar Saeed, Sahar Saeedi Moghaddam, Sher Zaman Safi, Amene Saghazadeh, Dominic Sagoe, Amirhossein Sahebkar, Fatemeh Saheb Sharif-Askari, Soumya Swaroop Sahoo, Mirza Rizwan Sajid, Luciane B Salaroli, Mohamed A Saleh, Marwa Rashad Salem, Sohrab Salimi, Yoseph Leonardo Samodra, Vijaya Paul Samuel, Abdallah M Samy, Prasanna K Santhekadur, Milena M Santric-Milicevic, Muhammad Arif Nadeem Saqib, Ushasi Saraswati, Aswini Saravanan, Dianis Wulan Sari, Tanmay Sarkar, Mohammad Sarmadi, Sachin C Sarode, Gargi Sachin Sarode, Michele Sassano, Brijesh Sathian, Ganesh Kumar Saya, Christophe Schinckus, Maria Inês Schmidt, Art Schuermans, Aletta Elisabeth Schutte, Sneha Annie Sebastian, Siddharthan Selvaraj, Mohammad H Semreen, Ashenafi Kibret Sendekie, Pallav Sengupta, Yigit Can Senol, Subramanian Senthilkumaran, Sadaf G Sepanlou, Yashendra Sethi, Allen Seylani, Mahan Shafie, Sweni Shah, Syed Mahboob Shah, Samiah Shahid, Fatemeh Shahrahmani, Moyad Jamal Shahwan, Sunder Sham, Muhammad Aaqib Shamim, Mehran Shams-Beyranvand, Anas Shamsi, Alfiya Shamsutdinova, Dan Shan, Mohd Shanawaz, Mohammed Shannawaz, Medha Sharath, Sadaf Sharfaei, Amin Sharifan, Anupam Sharma, Ujjawal Sharma, Manoj Sharma, Vishal Sharma, Fateme Sheida, Ali Sheikhy, Rekha Raghuveer Shenoy, Pavanchand H Shetty, Kenji Shibuya, Desalegn Shiferaw, Min-Jeong Shin, Rahman Shiri, Aminu Shittu, Sina Shool, Seyed Afshin Shorofi, Rajan Shrestha, Kerem Shuval, Yafei Si, Nicole R S Sibuyi, Emmanuel Edwar Siddig, Ahmed Kamal Siddiqi, Mithun Sikdar, Diego Augusto Santos Silva, Luís Manuel Lopes Rodrigues Silva, Surjit Singh, Jasvinder A Singh, Amit Singh, Harmanjit Singh, Baljinder Singh, Kalpana Singh, Puneetpal Singh, Valentin Yurievich Skryabin, Anna Aleksandrovna Skryabina, Amanda E Smith, Georgia Smith, Sameh S M Soliman, Soroush Soraneh, Reed J D Sorensen, Michael Spartalis, Bahadar S Srichawla, Panagiotis Stachteas, Antonina V Starodubova, Kurt Straif, Pete Stubbs, Vetriselvan Subramaniyan, Muritala Odidi Suleiman Odidi, Aleksander Sulkowski, Anusha Sultan Meo, Jing Sun, Zhong Sun, Sumam Sunny, Chandan Kumar Swain, Lukasz Szarpak, Rafael Tabarés-Seisdedos, Seyyed Mohammad Tabatabaei, Fatemeh Sadat Tabatabaei, Ozra Tabatabaei Malazy, Shima Tabatabai, Celine Tabche, Mohammad Tabish, Jabeen Taiba, Stella Talic, Mircea Tampa, Jacques Lukenze Tamuzi, Ker-Kan Tan, Manoj Tanwar, Saba Tariq, Nathan Y Tat, Seyed Mohammad Tavangar, Reem Mohamad Hani Temsah, Mohamad-Hani Temsah, Masayuki Teramoto, Dufera Rikitu Terefa, Jay Tewari, Rekha Thapar, Jansje Henny Vera Ticoalu, Sofonyas Abebaw Tiruneh, Tenaw Yimer Tiruye, Mariya Vladimirovna Titova, Krishna Tiwari, Sojit Tomo, Marcello Tonelli, Mathilde Touvier, Marcos Roberto Tovani-Palone, Khaled Trabelsi, Mai Thi Ngoc Tran, Thang Huu Tran, Nguyen Tran Minh Duc, Domenico Trico, Indang Trihandini, Thien Tan Tri Tai Truyen, Aristidis Tsatsakis, Gary Tse, Guesh Mebrahtom Tsegay, Munkhtuya Tumurkhuu, Sree Sudha T Y, Sok Cin Tye, Stefanos Tyrovolas, Aniefiok John Udoakang, Shahid Ullah, Saeed Ullah, Muhammad Umair, Umar Muhammad Umar, Lawan Umar, Brigid Unim, Dinesh Upadhya, Era Upadhyay, Jibrin Sammani Usman, Damla Ustunsoz, Asokan Govindaraj Vaithinathan, Jef Van den Eynde, Joe Varghese, Tommi Juhani Vasankari, Siavash Vaziri, Balachandar Vellingiri, Narayanaswamy Venketasubramanian, Madhur Verma, Anjul Verma, Georgios-Ioannis Verras, Simone Vidale, Victor E Villalobos-Daniel, Manish Vinayak, Vasily Vlassov, Theo Vos, Rade Vukovic, Mugi Wahidin, Mohammad Wahiduzzaman, Yanzhong Wang, Shu Wang, Cong Wang, Xingxin Wang, Mary Njeri Wanjau, Ahmed Bilal Waqar, Muhammad Waqas, Kosala Gayan Weerakoon, Fei-Long Wei, Anggi Lukman Wicaksana, Dakshitha Praneeth Wickramasinghe, Peter Willeit, Marcin W Wojewodzic, Tewodros Eshete Wonde, Utoomporn Wongsin, Qing Xia, Wanqing Xie, Suowen Xu, Xiaoyue Xu, Kazumasa Yamagishi, Yuichiro Yano, Haiqiang Yao, Amir Yarahmadi, Habib Yaribeygi, Subah Abderehim Yesuf, Dehui Yin, Dong Keon Yon, Naohiro Yonemoto, Chuanhua Yu, Chun-Wei Yuan, Deniz Yuce, Ismaeel Yunusa, Sojib Bin Zaman, Iman Zare, Michael Zastrozhin, Mohammed G M Zeariya, Xiaoyi Zhang, Liqun Zhang, Jingya Zhang, Zhiqiang Zhang, Casper J P Zhang, David X Zheng, Peng Zheng, Anthony Zhong, Claire Chenwen Zhong, Jiayan Zhou, Bin Zhu, Abzal Zhumagaliuly, Magdalena Zielińska, Ghazal Zoghi, Zhiyong Zou, Elric Zweck, Sa'ed H Zyoud, Christopher J L Murray, Susan M Sawyer, Stein Emil Vollset

## Abstract

**Background:**

Overweight and obesity is a global epidemic. Forecasting future trajectories of the epidemic is crucial for providing an evidence base for policy change. In this study, we examine the historical trends of the global, regional, and national prevalence of adult overweight and obesity from 1990 to 2021 and forecast the future trajectories to 2050.

**Methods:**

Leveraging established methodology from the Global Burden of Diseases, Injuries, and Risk Factors Study, we estimated the prevalence of overweight and obesity among individuals aged 25 years and older by age and sex for 204 countries and territories from 1990 to 2050. Retrospective and current prevalence trends were derived based on both self-reported and measured anthropometric data extracted from 1350 unique sources, which include survey microdata and reports, as well as published literature. Specific adjustment was applied to correct for self-report bias. Spatiotemporal Gaussian process regression models were used to synthesise data, leveraging both spatial and temporal correlation in epidemiological trends, to optimise the comparability of results across time and geographies. To generate forecast estimates, we used forecasts of the Socio-demographic Index and temporal correlation patterns presented as annualised rate of change to inform future trajectories. We considered a reference scenario assuming the continuation of historical trends.

**Findings:**

Rates of overweight and obesity increased at the global and regional levels, and in all nations, between 1990 and 2021. In 2021, an estimated 1·00 billion (95% uncertainty interval [UI] 0·989–1·01) adult males and 1·11 billion (1·10–1·12) adult females had overweight and obesity. China had the largest population of adults with overweight and obesity (402 million [397–407] individuals), followed by India (180 million [167–194]) and the USA (172 million [169–174]). The highest age-standardised prevalence of overweight and obesity was observed in countries in Oceania and north Africa and the Middle East, with many of these countries reporting prevalence of more than 80% in adults. Compared with 1990, the global prevalence of obesity had increased by 155·1% (149·8–160·3) in males and 104·9% (95% UI 100·9–108·8) in females. The most rapid rise in obesity prevalence was observed in the north Africa and the Middle East super-region, where age-standardised prevalence rates in males more than tripled and in females more than doubled. Assuming the continuation of historical trends, by 2050, we forecast that the total number of adults living with overweight and obesity will reach 3·80 billion (95% UI 3·39–4·04), over half of the likely global adult population at that time. While China, India, and the USA will continue to constitute a large proportion of the global population with overweight and obesity, the number in the sub-Saharan Africa super-region is forecasted to increase by 254·8% (234·4–269·5). In Nigeria specifically, the number of adults with overweight and obesity is forecasted to rise to 141 million (121–162) by 2050, making it the country with the fourth-largest population with overweight and obesity.

**Interpretation:**

No country to date has successfully curbed the rising rates of adult overweight and obesity. Without immediate and effective intervention, overweight and obesity will continue to increase globally. Particularly in Asia and Africa, driven by growing populations, the number of individuals with overweight and obesity is forecast to rise substantially. These regions will face a considerable increase in obesity-related disease burden. Merely acknowledging obesity as a global health issue would be negligent on the part of global health and public health practitioners; more aggressive and targeted measures are required to address this crisis, as obesity is one of the foremost avertible risks to health now and in the future and poses an unparalleled threat of premature disease and death at local, national, and global levels.

**Funding:**

Bill & Melinda Gates Foundation.

**Research in context**

**Evidence before this study**

Tracking and forecasting overweight and obesity trends are key to targeted prevention and intervention. We conducted a systematic literature search of Ovid MEDLINE and PubMed for articles published from inception up to April 30, 2024, using the terms “overweight and obese” AND “prevalence or epidemiology” AND “forecasting or projection” AND “adults” (and synonyms for each), with no language or year restrictions. We also searched the grey literature and the reference lists of relevant systematic reviews and meta-analyses. While many studies have been published on global or regional trends for overweight and obesity, those forecasting future scenarios are relatively sparse. Most studies (29 papers) were single-country projections, while ten papers outlined trends across multiple countries, but only four papers provided global forecasts for the adult population by country. One study in 2020, which projected the prevalence of obesity and diabetes across 185 countries, found that Libya, Kuwait, the UK, the USA, Argentina, and Nauru would have the highest prevalence of obesity in their respective continents by 2030. A 2008 study estimated that by 2030, the number of individuals with overweight and obesity would reach 1·35 billion due to population growth and urbanisation. It further suggested that if secular trends were accounted for, this number could rise to 2·16 billion. From the grey literature, using results from the NCD Risk Factor Collaboration group, the World Obesity Atlas 2023 report projected that, based on the current trajectory, by 2035, more than half the world's population—over 4 billion people—will be affected by overweight and obesity. To our knowledge, no study to date has generated an extended forecast until 2050.

**Added value of this study**

This study provides updated estimates of the global prevalence of overweight and obesity with forecasts until 2050. Using established methods from the Global Burden of Diseases, Injuries, and Risk Factors Study, we synthesised data from various sources to generate consistent estimates by age and sex for 204 countries and territories from 1990 to 2021. Based on the historical trends, we applied a generalised ensemble modelling approach to derive the forecasts of prevalence and examine the trajectory of overweight and obesity to 2050. In addition to comparing temporal and geographical variations in overweight and obesity prevalence, we examined differences in age patterns across birth cohorts, highlighting the evolution of the obesity pandemic through generations.

**Implications of all the available evidence**

Current strategies have failed to address the obesity crisis. Despite long-standing awareness of the threat to disease and premature death, no country has made substantial progress in reducing adult obesity. Prioritising healthy weight among populations is a leading global health challenge, and far more concerted efforts are required to deliver comprehensive interventions tailored to each country's unique sociodemographic, economic, environmental, and commercial contexts. Ongoing monitoring of overweight and obesity prevalence remains crucial for assessing the current status and future trajectory of the pandemic, as well as for providing key data to evaluate the effectiveness of interventions.

## Introduction

Overweight and obesity is a major health crisis and a real and present threat to global health progress.^1^ In 2021, 3·71 million deaths and 129 million disability-adjusted life-years (DALYs) were attributable to overweight and obesity.^2^ In the past two decades, the global age-standardised DALY rates associated with overweight and obesity increased by over 15%, placing it as one of the top risk factors, and the risk with the steepest increase in attributable burden.^2^

With ongoing surges in obesity among children and adolescents around the world,^3^ adult overweight and obesity is only expected to grow. Consequently, the burden of various non-communicable diseases (NCDs)—particularly diabetes, cardiovascular diseases, and cancer—will continue to escalate.^4^ A recent global forecasting study suggested that, driven by the ongoing obesity crisis, more than 1·31 billion people worldwide will develop diabetes by 2050.^5^ Moreover, country-specific forecasts predicted that the incidence of cardiovascular events will more than double in the next decade in some countries.^6^, ^7^ The number of obesity-related cancer cases is also expected to rise to over 2 million new cases globally by 2070, accounting for 7% of all cancer.^8^ Effective interventions to address obesity are urgently needed to avoid the foreseeable increases in these disease burdens.

Beyond adverse health outcomes, the global economic ramifications of overweight and obesity are equally staggering. In 2019, the estimated total costs associated with obesity, including both direct and indirect costs, ranged from US$3·19 billion in low-income countries to $1·33 trillion in high-income countries.^9^ Forecasts suggest that, by 2035, the obesity epidemic could lead to a 2·9% reduction in global gross domestic product, equating to a loss of $4 trillion.^10^ Addressing overweight and obesity is crucial not only for preserving population health but also for ensuring sustainable economic growth and development.

To support long-term policy planning and highlight the urgent need for action, it is essential to understand the current status and forecast future trajectories. Over the years, numerous studies have shown a relentless increase in the prevalence of overweight and obesity globally.^11^, ^12^, ^13^, ^14^ Nevertheless, comprehensive studies of prevalence forecasts are sparse.^10^, ^15^, ^16^ In this study, we leverage data from the Global Burden of Diseases, Injuries, and Risk Factors Study (GBD) 2021 to provide updated estimates of adult overweight and obesity prevalence by age and sex for 204 countries and territories from 1990 to 2021, with forecasts extending to 2050. Our forecast scenario offers an outlook based on the assumption that trends in overweight and obesity, as well as changes in policies and interventions, will continue according to their historical pace. This paper was produced as part of the GBD Collaborator Network and in accordance with the GBD Protocol.^17^

## Methods

### Overview

Based on the GBD 2021 study framework,^2^ we estimate and forecast the prevalence of overweight and obesity among adults aged 25 years and older from 1990 to 2050. The prevalence of overweight and obesity was stratified into 5-year age groups and by sex for 204 countries and territories. Overweight and obesity, based on both self-reported and measured data, are defined using BMI, calculated as mass in kilograms divided by the square of height in metres (kg/m²). A BMI of 25·0 or higher but less than 30·0 is classified as overweight, while a BMI of 30·0 or higher is classified as obese.^18^ Although BMI has its limitations as a measure of adiposity,^19^, ^20^ it is strongly associated with numerous health outcomes. It also remains the most commonly used metric for population-level assessments and provides the most extensive data. Regional and country-specific BMI cutoffs for defining overweight and obesity have previously been proposed.^21^ However, to ensure consistency in estimates over time and across geographies, an international standard is applied.

The results of this study are presented according to the GBD geographical definitions. The GBD framework classifies countries and territories into 21 regions and seven super-regions. Regions are defined by geographical proximity and epidemiological similarity. Regions are further grouped into super-regions based on cause of death patterns.^22^ The seven GBD super-regions are central Europe, eastern Europe, and central Asia; high income; Latin America and the Caribbean; north Africa and the Middle East; south Asia; southeast Asia, east Asia, and Oceania; and sub-Saharan Africa. This study complies with the Guidelines on Accurate and Transparent Health Estimates Reporting (appendix 1 pp 40–41).^23^ Analyses were completed with R version 4.4.0 and Python version 3.10. All codes used in the analysis are available upon request.

### Data sources

Data on overweight and obesity were identified through a systematic search through the Global Health Data Exchange (GHDx) appendix 1 p 3). Briefly, our inclusion criteria were limited to nationally or subnationally representative surveys at the state or province levels. Surveys targeting specific subpopulations were excluded. Studies involving self-reported or directly measured heights, weights, or BMI data were included in our analysis. Studies measuring overweight and obesity using alternative metrics, such as waist circumference and hip-to-waist ratio, were excluded due to the lack of reliable data for accurately converting these measurements into equivalent BMI-based prevalence estimates.

1350 data sources from 184 countries and territories between 1990 to 2021 were captured in our search. This included major multicountry survey programmes, such as the Demographic and Health Surveys, the WHO STEPwise Approach to Surveillance programme,^24^ the EU Eurobarometer Surveys,^25^ the UNICEF Multiple Indicator Cluster Surveys, the WHO World Health Surveys,^26^ the Centers for Disease Control and Prevention's Reproductive Health Surveys,^27^ the Survey of Health, Ageing and Retirement in Europe, and the International Social Survey Programme, along with various national multiyear surveys. Individual-level microdata or tabulated reports were extracted from these surveys for all ages. Any reported data with sample sizes smaller than ten were excluded. In addition, we conducted a systematic literature search to identify all published articles reporting on the prevalence of overweight and obesity based on BMI. Studies were included if the design consisted of a representative random sample of the population. Following data extraction, we performed rigorous quality checks to eliminate any duplications, inconsistencies, or implausible data entries. Further information on the search strategy, inclusion criteria, and data extraction methods are provided in previous publications.^11^, ^12^ The list of data sources can be accessed via the GHDx.

### Data standardisation

BMI calculated from measured height and weight is considered the reference or gold standard. To ensure consistency with this standard, adjustments were made to self-reported data to correct for potential biases. Distinct from previous GBD studies, a novel and more robust method based on meta-regression—Bayesian, regularised, trimmed (MR-BRT) was used.^28^ Briefly, using matched self-report and measured datapoints, sex-specific and super-region-specific MR-BRT models were developed to estimate bias correction factors. These bias correction factors were subsequently applied to all self-reported data. Details of the bias correction method can be found in appendix 1 (pp 7–10).

In addition to correcting for self-report bias, adjustments were made to standardise data reported in age intervals that differed from the conventional 5-year age grouping in GBD studies. Using the established GBD methodology,^11^, ^12^ an age–sex splitting model was derived using available survey microdata to approximate the underlying age–sex structure. Subsequently, aggregated prevalence values were redistributed into specific 5-year age and sex categories according to the estimated structure (appendix 1 p 7).

### Estimation of overweight and obesity prevalence from 1990 to 2021

Spatiotemporal Gaussian process regression (ST-GPR) was used to generate a complete retrospective time series for the prevalence of overweight and obesity by age, sex, and year for each country from 1990 to 2021. Although the current study reports only the prevalence among adults aged 25 years and older, the estimation includes all age groups, starting from age 5 years, to capture the underlying age patterns in overweight and obesity trends. A similar methodological approach was applied in previous studies.^11^, ^12^ Improvements in this iteration of GBD were as follows. An updated set of covariates, namely age-standardised educational attainment level and the proportion of the population working in agriculture, were incorporated into the linear model that served as an input for the mean function of ST-GPR. These new covariates proved to be more robust in capturing the relationship between socioeconomic development and overweight and obesity.^29^, ^30^ Separate models were applied to estimate prevalence in the USA to maximise the use of available country-level and state-level data and to better account for the unique obesity trends of the country.^31^ The results from the USA-specific model were subsequently corroborated and combined with those from the global model to generate the final estimates. Detailed descriptions of the models are available in appendix 1 (pp 8–10). 95% uncertainty intervals (UIs) for the final estimates were derived based on the 2·5th and the 97·5th percentiles of 1000 draws from the posterior distribution of ST-GPR. Further details can be found in previous publications.^11^, ^12^

### Forecasts of overweight and obesity prevalence from 2022 to 2050

Forecasted estimates of overweight and obesity prevalence from 2022 to 2050 were produced for a reference scenario, which is a probabilistic forecast of the most likely future based on past trends and relationships. Using prevalence estimates from 1990 to 2021 as inputs, a generalised ensemble modelling (GenEM) approach was applied to forecast the future prevalence of overweight and obesity, as well as the proportion of obesity among the population with overweight, from 2022 to 2050. The GenEM approach leverages the predictive strengths of 12 submodels. Six of these submodels were annualised rate of change (ARC) models with different recency weights, placing varying emphasis on recent year-over-year trends. The assumptions of our ARC submodels include the continuation of historical overweight and obesity trends, and relatedly that the historical pace of technological innovation will continue. For example, we have not built mass scale-up of GLP1 agonists or other rapid advancements in obesity prevention or treatment into our forecast. The remaining six submodels employed a two-stage MR-BRT spline model and used the Socio-demographic Index (SDI) as the covariate to inform trend prediction.^4^, ^32^, ^33^ In these MR-BRT models, we assume the past relationship between SDI and overweight and obesity will continue into the future. To select the best weights of submodels and validate the accuracy of forecast results, cross-validation based on a 10-year holdout period from 2012 to 2021 was used. The forecasted prevalence of obesity was then calculated by multiplying the forecasted prevalence of overweight and obesity by the forecasted proportion of obesity among the population with overweight for each draw. Similar approaches have been applied in previous publications.^4^, ^32^ Further details can be found in appendix 1 (pp 10–12). In addition to presenting the forecasted trend of prevalence over time and geography, we performed a cohort analysis by combining the forecasted 5-year age group prevalence from 2022 to 2050 with the estimates from 1990 to 2021. Age–period data were converted to age–cohort data, which enabled us to examine changes in age patterns and onset age by cohort. For instance, we compared the prevalence of obesity at age 25 years among the 1960, 1990, and 2015 cohorts to understand how the levels of prevalence differed. Additionally, we examined shifts in the peak prevalence across cohorts.

### Role of the funding source

The funder of this study had no role in study design, data collection, data analysis, data interpretation, or the writing of the report.

## Results

### Overweight and obesity prevalence in 2021

In 2021, an estimated 2·11 billion (95% UI 2·09–2·13) adults aged 25 years and older worldwide were affected by overweight and obesity—almost half of the total adult population (45·1% [44·7–45·4]). Of these individuals, approximately 1·00 billion (0·989–1·01) were males and 1·11 billion (1·10–1·12) were females. Eight countries—China, India, the USA, Brazil, Russia, Mexico, Indonesia, and Egypt—accounted for more than half of the global population living with overweight and obesity. The highest numbers were observed in China (402 million [397–407] individuals), India (180 million [167–194]) and the USA (172 million [169–174]; appendix 1 pp 85–112).

**Figure 1:**
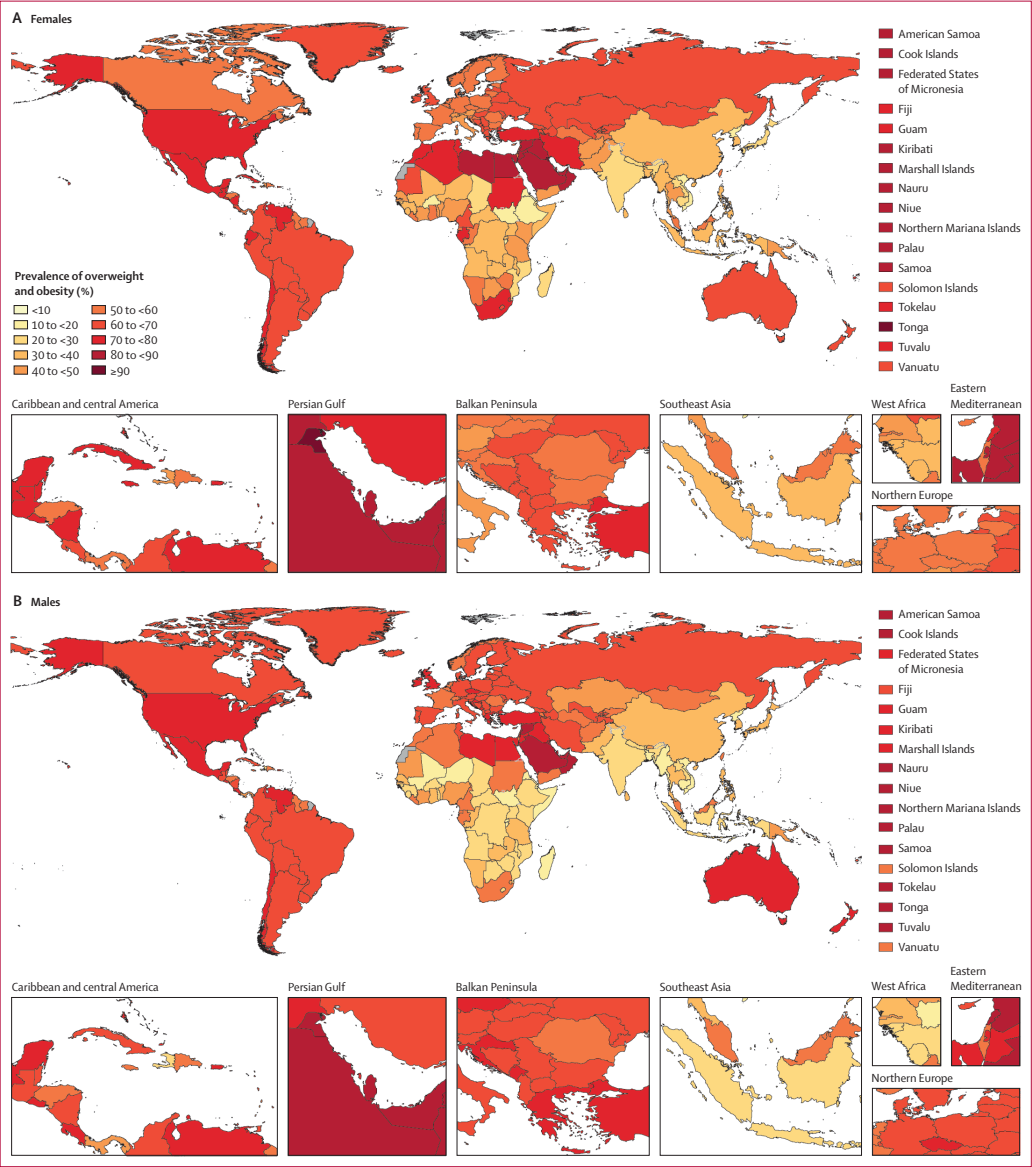
Estimated age-standardised prevalence of overweight and obesity among adults aged 25 years and older, by sex, 2021 (A) Females. (B) Males. No estimates are available for Western Sahara, French Guiana, or Svalbard, as these were not modelled locations in the Global Burden of Diseases, Injuries, and Risk Factors Study 2021.

**Figure 2:**
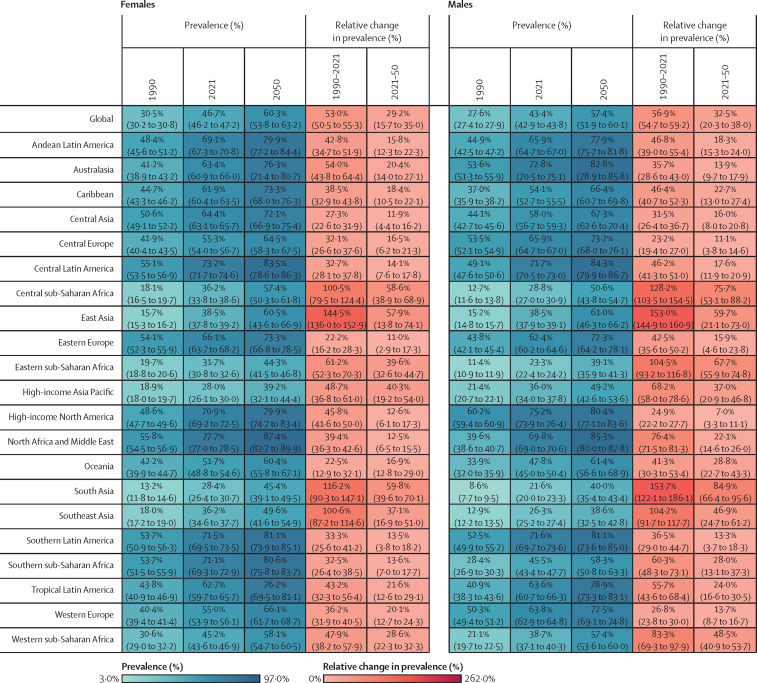

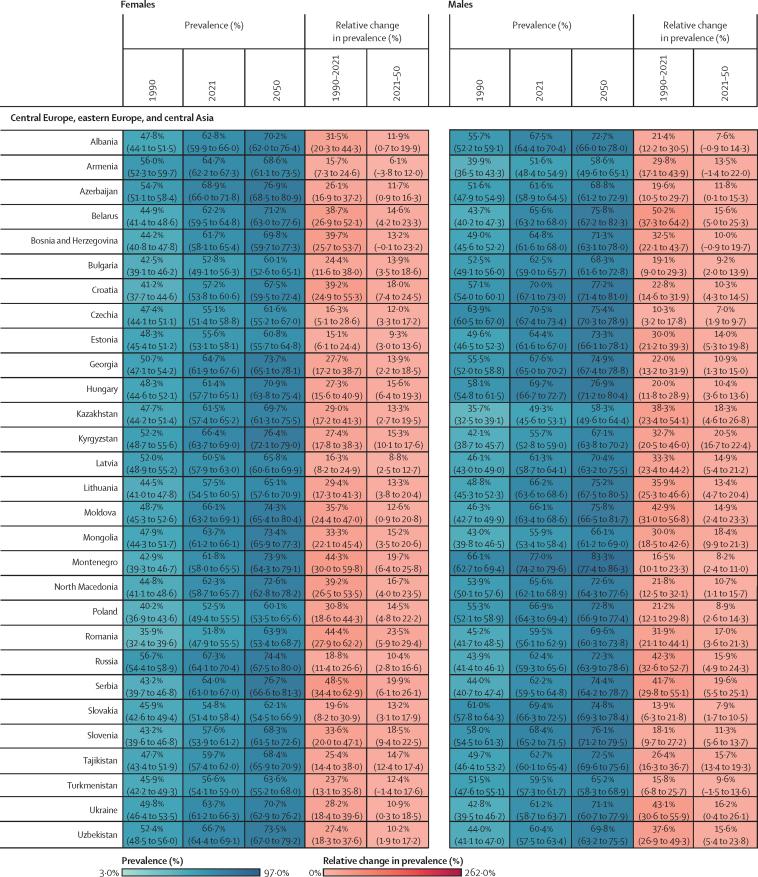

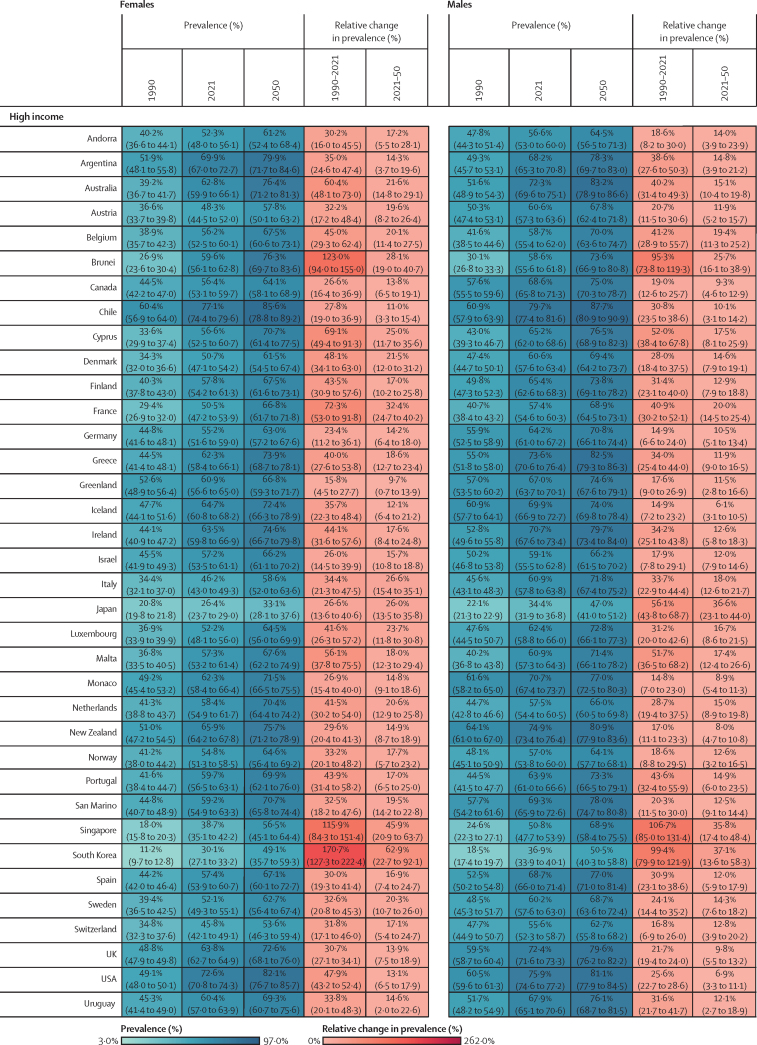

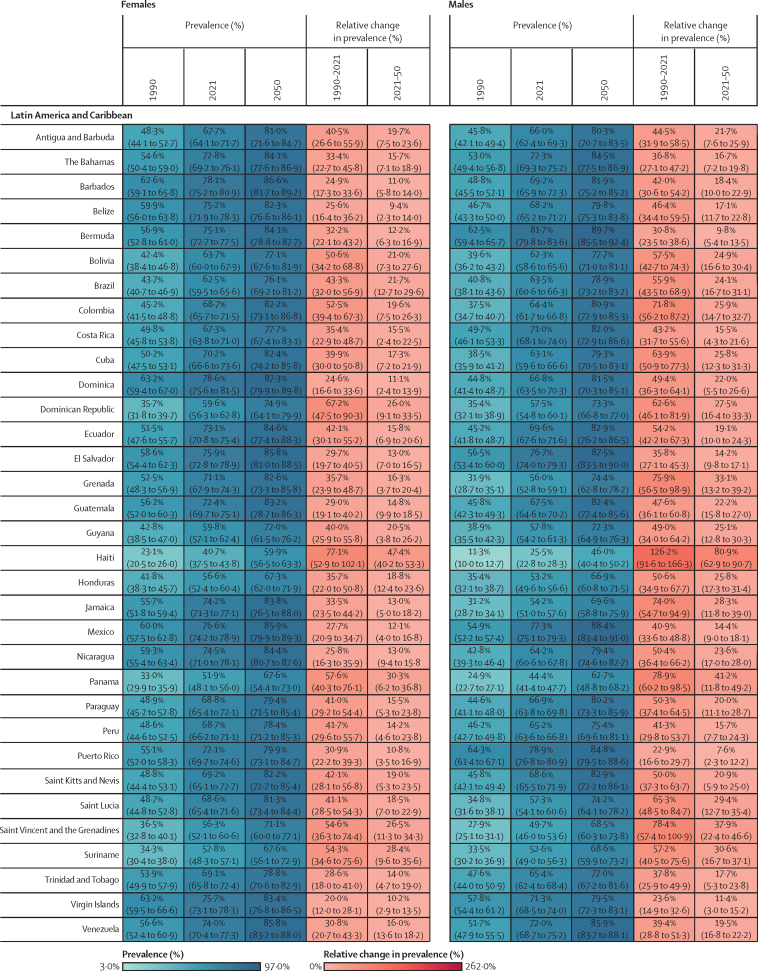

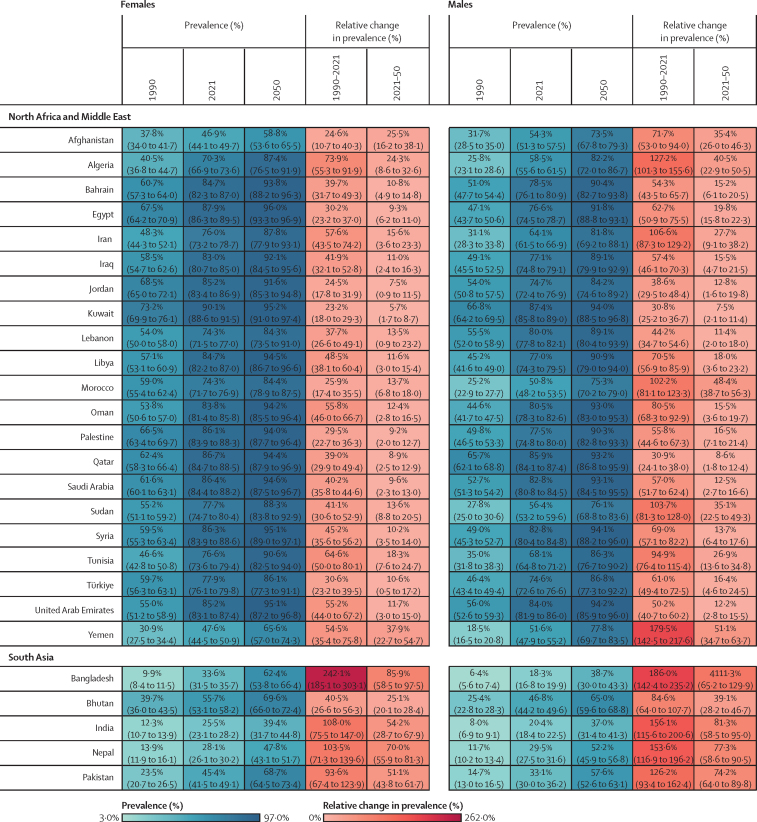

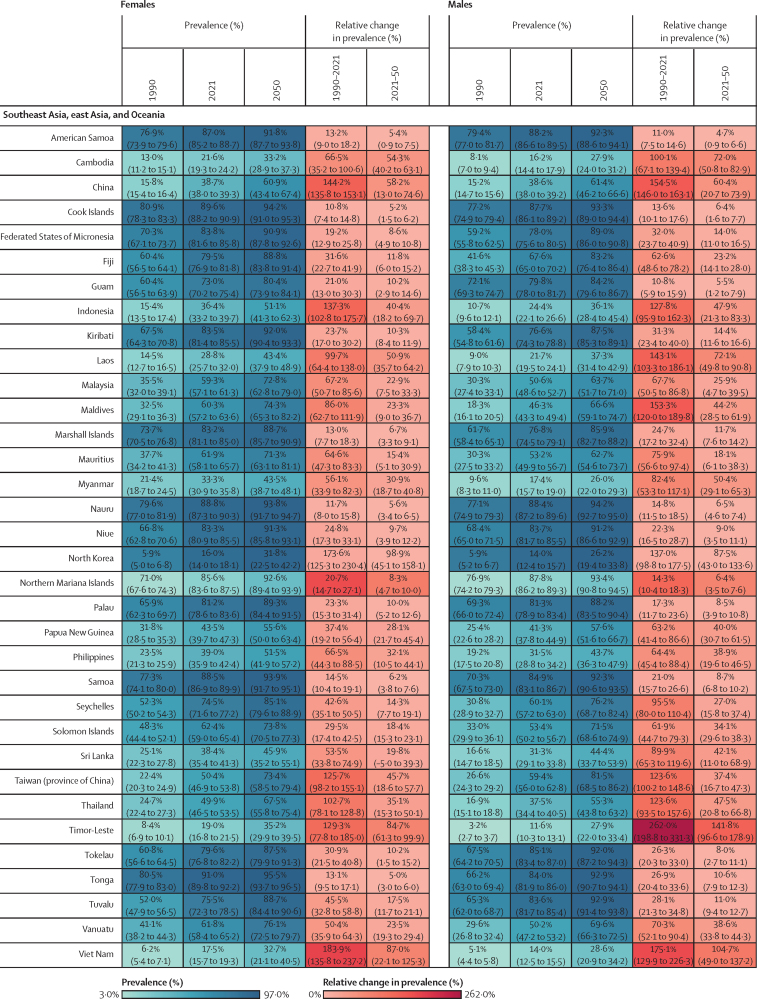

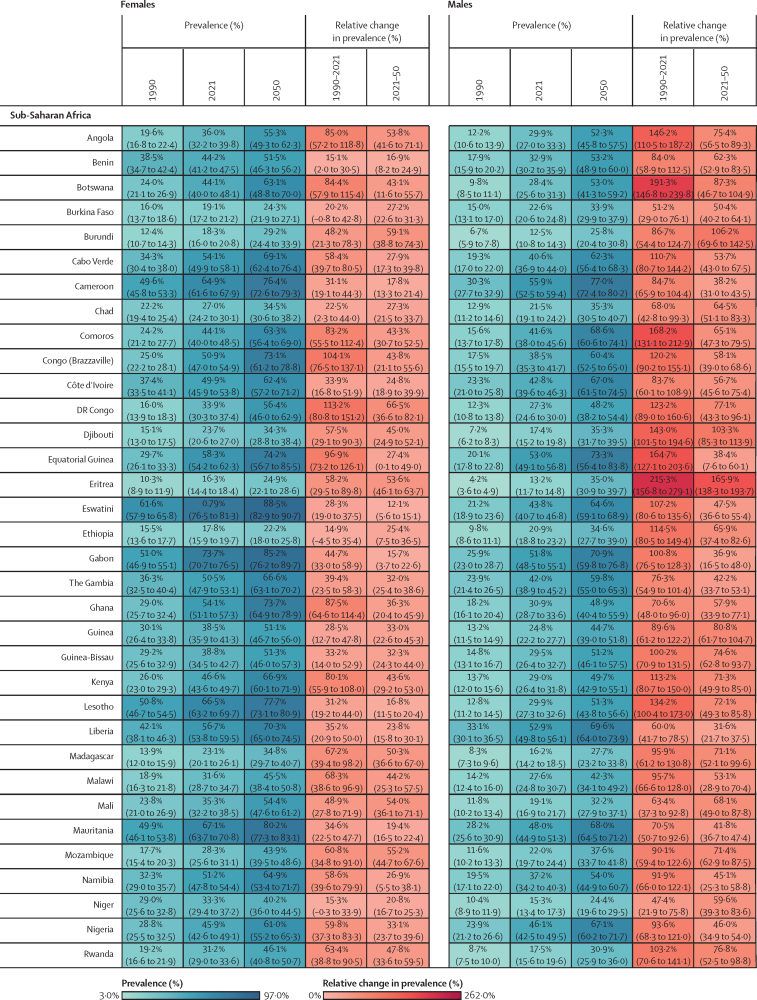

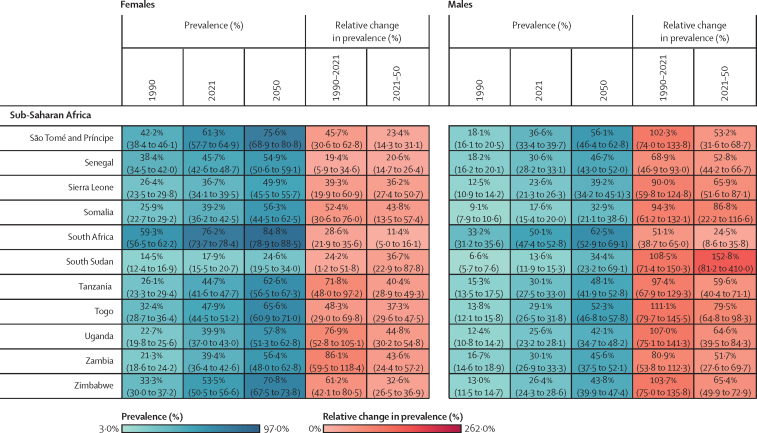
Estimated age-standardised prevalence of overweight and obesity and percentage changes among adults aged 25 years and older in 204 countries, by sex, 1990, 2021, and 2050 Values in parentheses are 95% uncertainty intervals.

Adjusting for demographic composition and age distribution shows the widespread nature of overweight and obesity around the world, with geographical variations observed in the age-standardised prevalence in 2021 (Figure 1, Figure 2). Nearly two-thirds of countries and territories, 133 of 204, recorded an age-standardised prevalence of over 50% for males and females. The countries and territories with the highest prevalence were primarily located in the regions of Oceania and north Africa and the Middle East. Among males, the prevalence of overweight and obesity was above 87% in Nauru, American Samoa, Northern Mariana Islands, Cook Islands, and Kuwait. Among females, the prevalence reached 88% and above in Tonga, Kuwait, Cook Islands, Nauru, and Samoa.

Focusing on obesity only, the highest age-standardised prevalence among males was observed in Nauru (67·0% [95% UI 64·6–69·2]), Cook Islands (65·7% [63·2–68·1]), and America Samoa (62·6% [59·9–65·2]; appendix 1 pp 44, 53). Among females, the age-standardised prevalence of obesity was highest in Tonga (76·3% [74·5–78·0]) and Nauru (71·6% [69·5–73·6]). Despite having one of the largest populations with overweight and obesity, China's age-standardised obesity prevalence was relatively low, estimated at 8·8% (8·6–9·1) for males and 10·8% (10·5–11·0) for females. Similarly, in India, the prevalence of obesity was estimated at 4·4% (3·8–5·2) for males and 7·5% (6·5–8·7) for females. By contrast, the USA had a relatively high prevalence of obesity, estimated at 41·5% (40·1–43·2) for males and 45·6% (43·7–47·5) for females, the highest among all high-income countries. In Latin America, 15 of the 17 countries had a prevalence of obesity among females of more than 30%, although the prevalence among males was slightly lower, with only seven countries exceeding 30%. At the regional level, the lowest prevalence of obesity among males was found in south Asia, estimated at less than 4·6% (4·1–5·2), whereas the lowest prevalence among females was in high-income Asia Pacific, estimated at 6·5% (5·9–7·1; appendix 1 p 53).

### Age and sex patterns in 2021

**Figure 3:**
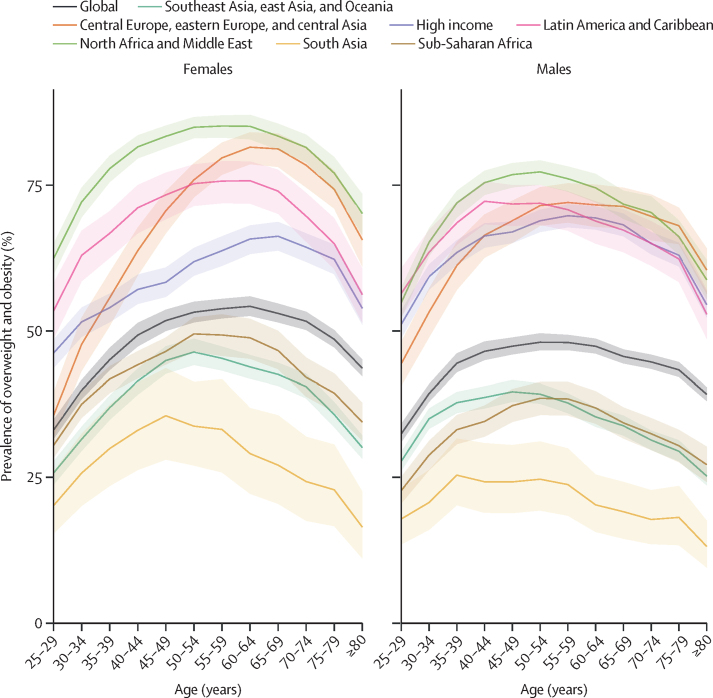
Estimated prevalence of overweight and obesity by age and sex, globally and by super-region, 2021 Shaded regions are 95% uncertainty intervals.

Figure 3 shows the age and sex patterns of overweight and obesity by GBD super-region. In 2021, the prevalence of overweight and obesity reached its highest point around age 50 years for both males and females in many GBD super-regions, including Latin America and the Caribbean; north Africa and the Middle East; southeast Asia, east Asia, and Oceania; and sub-Saharan Africa. By comparison, the prevalence of overweight and obesity in females peaked later in the central Europe, eastern Europe, and central Asia super-region (age 60–64 years) and high-income super-region (age 65–69 years), whereas among males, it reached its peak slightly earlier, at around age 55–59 years, in these two super-regions. In south Asia, the prevalence of overweight and obesity peaked at a younger age as compared with other super-regions, at 45–49 years for females and 35–39 years for males.

In all world super-regions except the high-income super-region, overweight and obesity prevalence was higher in females than in males (figure 3). In central Europe, eastern Europe, and central Asia, although the prevalence of overweight and obesity in males was higher than that in females before 40 years of age, the prevalence in females surpassed that of males after 40 years of age. A similar pattern was observed in southeast Asia, east Asia, and Oceania, where higher prevalence was observed in young adulthood in males, but by mid-adulthood, females overtook males. In north Africa and the Middle East, sub-Saharan Africa, and south Asia, the prevalence of overweight and obesity remained consistently high in females across the entire period of adulthood, with nearly parallel trends. In the high-income super-region, prevalence was consistently higher in males, with the gap between sexes narrowing only in late adulthood, close to age 65 years.

Conversely, for obesity alone, prevalence among females was consistently higher than that of males across all super-regions. However, the gap between sexes was more substantial in low-income and middle-income regions, such as sub-Saharan Africa and south Asia. In sub-Saharan Africa, in terms of relative percentage differences, the prevalence of obesity in females was over 100% higher than that in males for those aged 25–79 years. Between the ages of 50 years and 69 years, the relative percentage differences were over 140%. The smallest difference in obesity prevalence between males and females was observed in the high-income super-region, where the relative percentage difference was below 20% under age 70 years and increased moderately to 30–40% in older ages (appendix 1 p 48).

### Trends of adult obesity from 1990 to 2021

**Figure 4:**
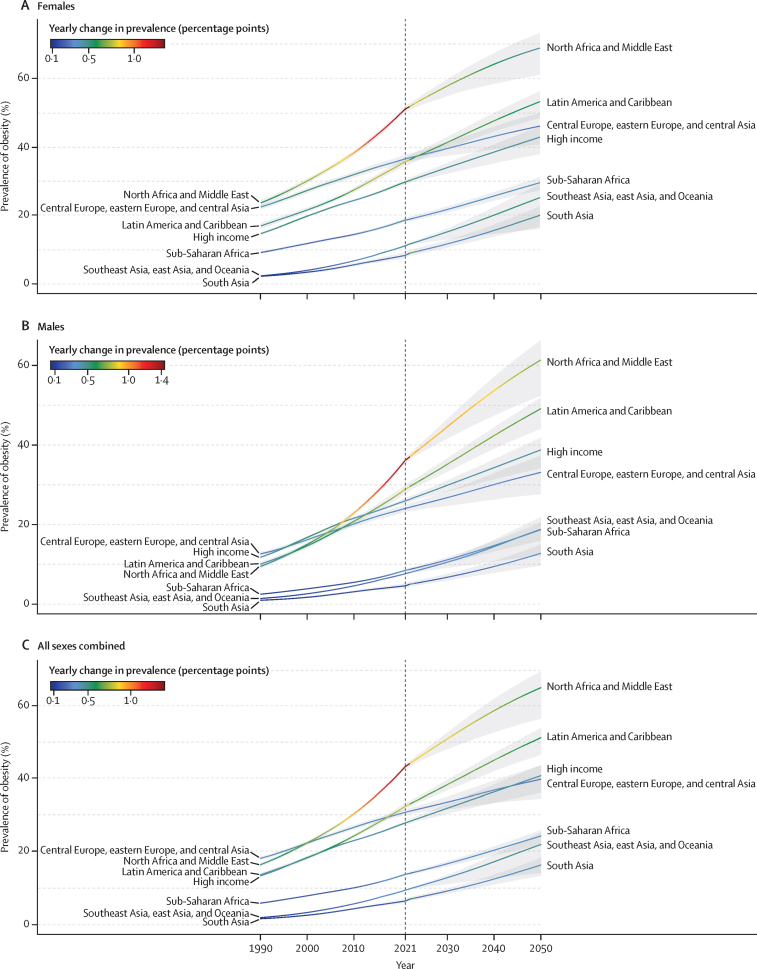
Estimated age-standardised prevalence of obesity in adults aged 25 years and older by sex, globally and by super-region, 1990–2050 (A) Females. (B) Males. (C) All sexes combined. Shaded regions are 95% uncertainty intervals.

Over the past three decades, the global prevalence of adult obesity has increased substantially—by 104·9% (95% UI 100·9–108·8) in females (from 10·2% [10·0–10·3] in 1990 to 20·8% [20·5–21·1] in 2021) and by 155·1% (149·8–160·3) in males (from 5·8% [5·7–5·9] to 14·8% [14·6–15·0]; appendix 1 p 53). Among the super-regions, north Africa and the Middle East had the sharpest increase: the obesity prevalence in males more than tripled, rising from 9·5% (9·0–9·9) in 1990 to 36·2% (35·4–36·9) in 2021, and in females it more than doubled, rising from 23·7% (22·8–24·5) to 51·1% (50·3–51·9). A comparison across super-regions is shown in figure 4 and appendix 1 (pp 51–52). The rate of increase was particularly rapid in the past decade, with annual absolute changes consistently above 1·0 percentage points among both males and females. A substantial rise was also observed in Latin America and the Caribbean, where the obesity prevalence among males increased from 10·0% (9·4–10·6) to 28·9% (27·8–30·0). In the high-income super-region and in central Europe, eastern Europe, and central Asia, despite slower rates of change compared with other super-regions, the prevalence of obesity rose steadily, with an annual absolute increase between 0·3 percentage points and 0·5 percentage points. Specifically, in the high-income super-region, obesity prevalence among males increased from 11·8% (11·5–12·0) in 1990 to 26·0% (25·4–26·5) in 2021, and among females from 14·7% (14·4–15·0) to 29·8% (29·1–30·4). In central Europe, eastern Europe, and central Asia, obesity prevalence climbed from 12·6% (12·2–13·1) to 24·1% (23·2–25·0) during the same period in males and from 22·5% (21·7–23·2) to 36·5% (35·6–37·7) in females. Additional results on the changes in overweight and obesity prevalence can be found in appendix 1 (pp 45–47, 49–50).

### Forecasts of adult overweight and obesity in 2050

Assuming a reference scenario in which historical trends and patterns continue into the future, we forecast that by 2050, the total number of individuals over 25 years of age with overweight and obesity will rise to 3·80 billion (95% UI 3·39–4·04) globally, among which 1·95 billion (1·64–2·13) would have obesity. China (with 627 million [448–736] affected individuals), India (450 million [359–535]), and the USA (214 million [196–231]) would continue to be the three countries with the largest numbers of people with overweight and obesity (appendix 1 p 54). Furthermore, driven by population growth, the largest increase in the number of individuals with overweight and obesity is expected in sub-Saharan Africa, with a forecasted increase of 254·8% (234·4–269·5) for this super-region. In Nigeria, the number of individuals with overweight and obesity is forecasted to increase by 287·4% (256·7–308·4), rising from 36·6 million (34·5–38·6) in 2021 to 141 million (121–162) in 2050, making it the country with the fourth-largest population with overweight and obesity (appendix 1 pp 54, 85–112).

The age-standardised prevalence of overweight and obesity is forecasted to increase by 30·7% (95% UI 17·8–36·3) globally over the next 30 years, with nearly 60% of adults expected to have overweight and obesity by 2050 (figure 2; appendix 1 pp 49–50). 60 countries and territories are forecasted to have an overweight and obesity prevalence over 80% among females, with 22 of these countries expected to exceed 90%. Among males, 54 countries and territories are forecasted to have an overweight and obesity prevalence above 80%, with 19 countries having a prevalence exceeding 90%. Countries in Oceania and north Africa and the Middle East will continue to have the highest prevalence of overweight and obesity. Among females, the highest prevalence is forecasted in Egypt (96·0% [93·3–96·9]), followed by Tonga (95·5% [93·7–96·5]). For males, the highest prevalence is forecasted in the United Arab Emirates (94·2% [85·9–96·0]), followed by Nauru (94·2% [92·7–95·0]). The largest increases in age-standardised rates of overweight and obesity prevalence are forecasted to be in south Asia, east Asia, and central and eastern sub-Saharan Africa. Among females, prevalence is forecasted to increase by 59·8% (39·6–70·1) in south Asia, 58·6% (38·9–68·9) in central sub-Saharan Africa, and 57·9% (13·8–74·1) in east Asia. The increase is more pronounced among males, with a forecasted increase of 84·9% (66·4–95·6) in south Asia, 75·7% (53·1–88·2) in central sub-Saharan Africa, and 67·7% (55·9–74·8) in eastern sub-Saharan Africa.

Specific to obesity, age-standardised prevalence is forecasted to increase by 68·3% (95% UI 42·6–80·5) globally, with approximately 1 in 3 adults over the age of 25 expected to experience obesity by 2050, among whom about a quarter will be over the age of 65 (see appendix 1 pp 51–53). 44 countries and territories are forecasted to have obesity prevalence exceeding 50% among males by 2050, and 45 countries and territories are forecasted to have obesity prevalence exceeding 60% among females. Tonga is forecasted to have the highest obesity prevalence among females, estimated at 87·7% (84·9–89·1), followed by Egypt at 87·0% (82·6–89·0). Among males, the highest obesity prevalence is projected in the United Arab Emirates at 81·1% (62·6–86·8), followed by Nauru at 80·3% (75·0–82·5). Obesity prevalence among males is projected to increase by over 100% in 71 countries and territories, while among females, it is forecasted to increase by over 100% in 24 countries and territories. These increases are predominantly expected in southeast Asia, east Asia, south Asia, and sub-Saharan Africa.

### Cohort pattern from 1990 to 2050

**Figure 5:**
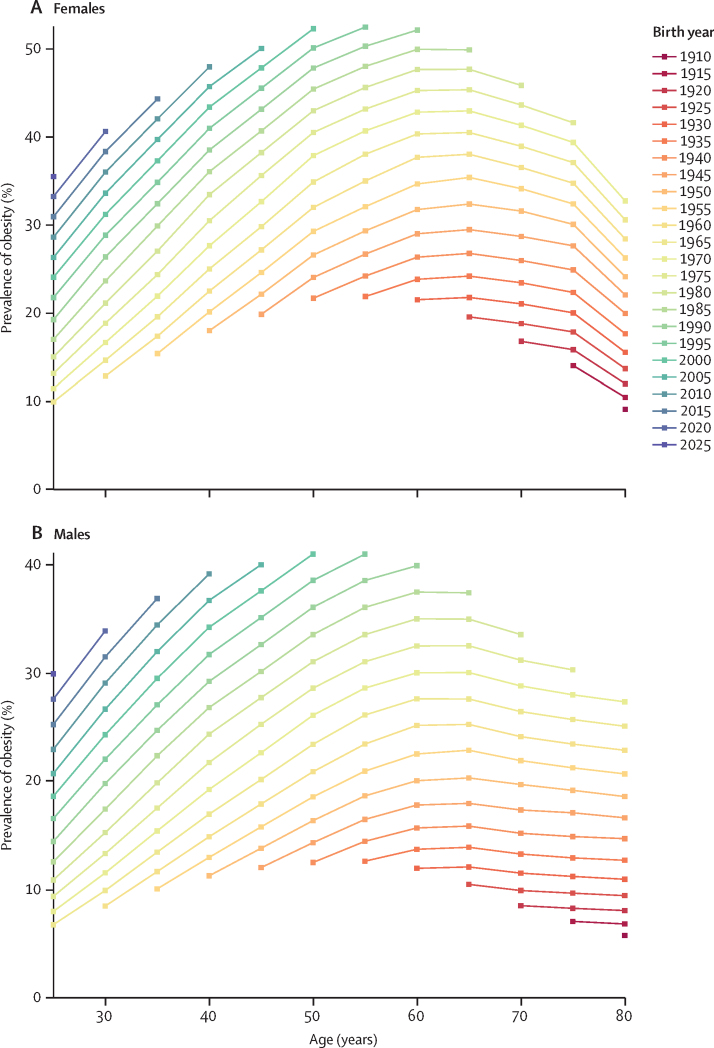
Estimated prevalence of obesity by age across birth cohorts globally, by sex (A) Females. (B) Males.

We synthesised forecast estimates, age patterns, and cohort information to illustrate the trends and trajectories of obesity prevalence across successive cohorts (ie, generations) globally and by super-region (figure 5; appendix 1 pp 71–84). Across all super-regions, the prevalence of obesity at a given age increases with each successive cohort. In the high-income super-region, at age 25 years, 7·1% (95% UI 4·6–10·2) of males in the 1960 cohort had obesity, compared with 16·3% (11·4–22·1) of the 1990 cohort and the forecasted 25·1% (17·2–33·7) of the 2015 cohort. Similarly, among females, at age 25 years, only 8·4% (5·4–12·1) of the 1960 cohort had obesity, compared with 18·9% (13·3–25·5) of the 1990 cohort and the forecasted 28·4% (19·7–37·9) of the 2015 cohort. The super-region with the most marked differences between cohorts is south Asia, where, between the 1960 cohort and the 2015 cohort, the prevalence of obesity at age 25 years is expected to increase by a relative 1116·6% among males and 719·9% among females. Sub-Saharan Africa also shows marked cohort differences, where the prevalence of the 2015 cohort in males at age 25 years is expected to be 962·0% higher than that of the 1960 cohort.

In addition to the increase in age-specific prevalence over successive cohorts, each cohort also reached the peak prevalence of the previous cohort at an earlier age. In the Latin America and the Caribbean super-region, the prevalence of obesity among males peaked at age 65 years in the 1980 cohort. However, the same level of prevalence was reached by age 45 years in the 1995 cohort and is expected to be exceeded by age 35 years in the 2010 cohort. Among females, the prevalence of obesity peaked at age 60 years in the 1980 cohort, and the same peak is expected to be exceeded by the 1995 cohort at age 50 years and by the 2010 cohort by age 40 years. Similar patterns were observed in other super-regions where the peak of obesity in earlier cohorts was matched or surpassed by subsequent cohorts 10–30 years earlier in age. A similar illustration for overweight and obesity prevalence cohort trends can be found in appendix 1 (pp 55–70).

## Discussion

This study offers an updated analysis of current and forecast trends in the prevalence of overweight and obesity by age and sex across 204 countries and territories from 1990 to 2050. Despite universal recognition of the threats posed by overweight and obesity, prevalence continues to rise globally, affecting adults of every age group and sex over the past three decades. Our reference scenario forecasts that by 2050, almost 2 in 3 adults over the age of 25 years will have overweight and obesity. While countries currently battling the obesity epidemic will continue to grapple with the crisis, greater burdens are expected to emerge in parts of Asia and sub-Saharan Africa. Driven by population growth, the number of adults with overweight and obesity is expected to double in some countries in these regions in the next 30 years.

Adult obesity is closely tied to childhood obesity.^34^ With the global prevalence of obesity in children and adolescents having increased by 244% in the past 30 years and having a forecasted increase of 121% in the next 30 years,^3^ trends in adult obesity prevalence are unlikely to abate. Our cohort analysis revealed steady increases in the prevalence of obesity with successive cohorts and a shift towards earlier onset. The rapid increases in obesity at younger ages for each cohort implies a heightened risk of early onset of a variety of complications, including type 2 diabetes, cardiovascular diseases, and certain cancers.^35^, ^36^ The surge in these complications impedes progress in population life expectancy and healthy life expectancy (HALE).^37^ Such negative consequences have already been observed in some countries, including Australia, the USA, and countries in Europe.^38^, ^39^, ^40^ In the USA, estimates in 2021 showed that the gap between life expectancy and HALE exceeded 12 years, which translates to over 16% of the expected life-years being lived in poor health.^41^ Without taking effective measures, these gaps will only widen.

In many countries, especially high-income countries (HICs), the rise of obesity is coupled with an ageing population and a low fertility rate, which together exert unparalleled pressure on existing health-care services and expenditures.^9^, ^42^ Our results indicate that by 2050, close to a quarter of the world's population with obesity will be older than 65 years. Beyond regular chronic condition management and geriatric care, ageing patients with obesity generally have higher demands for elective surgeries.^43^ They also tend to incur added costs due to suboptimal outcomes.^44^, ^45^ These surgical care costs constitute a substantial portion of health-care expenditure.^46^, ^47^ Obesity also increases risk of infections, resulting in health-care utilisation and costs that could potentially be avoided.^48^ The recent COVID-19 pandemic exposed the disproportionate impact on patients with obesity.^49^, ^50^ In the midst of escalating health-care service demand and expenditure driven by obesity,^9^, ^42^ reduced fertility and changing population age structure pose serious concerns for health-care system financing and service provision capacity. Traditional health financing mechanisms, which relied on labour markets as primary funding sources, are no longer sustainable given a shrinking workforce.^51^ Concurrently, an ageing health-care workforce with severe staff shortages is unable to meet the surge in service demands.^52^, ^53^ Without swift action, the growing obesity trends will further intensify the strain on health-care systems in most countries.

In low-income and middle-income countries (LMICs), the increase in obesity, combined with persistent childhood malnutrition and pervasive infectious diseases, creates a challenging epidemiological landscape that threatens to cripple the health-care systems in these already resource-scarce areas.^54^ As highlighted in the 2019 *Lancet* Commission on the syndemic of undernutrition, overnutrition, and climate change,^55^ many LMICs, including those in sub-Saharan Africa, south Asia, southeast Asia, and in the Pacific, have a double burden of malnutrition, with a high prevalence of undernutrition, particularly among children, and a growing prevalence of overweight and obesity among adults.^56^ Childhood undernutrition triggers physiological adaptations that stimulate energy accumulation in adipose tissue later in life, leading to obesity in adulthood.^57^, ^58^ This pattern of life-course exposure to the double burden of malnutrition triggers a metabolic capacity–load mismatch, which aggravates the risk of NCDs.^59^ Furthermore, obesity exacerbates individuals' susceptibility to infectious diseases and increases the risk of associated metabolic complications.^60^ Overall, the disease burden associated with NCDs has risen considerably in many LMICs in the past 30 years.^61^ Between 1990 and 2021, the prevalence of diabetes increased by more than 90% in sub-Saharan Africa and south Asia.^5^ During the same period, the number of DALYs associated with NCDs increased by over 80% in the two regions.^41^ Compared with infectious diseases and maternal and child health, NCDs have traditionally not been a priority area in LMICs and substantial gaps exist in essential NCD services.^62^ A recent study showed that the diagnosis and treatment rates of diabetes in sub-Saharan Africa and south Asia are among the lowest in the world (Stafford L, Institute for Health Metrics and Evaluation, personal communication). Curbing the obesity epidemic is therefore imperative for mitigating the substantial pressure faced by the existing health-care systems.

The forecasted rise in overweight and obesity is set to intensify existing health inequities globally. A comprehensive analysis of the dynamics between overweight and obesity inequities and socioeconomic, sex, racial, and ethnic disparities is beyond the scope of this study. However, our findings align with observations from published literature, which showed substantial geographical heterogeneity.^13^, ^14^ Such heterogeneity extends to the subnational level,^63^, ^64^ where socioeconomic factors interact with individuals' sex, race, ethnicity, and other characteristics, yielding a complex interplay of obesity risk across subpopulations.^65^ Therefore, it is necessary to understand local determinants of obesity in order to tailor appropriate interventions. Specific to sex disparities, our forecasts indicate that females will continue to have a higher prevalence of overweight and obesity than males across all super-regions except the high-income super-region, hinting to the potential widening of inequities in obesity-related health burden between the sexes.^66^ Considering that females often face suboptimal outcomes in many conditions, particularly in Africa and Asia, where the number of people with overweight and obesity is projected to increase substantially,^67^, ^68^ any obesity policies and interventions must recognise and address sex differences.^69^ Without targeted interventions to alleviate disparities in overweight and obesity, existing health inequities will be further amplified.

The global obesity epidemic is driven by a multitude of systemic factors and have been well discussed.^55^, ^70^ In addition to socioenvironmental factors, commercial determinants are essential in driving the obesity pandemic.^71^ Agricultural subsidies have been transforming the global food production and supply ecosystem, changing dietary content across all populations.^72^ Meanwhile, multinational food and beverage corporations and fast-food chains are shifting their investments from HICs to LMICs, where population growth, improvement in per-capita income, and weaker regulations have created favourable markets for expansion.^73^ Between 2009 and 2019, the largest annual growth in ultra-processed food and beverage sales per capita was observed in Cameroon, India, and Viet Nam.^74^ In addition to dietary impact, commercial determinants have modified other lifestyle choices, shaped built environments, and affected policy and legislation.^75^ These social, environmental and commercial influences, coupled with genetic predisposition, disproportionately affect certain populations,^76^ such as Caribbean and Pacific Islander populations, where over 80% of adults have overweight and obesity. In the past three decades, Africa and Asia have observed the largest percentage increases in obesity prevalence in the world. In these regions, the peak ages of obesity prevalence also appear to be younger compared with those in other regions. The exact reasons for some of the regional differences in age patterns are yet to be fully understood.

Undoing the harmful impacts of these factors requires a concerted multisectoral effort involving strong international governance to enforce coherent public policy, protect public policy space, and derive innovative solutions to incentivise alternative commercial practices conducive to promoting public health and interest.^77^ Although obesity has been on the global health agenda for over two decades,^78^ the translation of strategic plans into action has been limited in many countries, and progress has been minimal.^79^, ^80^ According to WHO, in 2021, only 40% of countries had an operational policy, strategy, or action plan for addressing overweight and obesity. In low-income countries, this policy coverage dropped to below 10%.^81^ A major challenge in implementing obesity intervention policies has been the identification and adaptation of strategies in real-world settings.^82^ Nevertheless, recent examples, such as New York City's ban on artificial trans fats^83^ and the taxation of sugar-sweetened beverages in the UK and the USA,^84^, ^85^ demonstrated the potential of promising policy interventions.^86^, ^87^, ^88^ Still, most obesity intervention studies have been conducted in high-income settings,^89^ and evidence on effective obesity intervention implementation in LMICs is scarce.^90^ With the rapid increase in the prevalence of overweight and obesity forecasted in many LMICs, it is imperative to ensure proper integration of monitoring and evaluation strategies alongside the introduction of new policies and programmes to accumulate evidence to support continuous policy planning and reform.^91^

Besides public health policies, anti-obesity medications have recently come into the spotlight with the approval of new-generation pharmacological options.^92^ The use of anti-obesity medications for weight control is nothing new. Various pharmacotherapies for obesity have been launched in the past;^93^ however, uptake was slow^94^ due to the risk of serious side-effects^95^, ^96^ and contraindications for conditions such as cardiovascular diseases, which are common among individuals with obesity.^97^ The new-generation anti-obesity medications have mitigated some of these constraints and appear to carry the potential to reach a wider population.^98^ However, the complex and heterogeneous nature of the biological mechanisms behind obesity means that treatment efficacy varies across individuals.^99^, ^100^ Access and cost are other key considerations. Anti-obesity medications are not readily available in many LMICs,^101^ and the cost of treatments is high.^102^ An attempt was made in 2023 to place anti-obesity medications on WHO's essential medicines lists to eliminate access barriers, but was rejected due to uncertain long-term clinical benefits and safety.^103^, ^104^ Although market exclusivity for several GLP-1 receptor antagonists anti-obesity medications is soon to expire and lower-cost generic versions are expected to become available, potentially broadening access,^105^ given the uncertainty in long-term outcomes,^95^, ^96^ the sustainability and scalability of anti-obesity medications as a remedy to the global obesity epidemic are doubtful; public health interventions will remain key strategies in tackling the crisis.^106^

The findings of this study should be interpreted while considering its limitations. First, the definition of overweight and obesity is based on BMI. While BMI is a convenient and by far the most abundant measure of adiposity, it does not account for variations in body structure across ethnic groups and subpopulations.^19^, ^20^ A recent, global, population-based study showed regional variations in the correlation between BMI and abdominal adiposity.^107^ Moreover, the use of universal cutoffs might lead to underestimations of overweight and obesity prevalence in certain countries such that, if corrected, the outcome would be more stark.^21^, ^108^ Second, to maximise data volume, self-reported data were included to supplement measured data. Self-reported heights and weights are prone to biases, and the extent of these biases differs by sex and country and evolves over time. We attempted to adjust for these biases with updated bias correction models that account for variations in sex and super-region. Despite our best efforts, it is probable that the bias correction remains imperfect. Third, subnational variations are not considered in this study. As documented in the literature, considerable differences exist in overweight and obesity prevalence across socioeconomic statuses, as well as racial and ethnic groups within countries.^109^, ^110^, ^111^ The estimates in this study, which aim to be representative at the national level, do not reflect subnational variations. This is an important area for future research. Fourth, due to data sparsity, the prevalence estimates and forecasts for certain countries and years rely more heavily on extrapolations influenced by the covariates in the model. The accuracy of these extrapolated estimates depends on the quality of the input covariate sources. Fifth, the age–cohort analysis was derived from age–period data, limited to the period between 1990 and 2050. Due to this constraint, prevalence estimates at certain ages for some cohorts were not captured. Consequently, the rise in obesity driven by changes during earlier years was not fully reflected. Finally, the current study only considered the reference scenario, which assumes the continuation of existing trends. Other scenarios examining the potential impact of interventions, such as scale-up of anti-obesity medications, were not included due to the lack of reliable long-term effect size information. With a better understanding of the effectiveness and sustainability of obesity intervention solutions, future studies can develop more robust forecasts to examine the relative impacts of different strategies.

Without drastic intervention, 3·80 billion adults over the age of 25 years will have overweight and obesity by 2050. This polycrisis will cause more avertable adverse health outcomes in the coming decades than any other modifiable risk at an individual level. Preventive measures are urgently needed, particularly in regions such as Asia and sub-Saharan Africa, where a surge in the number of individuals with overweight and obesity is anticipated, and existing health-care systems are ill-equipped for the rapid escalation of NCDs. Urgent, bold, and comprehensive initiatives are imperative to enable multisectoral collaboration and propel structural reforms to address drivers of overweight and obesity at individual and population levels. Although new generation anti-obesity medications appear promising, tactful, whole-system, public health strategies will continue to be crucial to achieving widespread and sustainable impact.

### GBD 2021 Adult BMI Collaborators

### Affiliations

### Contributors

### Data sharing

To download the input data used in these analyses, please visit the Global Health Data Exchange GBD 2021 website. All results from this study are publicly accessible. To download estimates produced in these analyses, please visit the GBD Results tool.

## Declaration of interests

Q E S Adnani reports grants or contracts from Online Library Data Research funds from Universitas Padjadjaran, Bandung, Indonesia, under contract number 2152/UN6.3.1/PT.00/2024 and Acceleration to Associate Professor Grant from Universitas Padjadjaran, Bandung, Indonesia under contract number 1592/UN6.3.1/PT.00/2024, outside the submitted work. S Afzal reports support for their support in the present manuscript from King Edward Medical University; payment or honoraria for lectures, presentations, or educational events from King Edward Medical University and collaborative partners including University of Johns Hopkins, University of California, University of Massachusetts, KEMCAANA, KEMCA_UK international scientific conferences, webinars and meetings; support for attending meetings and/or travel from King Edward Medical University; participation on a data safety monitoring board or advisory board with National Bioethics Committee Pakistan, King Edward Medical University Ethical Review Board, Ethical Review Board Fatima Jinnah Medical University and Sir Ganga Ram Hospital, Member Technical Working Group on Infectious Diseases to formulate guidelines; leadership or fiduciary role in other board, society, committee or advocacy group (paid or unpaid) Pakistan Association of Medical Editors, Fellow of Faculty of Public Health Royal Colleges UK (FFPH), Society of Prevention, Advocacy And Research, King Edward Medical University (SPARK), Member Pakistan Society of Infectious Diseases; other financial or non-financial interests through serving as Dean of Public Health and Preventive Medicine King Edward Medical University, Chief Editor Annals of King Edward Medical University since 2014, Director Quality Enhancement Cell King Edward Medical University, Fellow of Faculty of Public Health United Kingdom. Advisory Board Member and Chair Scientific Session, KEMCA-UK, Chairperson International Scientific Conference, KEMCAANA, At National level, Member Research and Publications Higher Education Commission, HEC Pakistan, Member Research and Journals Committee Pakistan Medical and Dental Council, Pakistan Member National Bioethics Committee, Pakistan At Punjab Level, Member Corona Experts Advisory Group. Member Technical Working Group on Infectious Diseases to formulate guidelines Member Dengue Experts Advisory Group Chair, Punjab Residency Program Research Committee; all outside the submitted work. S M Alif reports Payment or honoraria for lectures, presentations, speakers bureaus, manuscript writing or educational events from Victoria University Online; support for attending meetings and/or travel from University of Melbourne; leadership or fiduciary role in other board, society, committee or advocacy group (paid or unpaid), from Thoracic Society of Australia and New Zealand; all outside the submitted work. R Ancuceanu reports Consulting fees from AbbVie and Merck Romania; payment or honoraria for lectures, presentations, speakers bureaus, manuscript writing or educational events from AbbVie, Laropharm, Reckitt, and Merck Romania; support for attending meetings and/or travel from Merck Romania and Reckitt; all outside the submitted work. J Ärnlöv reports Payment or honoraria for lectures from AstraZeneca and Boehringer Ingelheim; participation on a Advisory Board with AstraZeneca, Astella, outside the submitted work. O C Baltatu reports support for their participation in the current manuscript from Alfaisal University, Anima Institute (AI) Research Professor Fellowship, and National Council for Scientific and Technological Development Fellowship (CNPq, 304224/2022-7); leadership or fiduciary role in other board, society, committee or advocacy group (paid or unpaid), from VividiWise Analytics, and São José dos Campos Tech Park, outside the submitted work. L Belo reports other financial or non-financial support from FCT in the scope of the project UIDP/04378/2020 and UIDB/04378/2020 of UCIBIO and the project LA/P/0140/2020 of i4HB, outside the submitted work. S Bhaskar reports grants or contacts from Japan Society for the Promotion of Science (JSPS), Japanese Ministry of Education, Culture, Sports, Science and Technology (MEXT) through Grant-in-Aid for Scientific Research (KAKENHI) (23KF0126) and JSPS and the Australian Academy of Science through JSPS International Fellowship (P23712); leadership or fiduciary role in other board, society, committee or advocacy group (paid or unpaid), with the Rotary District 9675, Sydney, Australia, Global Health & Migration Hub Community, Global Health Hub Germany, Berlin, Germany, PLOS One, BMC Neurology, Frontiers in Neurology, Frontiers in Stroke, Frontiers in Public Health, Journal of Aging Research & BMC Medical Research Methodology, College of Reviewers, Canadian Institutes of Health Research (CIHR), Government of Canada, World Headache Society, Bengaluru, India, Cariplo Foundation, Milan, Italy, National Cerebral and Cardiovascular Center, Department of Neurology, Division of Cerebrovascular Medicine and Neurology, Suita, Osaka, Japan, and Cardiff University Biobank, Cardiff, UK; all outside the submitted work. E J Boyko reports Payment or honoraria for lectures, presentations, speakers bureaus, manuscript writing or educational events from Korean Diabetes Association, International Society for the Diabetic Foot, Diabetes Association of the R.O.C. (Taiwan), American Diabetes Association; support for attending meetings and/or travel from Korean Diabetes Association, Diabetes Association of the R.O.C. (Taiwan), International Society for the Diabetic Foot; all outside the submitted work. M Carvalho reports other financial or non-financial support from LAQV/REQUIMTE, University of Porto, Porto, Portugal, and FCT/MCTES under the scope of the project UIDP/50006/2020 (DOI 10.54499/UIDP/50006/2020). N Conrad reports grants or contracts from Research Foundation Flanders (grant number 12ZU922N) through payments to their institute, all outside the submitted work. D Flood reports grants or contracts from NHLBI (award number K23HL161271); consulting fees from serving as a diabetes consultant for the World Health Organization in 2023-2024; all outside the submitted work. A A Fomenkov reports support for their participation in the present manuscript from the Ministry of Science and Higher Educa-tion of the Russian Federation (themes no. 122042600086-7). R C Franklin reports Support for attending meetings and/or travel from ACTM – Annual Conference 2022-2024; leadership or fiduciary role in other board, society, committee or advocacy group (paid or unpaid), from President of Australasian College of Tropical Medicine, outside the submitted work. A Guha reports grants or contracts with the Department of Defense from American Heart Association; leadership or fiduciary role in other board, society, committee or advocacy group (paid or unpaid), with ZERO Cancer health disparities advisory group; outside the submitted work. A Hassan reports Consulting fees from Novartis, Sanofi Genzyme, Biologix, Merck, Hikma Pharma, Janssen, Inspire Pharma, Future Pharma, Elixir pharma; payment or honoraria for lectures, presentations, speakers bureaus, manuscript writing or educational events from Novartis, Allergan, Merck, Biologix, Janssen, Roche, Sanofi Genzyme, Bayer, Hikma Pharma, Al Andalus, Chemipharm, Lundbeck, Inspire Pharma, Future Pharma and Habib Scientific Office, and Everpharma; support for attending meetings and/or travel from Novartis, Allergan, Merck, Biologix, Roche, Sanofi Genzyme, Bayer, Hikma Pharma, Chemipharm, and Al Andalus and Clavita pharm; leadership or fiduciary role in other board, society, committee or advocacy group (paid or unpaid), as Vice president of MENA headache society, Board member of Multiple Sclerosis chapter of the Egyptian Society of Neurology, Board member of headache chapter of the Egyptian Society of Neurology, member of committee of Education of the international Headache Society (IHS), membership committee of IHS, and regional committee of HIS; all outside the submitted work. A M Hopkins reports support for their participation in the manuscript from National Health and Medical Research Council (Australia); grants or contacts from Tour de Cure, The Hospital Research Foundation, and Grant funding from Boehringer Ingelheim, outside the submitted work. C Hu reports support for their participation in the present manuscript from the National Social Science Fund of China (grant number: 24CSH106).I Ilic reports support for their participation in the present manuscript from the Ministry of Education, Science and Technological Development, Republic of Serbia (project No 175042, 2011-2023).M Ilic reports support for their participation in the present manuscript from the Ministry of Technological Development and Innovation of the Republic of Serbia (no. 451-03-47/2023-01/200111). R M Islam is Associate Editor of Climacteric, the official journal of the International Menopause Society (IMS), and a member of the IMS Publication Steering Committee; these roles are unrelated to the content of this submission. N E Ismail reports Leadership or fiduciary role in other board, society, committee or advocacy group, unpaid, Council Member and The Bursar, Malaysian Academy of Pharmacy (MAP), Malaysia, and the Committee Member, Malaysian Pharmacists Society (MPS) Education Chapter Committee, Malaysia, outside the submitted work. J J Jozwiak reports payment or honoraria for lectures, presentations, speakers bureaus, manuscript writing or educational events from NOVARTIS, ADAMED, and AMGEN; outside the submitted work. S K Kamarajah reports support for their participation in the current manuscript from NIHR Doctoral Fellowship scheme (NIHR300175). The funders had no role in study design or writing of this report. The views expressed are those of the authors and not necessarily those of the National Health Service, the NIHR or the UK Department of Health and Social Care. M Kivamaki reports grants or contracts Wellcome Trust, UK (221854/Z/20/Z), National Institute on Aging (NIH), US (R01AG056477), Medical Research Council, UK (MR/R024227/1, MR/Y014154/1), Academy of Finland (350426) through grants to their institutions, outside the submitted work. K Krishan reports other non-financial support from the UGC Centre of Advanced Study, CAS II, awarded to the Department of Anthropology, Panjab University, Chandigarh, India, outside the submitted work. B Lacey reports grants or contracts from Support from UK Biobank, funded largely by the UK Medical Research Council and Wellcome through payments to their institutions, outside the submitted work. M Lee reports support for their participation in the current manuscript from the Ministry of Education of the Republic of Korea and the National Research Foundation of Korea (NRF-2023S1A3A2A05095298). M-C Li reports support for their participation in the current manuscript from National Science and Technology Council, Taiwan (NSTC 113-2314-B-003-002; leadership or fiduciary role in other board, society, committee or advocacy group (paid or unpaid), as Technical Editor, Journal of the American Heart Association, outside the submitted work. D Lindholm reports stock or stock options and other financial interests in AstraZeneca as a former employee, outside the submitted work. S Liu reports grants or contracts from NIH R01ES031391; R01DK125403; R01HL156518; R01HL164485l R01ES034014; U01CA259208; R01HL164485, Royalties or licenses from Uptodate.com; consulting fees from American Society for Nutrition, Fred Hutchinson Cancer Research Center, Guangdong Provincial Hospital, Novo Nordisk, Research Foundation of State University of New York, and Twinhealth.com; support for attending meetings and/or travel from the American Heart Association, Chinese Nutrition Society, European Diabetes and Nutrition Study Group, and American Society of Nutrition; participation on a data safety monitoring board or advisory board with The Select Trial sponsored by Novo Nordisk; all outside the submitted work. S Lorkowski reports grants or contacts from dsm-firmenich (formerly DSM Nutritional Products) through payments to their institution; consulting fees from Danone, Novartis Pharma, and Swedish Orphan Biovitrum (SOBI); payment or honoraria for lectures, presentations, speakers bureaus, manuscript writing or educational events from AMARIN Germany, Amedes Holding, AMGEN, Berlin-Chemie, Boehringer Ingelheim Pharma, Daiichi Sankyo Deutschland, Danone, Hubert Burda Media Holding, Janssen-Cilag, Lilly Deutschland, Novartis Pharma, Novo Nordisk Pharma, Roche Pharma, Sanofi-Aventis, Swedish Orphan Biovitrum (SOBI), and SYNLAB Holding Deutschland; support for attending meetings and/or travel from AMGEN; participation on a data safety monitoring board or advisory board with AMGEN, Daiichi Sankyo Deutschland, Novartis Pharma, and Sanofi-Aventis; all outside the submitted work. E Lytvyak reports grants or contracts from College of Physicians and Surgeons of Alberta, Government of Alberta, and Advanz Pharma; other financial or non-financial interests in University of Alberta and Alberta Health Services; all outside the submitted work. H R Marateb reports grants or contracts from The Beatriu de Pinós post-doctoral programme from Agency for Management of University and Research Grants, Government of Catalonia program (#2020 BP 00261) through payments to their institution, and from Agency for Management of University and Re-search Grants, Knowledge Industry Grants for 2024. Modality A. LLAVOR (2024 LLAV 00083), outside the submitted work. S A Meo reports grants or contracts from Researchers Supporting Project, King Saud University, Riyadh, Saudi Arabia (RSP-2025 R47), outside the submitted work. S Nomura reports support for their participation in the current manuscript from Ministry of Education, Culture, Sports, Science and Technology of Japan (24H00663), and Precursory Research for Embryonic Science and Technology from the Japan Science and Technology Agency (JPMJPR22R8). B Oancea reports grants or contracts from the MRID, project PNRR-I8 no 842027778., contract no 760096, outside the submitted work. A Ortiz reports grants or contracts Sanofi and through the Catedra Mundipharma-UAM of diabetic kidney disease and the Catedra AstraZeneca-UAM of chronic kidney disease and electrolytes; consulting fees, speaker fees or travel support from Advicciene, Astellas, AstraZeneca, Amicus, Amgen, Fresenius Medical Care, GSK, Boehringer-Ingelheim, Bayer, Sanofi-Genzyme, Menarini, Kyowa Kirin, Alexion, Idorsia, Chiesi, Otsuka, Novo-Nordisk and Vifor Fresenius Medical Care Renal Pharma; leadership or fiduciary role in other board, society, committee or advocacy group, unpaid, with council ERA. SOMANE; all outside the submitted work. S K Panda reports support for their participation in the current manuscript from Siksha ‘O’ Anusandhan (Deemed to be University) through their salary; grants or contracts from DST-GOVT. OF ODISHA (Letter No. 3444/ST); leadership or fiduciary role in other board, society, committee or advocacy group (paid or unpaid) as associate editor at *Heliyon*; outside the submitted work. R Passera reports Participation on a data safety monitoring board or advisory board as Member of the Data Safety Monitoring Board of the clinical trial “Consolidation with ADCT-402 (loncastuximab tesirine) after immunochemotherapy: a phase II study in BTKi-treated/ineligible Relapse/Refractory Mantle Cell Lymphoma (MCL) patients” - FIL, Fondazione Italiana Linfomi, Alessandria; leadership or fiduciary role in other board, society, committee or advocacy group (paid or unpaid), as Member of the EBMT Statistical Committee, European Society for Blood and Marrow Transplantation, Paris (F), and as a past member 2020–2023 (biostatistician) of the IRB/IEC Comitato Etico AO SS. Antonio e Biagio Alessandria-ASL AL-VC; all outside the submitted work. S Rege reports Leadership or fiduciary role in other board, society, committee or advocacy group (paid or unpaid), as Operational Lead – International Society for Pharmacoeconomics and Outcomes Research (ISPOR) Medication Adherence and Persistence (MAP) Special Interest Group (SIG), Review Editor – Editorial Board of Pharmacoepidemiology section within Frontiers in Pharmacology, and Academic Editor – PLOS ONE Editorial Board; outside the submitted work. Y Samodra reports leadership or fiduciary role in other board, society, committee or advocacy group (paid or unpaid), with the Benang Merah Research Center, Indonesia as co-founder; outside the submitted work. A E Schutte reports grants or contracts from National Health and Medical Research Council of Australia and Medical Research Future Fund; consulting fees from Sky Labs, Servier, and Medtronic; payment or honoraria for lectures, presentations, speakers bureaus, manuscript writing or educational events from Servier, Abbott, Sanofi, AstraZeneca, Medtronic, Omron, Aktiia; support for attending meetings and/or travel from Servier, and Medtronic; leadership or fiduciary role in other board, society, com-mittee or advo-cacy group (paid or unpaid), as Co-Chair, National Hypertension Taskforce of Australia, Secretary, Australian Cardiovascular Alliance, and Board Member, Hypertension Australia; all outside the submitted work. V Sharma reports other financial or non-financial support from DFSS (MHA)'s research project (DFSS28(1)2019/EMR/6) at Institute of Forensic Science & Criminology, Panjab University, Chandigarh, India, outside the submitted work. J A Singh reports consulting fees from ROMTech, Atheneum, Clearview healthcare partners, American College of Rheumatology, Yale, Hulio, Horizon Pharmaceuticals, DINORA, ANI/Exeltis, USA, Frictionless Solutions, Schipher, Crealta/Horizon, Medisys, Fidia, PK Med, Two labs, Adept Field Solutions, Clinical Care options, Putnam associates, Focus forward, Navigant consulting, Spherix, MedIQ, Jupiter Life Science, UBM LLC, Trio Health, Medscape, WebMD, and Practice Point communications; and the National Institutes of Health]; payment of honoraria for lectures, presentations, speakers bureaus, manuscript writing or education events as a member of the speaker's bureau of Simply Speaking; support for attending meetings as a past steering committee member of OMERACT; participation on a data safety monitoring board or advisory board with the FDA Arthritis Advisory Committee; leadership or fiduciary role in other board, society, committee or advocacy group, paid as a past steering committee member of the OMERACT (an international organization that develops measures for clinical trials and receives arm's length funding from 12 pharmaceutical companies), unpaid as a Co-Chair of the Veterans Affairs Rheumatology Field Advisory Committee, and unpaid as an editor and Director of the UAB Cochrane Musculoskeletal Group Satellite Center on Network Meta-analysis; stock of stock options in Atai life sciences, Kintara therapeutics, Intelligent Biosolutions, Acumen pharmaceutical, TPT Global Tech, Vaxart pharmaceuticals, Atyu biopharma, Adaptimmune Therapeutics, GeoVax Labs, Pieris Pharmaceuticals, Enzolytics, Seres Therapeutics, Tonix Pharmaceuticals Holding Corp, and Charlotte's Web Holdings, and previous stock options in Amarin, Viking, and Moderna Pharmaceuticals; outside the submitted work. R Tabares-Seisdedos reports grants or contracts from Valencian Regional Government's Ministry of Education (PROMETEO/CIPROM/2022/58 and the Spanish Ministry of Science, Innovation and Universities (PID2021-129099OB-I00); outside the submitted work. J H V Ticoalu reports Leadership or fiduciary role in other board, society, committee or advocacy group (paid or unpaid), with Benang Merah Research Center, Indonesia as co-founder, outside the submitted work. M V Titova reports support for their participation in the current manuscript from the Ministry of Science and Higher Education of the Russian Federation (themes no. 122042600086-7). D Trico reports Payment or honoraria for lectures, presentations, speakers bureaus, manuscript writing or educational events from AstraZeneca, Eli Lilly, and Novo Nordisk; support for attending meetings and/or travel from AstraZeneca; participation on a data safety monitoring board or advisory board from Amarin, Boehringer Ingelheim, and Novo Nordisk; leadership or fiduciary role in other board, society, committee or advocacy group (paid or unpaid), with EASD Early Career Academy and EASD Committee on Clinical Affairs; receipt of equipment, materials, drugs, medical writing, gifts or other services from Abbott and PharmaNutra; all outside the submitted work. E Upadhyay reports patents issued, planned, or pending, for A system and method of reusable filters for anti-pollution mask, A system and method for electricity generation through crop stubble by using microbial fuel cells, A system for disposed personal protection equipment (PPE) into biofuel through pyrolysis and method, A novel herbal pharmaceutical aid for formulation of gel and method thereof, Herbal drug formulation for treating lung tissue degenerated by particulate matter exposure, and A method to transform cow dung into the wall paint by using natural materials and composition thereof; leadership or fiduciary role in other board, society, committee or advocacy group (paid or unpaid), with the Executive Council Member, Indian Meteorological Society, Jaipur Chapter. (India) and Member Secretary-DSTPURSE Program; all outside the submitted work. P Willeit reports grants or contracts from Novartis Pharmaceuticals, outside the submitted work. M Zielińska reports other financial or non-financial in Alexion, AstraZeneca Rare Disease as an employee. E Zweck reports Payment or honoraria for lectures, presentations, speakers bureaus, manuscript writing or educational events and support for travel and/or travel from Abiomed, outside the submitted work.
