
JOURNAL INFORMATION
==============================
NLM Title Abbreviation: Lancet
Journal ID: Lancet
NLM Journal ID: 2985213R

Lancet (London, England)

ISSN: 0140-6736
EISSN: 1474-547X

ARTICLE INFORMATION
==============================
PMCID: PMC12695672
PMID: 40849136
DOI: 10.1016/S0140-6736(25)01722-2
Article ID: S0140-6736(25)01722-2
Article version: 1
Subjects: Department of Error

Department of Error

Print publication date: 2025 Aug 23
Electronic publication date: 2025 Aug 21
Volume: 406
Issue: 10505
First page: 810
Last page: 810
Copyright: © 2025 The Author(s). Published by Elsevier Ltd.
Copyright year: 2025
Copyright holder: Elsevier Ltd
License: This is an open access article under the CC BY license (http://creativecommons.org/licenses/by/4.0/).
License URL: https://creativecommons.org/licenses/by/4.0/

Erratum for: Global, regional, and national prevalence of adult overweight and obesity, 1990–2021, with forecasts to 2050: a forecasting study for the Global Burden of Disease Study 2021 405 10481 8 3 2025 813 838 Lancet (London, England) 10.1016/S0140-6736(25)00355-1 PMC11920007 40049186

==============================
GBD 2021 Adult BMI Collaborators. Global, regional, and national prevalence of adult overweight and obesity, 1990–2021, with forecasts to 2050: a forecasting study for the Global Burden of Disease Study 2021. Lancet 2025; 405: 813–38—For this Article, Nayu Ikeda should have been included in the list of GBD 2021 Adult BMI Collaborators. This correction has been made to the online version as of Aug 21, 2025.

