# Supplementary material for: Global, regional, and national prevalence of adult overweight and obesity, 1990–2021, with forecasts to 2050: a forecasting study for the Global Burden of Disease Study 2021
Source: Lancet. 2025 Mar 8;405(10481):813–38. doi: 10.1016/S0140-6736(25)00355-1 (PMC11920007; doi:10.1016/S0140-6736(25)00355-1)
Supplement: Supplementary appendix 2 [file mmc2.pdf]

THE LANCET

Supplementary appendix 2

This appendix formed part of the original submission and has been peer reviewed. We post it as supplied by the authors.

Supplement to: GBD 2021 Adult BMI Collaborators. Global, regional, and national prevalence of adult overweight and obesity, 1990–2021, with forecasts to 2050: a forecasting study for the Global Burden of Disease Study 2021. *Lancet* 2025; published online March 3. https://doi.org/10.1016/S0140-6736(25)00355-1.

Appendix 2: Authorship appendix to "Global, regional, and national prevalence of adult overweight and obesity, 1990–2021, with forecasts to 2050: a forecasting study for the Global Burden of Disease Study 2021"

This appendix provides further authorship detail for "Global, regional, and national prevalence of adult overweight and obesity, 1990–2021, with forecasts to 2050: a forecasting study for the Global Burden of Disease Study 2021"

Table of Contents

GBD 2021 Global Adult Obesity Collaborators ... 2

Affiliations ... 6

Authors' Contributions ... 34

- Managing the overall research enterprise ... 34
- Writing the first draft of the manuscript ... 34
- Primary responsibility for applying analytical methods to produce estimates ... 34
- Primary responsibility for seeking, cataloguing, extracting, or cleaning data; designing or coding figures and tables ... 34
- Providing data or critical feedback on data sources ... 34
- Developing methods or computational machinery ... 36
- Providing critical feedback on methods or results ... 36
- Drafting the work or revising it critically for important intellectual content ... 40
- Managing the estimation or publication process ... 44

GBD 2021 Global Adult Obesity Collaborators

Marie Ng*, Emmanuela Gakidou*, Justin Lo*, Yohannes Habtegiorgis Abate, Cristiana Abbafati, Nasir Abbas, Mohammadreza Abbasian, Samar Abd ElHafeez, Wael M Abdel-Rahman, Sherief Abd-Elsalam, Arash Abdollahi, Meriem Abdoun, Deldar Morad Abdulah, Rizwan Suliankatchi Abdulkader, Auwal Abdullahi, Armita Abedi, Hansani Madushika Abeywickrama, Alemwork Abie, Richard Gyan Aboagye, Shady Abohashem, Dariush Abtahi, Hasan Abualruz, Bilyaminu Abubakar, Rana Kamal Abu Farha, Hana J Abukhadijah, Niveen ME Abu-Rmeileh, Salahdein Aburuz, Ahmed Abu-Zaid, Lisa C Adams, Mesafint Molla Adane, Isaac Yeboah Addo, Kamoru Ademola Adedokun, Nurudeen A Adegoke, Abiola Victor Victor Adepoju, Ridwan Olamilekan Adesola, Temitayo Esther Adeyeoluwa, Usha Adiga, Qorinah Estiningtyas Sakilah Adnani, Siamak Afaghi, Saira Afzal, Muhammad Sohail Afzal, Thilini Chanchala Agampodi, Shahin Aghamiri, César Agostinis Sobrinho, Williams Agyemang-Duah, Austin J Ahlstrom, Danish Ahmad, Sajjad Ahmad, Aqeel Ahmad, Muayyad M Ahmad, Fuzail Ahmad, Noah Ahmad, Haroon Ahmed, Muktar Beshir Ahmed, Ayman Ahmed, Meqdad Saleh Ahmed, Mehrunnisha Sharif Ahmed, Syed Anees Ahmed, Marjan Ajami, Samina Akhtar, Mohammed Ahmed Akkaif, Ashley E Akrami, Tariq A Alalwan, Ziyad Al-Aly, Khurshid Alam, Rasmieh Mustafa Al-amer, Amani Alansari, Fahmi Y Al-Ashwal, Mohammed Albashtawy, Wafa A Aldhaleei, Bezawit Abeje Alemayehu, Abdelazeem M Algammal, Khalid F Alhabib, Hanadi Al Hamad, Syed Mahfuz Al Hasan, Dari Alhuwail, Rafat Ali, Abid Ali, Waad Ali, Mohammed Usman Ali, Sheikh Mohammad Alif, Samah W Al-Jabi, Syed Mohamed Aljunid, Ahmad Alkhatib, Sabah Al-Marwani, Mahmoud A Alomari, Saleh A Alqahtani, Rajaa M Mohammad Al-Raddadi, Ahmad Alrawashdeh, Intima Alrimawi, Sahel Majed Alrousan, Najim Z Alshahrani, Omar Al Ta'ani, Zain Al Ta'ani, Zaid Altaany, Awais Altaf, Yazan Al Thaher, Nelson Alvis-Guzman, Mohammad Al-Wardat, Yaser Mohammed Al-Worafi, Safwat Aly, Hany Aly, Hosam Alzahrani, Abdallah Alzoubi, Karem H Alzoubi, Md. Akib Al-Zubayer, Sohrab Amiri, Hubert Amu, Dickson A Amugsi, Ganiyu Adeniyi Amusa, Roshan A Ananda, Robert Ancuceanu, Catalina Liliana Andrei, Ranjit Mohan Anjana, Sumbul Ansari, Mohammed Tahir Ansari, Catherine M Antony, Iyadunni Adesola Anuoluwa, Boluwatife Stephen Anuoluwa, Saeid Anvari, Saleha Anwar, Anayochukwu Edward Anyasodor, Geminn Louis Carace Apostol, Juan Pablo Arab, Jalal Arabloo, Mosab Arafat, Aleksandr Y Aravkin, Demelash Areda, Hidayat Arifin, Mesay Arkew, Benedetta Armocida, Johan Ärnlöv, Mahwish Arooj, Anton A Artamonov, Kurnia Dwi Artanti, Ashokan Arumugam, Mohammad Asghari-Jafarabadi, Tahira Ashraf, Bernard Kwadwo Yeboah Asiamah-Asare, Anemaw A Asrat, Thomas Astell-Burt, Seyyed Shamsadin Athari, Prince Atorkey, Alok Atreya, Zaure Maratovna Aumoldaeva, Hamzeh Awad, Mamaru Ayenew Awoke, Adedapo Wasiu Awotidebe, Setognal Birara Aychiluhm, Ali Azargoonjahromi, Amirali Azimi, Sadat Abdulla Aziz, Shahkaar Aziz, Ahmed Y. Azzam, Domenico Azzolino, Peter S Azzopardi, Mina Babashahi, Giridhara Rathnaiah Babu, Ashish D Badiye, Nasser Bagheri, Yogesh Bahurupi, Ruhai Bai, Atif Amin Baig, Shankar M Bakkannavar, Senthilkumar Balakrishnan, Ovidiu Constantin Baltatu, Kiran Bam, Maciej Banach, Rajon Banik, Mainak Bardhan, Hiba Jawdat Barqawi, Simon Barquera, Lingkan Barua, Zarrin Basharat, Shahid Bashir, Mohammad-Mahdi Bastan, Saurav Basu, Reza Bayat, Mulat Tirfie Bayih, Narasimha M Beeraka, Tahmina Begum, Umar Muhammad Bello, Abdulrahman Babatunde Bello, Luis Belo, Isabela M Bensenor, Maria Bergami, Kidanemaryam Berhe, Abiye Assefa Berihun, Ajeet Singh Bhadoria, Akshaya Srikanth Bhagavathula, Neeraj Bhala, Jaideep Singh Bhalla, Ravi Bharadwaj, Pankaj Bhardwaj, Nikha Bhardwaj, Sonu Bhaskar, Ajay Nagesh Bhat, Priyadarshini Bhattacharjee, Shuvarthi Bhattacharjee, Jasvinder Singh Bhatti, Gurjit Kaur Bhatti, Andras Bikov, Cem Bilgin, Catherine Bisignano, Bijit Biswas, Bruno Bizzozero Peroni, Espen Bjertness, Tone Bjørge, Srinivasa Rao Bolla, Hamed Borhany, Samuel Adolf Bosoka, Souad Bouaoud, Edward J Boyko, Dejana Braithwaite, Javier Brazo-Sayavera, Hermann Brenner, Gabrielle

Britton, Dana Bryazka, Raffaele Bugiardini, Linh Phuong Bui, Felix Busch, Yasser Bustanji, Nadeem Shafique Butt, Zahid A Butt, Daniela Calina, Luciana Aparecida Campos, Ismael Campos-Nonato, Si Cao, Yin Cao, Angelo Capodici, Andre F Carvalho, Márcia Carvalho, Alberico L Catapano, Monica Cattafesta, Maria Sofia Cattaruzza, Luca Cegolon, Francieli Cembranel, Edina Cenko, Ester Cerin, Achille Cernigliaro, Joshua Chadwick, Chiranjib Chakraborty, Raymond N C Chan, Jung-Chen Chang, Vijay Kumar Chattu, Anis Ahmad Chaudhary, Akhilanand Chaurasia, Guangjin Chen, An-Tian Chen, Haowei Chen, Esther T W Cheng, Nicholas WS Chew, Gerald Chi, Ritesh Chimoriya, Patrick R Ching, Dong-Woo Choi, Bryan Chong, Hitesh Chopra, Shivani Chopra, Hou In Chou, Sonali Gajanan Choudhari, Dinh-Toi Chu, Sunghyun Chung, Sheng-Chia Chung, Muhammad Chutiyami, Karly I Cini, Iolanda Cioffi, Rebecca M Cogen, Daniel Collado-Mateo, Alyssa Columbus, Nathalie Conrad, Michael H Criqui, Natalia Cruz-Martins, Steven Cummins, Emanuele D'Amico, Lucio D'Anna, Mario D'Oria, Omid Dadras, Xiaochen Dai, Mayank Dalakoti, Rakhi Dandona, Lalit Dandona, Pojsakorn Danpanichkul, Samuel Demissie Darcho, Reza Darvishi Cheshmeh Soltani, Alanna Gomes da Silva, Kairat Davletov, Ivan Delgado-Enciso, Edgar Denova-Gutiérrez, Meseret Derbew Molla, Ismail Dergaa, Aragaw Tesfaw Desale, Vinoth Gnana Chellaiyan Devanbu, Devananda Devegowda, Syed Masudur Rahman Dewan, Arkadeep Dhali, Samath Dhamminda Dharmaratne, Meghnath Dhimal, Bibha Dhungel, Daniel Diaz, Monica Dinu, Milad Dodangeh, Sushil Dohare, Klara Georgieva Dokova, Neda Dolatkhah, Camila Bruneli do Prado, Fariba Dorostkar, Ojas Prakashbhai Doshi, Rajkumar Prakashbhai Doshi, Robert Kokou Dowou, Viola Savy Dsouza, Mi Du, Samuel C Dumith, Dorothea Dumuid, Bruce B Duncan, Sulagna Dutta, Arkadiusz Marian Dziedzic, Alireza Ebrahimi, Behrad Eftekhari, Ashkan Eighaei Sedeh, Michael Ekholuenetale, Mohamed Ahmed Eladl, Rabie Adel El Arab, Said El-Ashker, Iffat Elbarazi, Ibrahim Farahat El Bayoumy, Islam Y Elgendy, Muhammed Elhadi, Waseem El-Huneidi, Ashraf A El-Metwally, Mohamed A Elmonem, Mohamed Hassan Elnaem, Randa Elsheikh, Ibrahim Elsohaby, Chadi Eltaha, Theophilus I Emeto, Maysa Eslami, Ugochukwu Anthony Eze, Heidar Fadavian, Adeniyi Francis Fagbamigbe, Ildar Ravisovich Fakhradiyev, Seyed Nooreddin Faraji, Carla Sofia e Sá Farinha, MoezAlIslam Ezzat Mahmoud Faris, Umar Farooque, Hossein Farrokhpour, Samuel Aanuoluwapo Fasusi, Patrick Fazeli, Timur Fazylov, Alireza Feizkhah, Ginenus Fekadu, Xiaoqi Feng, João C Fernandes, Rodrigo Fernandez-Jimenez, Nuno Ferreira, Bikila Regassa Feyisa, Florian Fischer, David Flood, Nataliya A Foigt, Morenike Oluwatoyin Folayan, Artem Alekseevich Fomenkov, Roham Foroumadi, Celia Fortuna Rodrigues, Matteo Foschi, Maryam Fotouhi, Kate Louise Francis, Richard Charles Franklin, Aleš Gába, Muktar A Gadanya, Abhay Motiramji Gaidhane, Yaseen Galali, Silvano Gallus, Balasankar Ganesan, Shivaprakash Gangachannaiah, Wendy Paola Gastélum Espinoza, Miglas Welay Gebregergis, Teferi Gebru Gebremeskel, Lemma Getacher, Fataneh Ghadirian, Amir Ghaffari Jolfayi, Seyyed-Hadi Ghamari, Ramy Mohamed Ghazy, Artyom Urievich Gil, Tiffany K Gill, Elena V Gnedovskaya, Mahaveer Golechha, Davide Golinelli, Michal Grivna, Ashna Grover, Zhongyang Guan, Shi-Yang Guan, Giovanni Guarducci, Mohammed Ibrahim Mohialdeen Gubari, Avirup Guha, Damitha Asanga Gunawardane, Zheng Guo, Rajeev Gupta, Anish Kumar Gupta, Rahul Gupta, Sapna Gupta, Vivek Kumar Gupta, Roberth Steven Gutiérrez-Murillo, Jose Guzman-Esquivel, Najah R Hadi, Zahra Hadian, Nadia M Hamdy, Sajid Hameed, Samer Hamidi, Mohammad Hamiduzzaman, Asif Hanif, Nasrin Hanifi, Graeme J Hankey, Allie Haq, Netanja I Harlianto, Josep Maria Haro, Risky Kusuma Hartono, Faizul Hasan, Mohammad Hashem Hashempur, Md Saquib Hasnain, Amr Hassan, Nageeb Hassan, Soheil Hassanipour, Afagh Hassanzade Rad, Rasmus J Havmoeller, Simon I Hay, Wen-Qiang He, Jeffrey J Hebert, Golnaz Heidari, Mehdi Hemmati, Yuta Hiraike, Nguyen Quoc Hoan, Mai Hoang, Ramesh Holla, Praveen Hoogar, Ashley Mark Hopkins, Alamgir Hossain, Hassan Hosseinzadeh, Sorin Hostiuc, Mihaela Hostiuc, Zin Wai Htay, Chengxi Hu, Junjie Huang, Tsegaye Gebreyes Hundie, Mohamed Ibrahim Husseiny, Hong-Han

Huynh, Ivo Iavicoli, Anel Ibrayeva, Olayinka Stephen Ilesanmi, Irena M Ilic, Milena D Ilic, Mohammad Tarique Imam, Leeberk Raja Inbaraj, Arit Inok, Lalu Muhammad Irham, Md. Rabiul Islam, Sheikh Mohammed Shariful Islam, Rakibul M Islam, Nahlah Elkudssiah Ismail, Hiroyasu Iso, Gaetano Isola, Mosimah Charles Ituka, Masao Iwagami, Chinwe Juliana Iwu-Jaja, Assefa N Iyasu, Vinothini J, Louis Jacob, Shabbar Jaffar, Haitham Jahrami, Akhil Jain, Ammar Abdulrahman Jairoun, Mihajlo Jakovljevic, Mohamed Lamrana Jalloh, Syed Sarmad Javaid, Sathish Kumar Jayapal, Umesh Jayarajah, Shubha Jayaram, Rime Jebai, Felix K Jebasingh, Alelign Tasew Jema, Mohammad Jokar, Jost B Jonas, Jobinse Jose, Nitin Joseph, Charity Ehimwenma Joshua, Jacek Jerzy Jozwiak, Mikk Jürisson, Billingsley Kaambwa, Ali Kabir, Zubair Kabir, Ashish Kumar Kakkar, Sanjay Kalra, Sivesh Kathir Kamarajah, Saddam Fuad Kanaan, Samuel Berchi Kankam, Kehinde Kazeem Kanmodi, Neeti Kapoor, Mehrdad Karajizadeh, Paschalis Karakasis, Reema A Karasneh, Yeganeh Karimi, Arman Karimi Behnagh, Nicholas J Kassebaum, Joonas H Kauppila, Gbenga A Kayode, Dimitrios Kehagias, Jessica A Kerr, Ariz Keshwani, Emmanuelle Kesse-Guyot, Mohammad Keykhaei, Inn Kynn Khaing, Himanshu Khajuria, Pantea Khalili, Alireza Khalilian, Mohamed Khalis, Mohammad Jobair Khan, Maseer Khan, Nusrat Khan, Md Abdullah Saeed Khan, Ajmal Khan, Moien AB Khan, Shaghayegh Khanmohammadi, Khaled Khatab, Moawiah Mohammad Khatatbeh, Maryam Khayamzadeh, Feriha Fatima Khidri, Fatemeh Khorashadizadeh, Atulya Aman Khosla, Sepehr Khosravi, Mahmood Khosrowjerdi, Jagdish Khubchandani, Helda Khusun, Jinho Kim, Kwanghyun Kim, Min Seo Kim, Yun Jin Kim, Ruth W Kimokoti, Adnan Kisa, Ladli Kishore, Mika Kivimäki, Michail Kokkorakis, Ali-Asghar Kolahi, Farzad Kompani, Oleksii Korzh, Karel Kostev, Sindhura Lakshmi Koulmane Laxminarayana, Irene Akwo Kretchy, Kewal Krishan, Chong-Han Kua, Barthelemy Kuate Defo, Mukhtar Kulimbet, Vishnutheertha Kulkarni, Ashish Kumar, Vijay Kumar, G Anil Kumar, Satyajit Kundu, Setor K Kunutsor, Om P Kurmi, Maria Dyah Kurniasari, Dian Kusuma, Ville Kytö, Ben Lacey, Chandrakant Lahariya, Daphne Teck Ching Lai, Hanpeng Lai, Iván Landires, Bagher Larijani, Kamaluddin Latief, Carlo La Vecchia, Nhi Huu Hanh Le, Munjae Lee, Sang-woong Lee, Wei-Chen Lee, Seung Won Lee, Paul H Lee, Ming-Chieh Li, Yongze Li, Weilong Li, Stephen S Lim, Queran Lin, Jialing Lin, Daniel Lindholm, Paulina A Lindstedt, Simin Liu, Erand Llanaj, José Francisco López-Gil, Stefan Lorkowski, Giancarlo Lucchetti, Alessandra Lugo, Angelina M Lutambi, Lei Lv, Ellina Lytvyak, Zheng Feei Ma, Monika Machoy, Javier A Magaña Gómez, Nastaran Maghbouli, Mehrdad Mahalleh, Nozad H Mahmood, Elham Mahmoudi, Rituparna Maiti, Konstantinos Christos C Makris, Kashish Malhotra, Ahmad Azam Malik, Iram Malik, Deborah Carvalho Malta, Abdullah A Mamun, Marjan Mansourian, Emmanuel Manu, Hamid Reza Marateb, Mirko Marino, Abdoljalal Marjani, Ramon Martinez-Piedra, Santi Martini, Miquel Martorell, Sammer Marzouk, Stefano Masi, Soroush Masrouri, Yasith Mathangasinghe, Manu Raj Mathur, Fernanda Penido Matozinhos, Thushara Matthias, Rita Mattiello, Mohsen Mazidi, Steven M McPhail, Enkeleint A Mechili, Riffat Mehboob, Asim Mehmood, Man Mohan Mehndiratta, Kamran Mehrabani-Zeinabad, Tesfahun Mekene Meto, Hadush Negash Meles, Walter Mendoza, Ritesh G Menezes, Emiru Ayalew Mengistie, Sultan Ayoub Meo, Tomislav Mestrovic, Sachith Mettananda, Chamila Dinushi Kukulege Mettananda, Ana Carolina Micheletti Gomide Nogueira de Sá, Ted R Miller, GK Mini, Erkin M Mirrakhimov, Awoke Misganaw, Madhukar Mittal, Ahmed Ismail Mohamed, Mona Gamal Mohamed, Nouh Saad Mohamed, Jama Mohamed, Taj Mohammad, Sakineh Mohammad-Alizadeh-Charandabi, Ibrahim Mohammadzadeh, Shafiu Mohammed, Mustapha Mohammed, Ali H Mokdad, Stefania Mondello, Mohammad Ali Moni, Maryam Moradi, Shane Douglas Morrison, Elias Mossialos, Rohith Motappa, Francesk Mulita, Erin C Mullany, Yanjinlkham Munkhsaikhan, Efren Murillo-Zamora, Sani Musa, Ghulam Mustafa, Sathish Muthu, Woojae Myung, Pirouz Naghavi, Mohsen Naghavi, Ganesh R Naik, Hiten Naik, Gopal Nambi, Vinay Nangia, Jobert Richie Nansseu, Gustavo G Nascimento, Mahmoud

Nassar, Zuhair S Natto, Javaid Nauman, Zakira Naureen, Samidi Nirasha Kumari Navaratna, Biswa Prakash Nayak, Md Fahad Shahariar Nayon, Athare Nazri-Panjaki, Masoud Negahdary, Ruxandra Irina Negoi, Ionut Negoi, Seyed Aria Nejadghaderi, Soroush Nematollahi, Samata Nepal, Henok Biresaw Netsere, Josephine W Ngunjiri, Cuong Tat Nguyen, Dang Nguyen, Trang Nguyen, Duc Hoang Nguyen, Phuong The Nguyen, Robina Khan Niazi, Luciano Nieddu, Mahdieh Niknam, Ali Nikoobar, Jan Rene Nkeck, Shuhei Nomura, Syed Toukir Ahmed Noor, Mamoona Noreen, Masoud Noroozi, Nawsherwan, Jean Jacques Noubiap, Mehran Nouri, Chisom Adaobi Nri-Ezedi, Fred Nugen, Dieta Nurrika, Ogochukwu Janet Nzoputam, Erin M O'Connell, Bogdan Oancea, James Odhiambo Oguta, In-Hwan Oh, Hassan Okati-Aliabad, Akinkunmi Paul Okekunle, Osaretin Christabel Okonji, Andrew T Olagunju, Oladotun Victor Olalusi, Timothy Olusegun Olanrewaju, Omotola O Olasupo, Gláucia Maria Moraes Oliveira, Arão Belitardo Oliveira, Yinka Doris Oluwafemi, Hany A Omar, Ahmed Omar Bali, Marcel Opitz, Michal Ordak, Alberto Ortiz, Augustus Osborne, Wael M S Osman, Alaa A M Osman, Uchechukwu Levi Osuagwu, Adrian Otoiu, Abdu Oumer, Amel Ouyahia, Mayowa O Owolabi, Irene Amoakoh Owusu, Kolapo Oyebola, Mahesh Padukudru P A, Alicia Padron-Monedero, Jagadish Rao Padubidri, Sujogya Kumar Panda, Songhomitra Panda-Jonas, Anamika Pandey, Seithikurippu R Pandi-Perumal, Shahina Pardhan, Utsav Parekh, Pragyan Paramita Parija, Romil R Parikh, Eun-Cheol Park, Ava Pashaei, Roberto Passera, Hemal M Patel, Aslam Ramjan Pathan, Dimitrios Patoulias, George C Patton, Susan Paudel, Hamidreza Pazoki Toroudi, Umberto Pensato, Prince Peprah, Gavin Pereira, Marcos Pereira, Arokiasamy Perianayagam, Norberto Perico, Simone Perna, Ionela-Roxana Petcu, Fanny Emily Petermann-Rocha, Hoang Nhat Pham, Roman V Polibin, Djordje S Popovic, Farzad Pourghazi, Akram Pourshams, Jalandhar Pradhan, Pranil Man Singh Pradhan, Manya Prasad, Akila Prashant, Elton Junio Sady Prates, I Gusti Ngurah Edi Putra, Jagadeesh Puvvula, Ibrahim Qattea, Jia-Yong Qiu, Venkatraman Radhakrishnan, Maja R Radojčić, Catalina Raggi, Muhammad Aziz Rahman, Fryad Majeed Rahman, Mohammad Hifz Ur Rahman, Mosiur Rahman, Saeed Rahmani, Vahid Rahmanian, Setyaningrum Rahmawaty, Rajesh Kumar Rai, Ivano Raimondo, Jeffrey Pradeep Raj, Prashant Rajput, Mahmoud Mohammed Ramadan, Chitra Ramasamy, Shakthi Kumaran Ramasamy, Sheena Ramazanu, Kritika Rana, Chhabi Lal Ranabhat, Mithun Rao, Sowmya J Rao, Sina Rashedi, Mohammad-Mahdi Rashidi, Ashkan Rasouli-Saravani, Devarajan Rathish, Santosh Kumar Rauniyar, Ilari Rautalin, David Laith Rawaf, Salman Rawaf, Elrashdy M. Moustafa Mohamed Redwan, Sanika Rege, Ana Reis-Mendes, Giuseppe Remuzzi, Nazila Rezaei, Mohsen Rezaeian, Hossein Rezazadeh, Taeho Gregory Rhee, João Rocha Rocha-Gomes, Mónica Rodrigues, Thales Philipe Rodrigues da Silva, Jefferson Antonio Buendia Rodriguez, Leonardo Roever, Peter Rohloff, Debby Syahru Romadlon, Moustaq Karim Khan Rony, Gholamreza Roshandel, Himanshu Sekhar Rout, Nitai Roy, Godfrey M Rwegerera, Aly M A Saad, Maha Mohamed Saber-Ayad, Cameron John Sabet, Kabir P Sadarangani, Basema Ahmad Saddik, Masoumeh Sadeghi, Mohammad Reza Saeb, Umar Saeed, Sahar Saeedi Moghaddam, Sher Zaman Safi, Amene Saghazadeh, Dominic Sagoe, Amirhossein Sahebkar, Fatemeh Saheb Sharif-Askari, Soumya Swaroop Sahoo, Mirza Rizwan Sajid, Luciane B Salaroli, Mohamed A Saleh, Marwa Rashad Salem, Sohrab Salimi, Yoseph Leonardo Samodra, Vijaya Paul Samuel, Abdallah M Samy, Prasanna K Santhekadur, Milena M Santric-Milicevic, Muhammad Arif Nadeem Saqib, Ushasi Saraswati, Aswini Saravanan, Dianis Wulan Sari, Tanmay Sarkar, Mohammad Sarmadi, Sachin C Sarode, Gargi Sachin Sarode, Michele Sassano, Brijesh Sathian, Ganesh Kumar Saya, Christophe Schinckus, Maria Inês Schmidt, Art Schuermans, Aletta Elisabeth Schutte, Sneha Annie Sebastian, Siddharthan Selvaraj, Mohammad H Semreen, Ashenafi Kibret Sendekie, Pallav Sengupta, Yigit Can Senol, Subramanian Senthilkumaran, Sadaf G Sepanlou, Yashendra Sethi, Allen Seylani, Mahan Shafie, Sweni Shah, Syed Mahboob Shah, Samiah Shahid, Fatemeh Shahrahmani, Moyad Jamal Shahwan, Sunder Sham,

Muhammad Aaqib Shamim, Mehran Shams-Beyranvand, Anas Shamsi, Alfiya Shamsutdinova, Dan Shan, Mohd Shanawaz, Mohammed Shannawaz, Medha Sharath, Sadaf Sharfaei, Amin Sharifan, Anupam Sharma, Ujjawal Sharma, Manoj Sharma, Vishal Sharma, Fateme Sheida, Ali Sheikhy, Rekha Raghuveer Shenoy, Pavanchand H Shetty, Kenji Shibuya, Desalegn Shiferaw, Min-Jeong Shin, Rahman Shiri, Aminu Shittu, Sina Shool, Seyed Afshin Shorofi, Rajan Shrestha, Kerem Shuval, Yafei Si, Nicole R S Sibuyi, Emmanuel Edwar Siddig, Ahmed Kamal Siddiqi, Mithun Sikdar, Diego Augusto Santos Silva, Luís Manuel Lopes Rodrigues Silva, Surjit Singh, Jasvinder A Singh, Amit Singh, Harmanjit Singh, Baljinder Singh, Kalpana Singh, Puneetpal Singh, Valentin Yurievich Skryabin, Anna Aleksandrovna Skryabina, Amanda E Smith, Georgia Smith, Sameh S M Soliman, Soroush Soraneh, Reed J D Sorensen, Michael Spartalis, Bahadar S Srichawla, Panagiotis Stachteas, Antonina V Starodubova, Kurt Straif, Pete Stubbs, Vetriselvan Subramaniyan, Muritala Odidi Suleiman Odidi, Aleksander Sulkowski, Anusha Sultan Meo, Jing Sun, Zhong Sun, Sumam Sunny, Chandan Kumar Swain, Lukasz Szarpak, Rafael Tabarés-Seisdedos, Seyyed Mohammad Tabatabaei, Fatemeh Sadat Tabatabaei, Ozra Tabatabaei Malazy, Shima Tabatabai, Celine Tabche, Mohammad Tabish, Jabeen Taiba, Stella Talic, Mircea Tampa, Jacques Lukenze Tamuzi, Ker-Kan Tan, Manoj Tanwar, Saba Tariq, Nathan Y Tat, Seyed Mohammad Tavangar, Reem Mohamad Hani Temsah, Mohamad-Hani Temsah, Masayuki Teramoto, Dufera Rikitu Terefa, Jay Tewari, Rekha Thapar, Jansje Henny Vera Ticoalu, Sofonyas Abebaw Tiruneh, Tenaw Yimer Tiruye, Mariya Vladimirovna Titova, Krishna Tiwari, Sojit Tomo, Marcello Tonelli, Mathilde Touvier, Marcos Roberto Tovani-Palone, Khaled Trabelsi, Mai Thi Ngoc Tran, Thang Huu Tran, Nguyen Tran Minh Duc, Domenico Trico, Indang Trihandini, Thien Tan Tri Tai Truyen, Aristidis Tsatsakis, Gary Tse, Guesh Mebrahtom Tsegay, Munkhtuya Tumurkhuu, Sree Sudha T Y, Sok Cin Tye, Stefanos Tyrovolas, Aniefiok John Udoakang, Shahid Ullah, Saeed Ullah, Muhammad Umair, Umar Muhammad Umar, Lawan Umar, Brigid Unim, Dinesh Upadhya, Era Upadhyay, Jibrin Sammani Usman, Damla Ustunsoz, Asokan Govindaraj Vaithinathan, Jef Van den Eynde, Joe Varghese, Tommi Juhani Vasankari, Siavash Vaziri, Balachandar Vellingiri, Narayanaswamy Venketasubramanian, Madhur Verma, Anjul Verma, Georgios-Ioannis Verras, Simone Vidale, Victor E Villalobos-Daniel, Manish Vinayak, Vasily Vlassov, Theo Vos, Rade Vukovic, Mugi Wahidin, Mohammad Wahiduzzaman, Yanzhong Wang, Shu Wang, Cong Wang, Xingxin Wang, Mary Njeri Wanjau, Ahmed Bilal Waqar, Muhammad Waqas, Kosala Gayan Weerakoon, Fei-Long Wei, Anggi Lukman Wicaksana, Dakshitha Praneeth Wickramasinghe, Peter Willeit, Marcin W Wojewodzic, Tewodros Eshete Wonde, Utoomporn Wongsin, Qing Xia, Wanqing Xie, Suowen Xu, Xiaoyue Xu, Kazumasa Yamagishi, Yuichiro Yano, Haiqiang Yao, Amir Yarahmadi, Habib Yaribeygi, Subah Abderehim Yesuf, Dehui Yin, Dong Keon Yon, Naohiro Yonemoto, Chuanhua Yu, Chun-Wei Yuan, Deniz Yuce, Ismaeel Yunusa, Sojib Bin Zaman, Iman Zare, Michael Zastrozhin, Mohammed G M Zeariya, Xiaoyi Zhang, Liqun Zhang, Jingya Zhang, Zhiqiang Zhang, Casper J P Zhang, David X Zheng, Peng Zheng, Anthony Zhong, Claire Chenwen Zhong, Jiayan Zhou, Bin Zhu, Abzal Zhumagaliuly, Magdalena Zielińska, Ghazal Zoghi, Zhiyong Zou, Elric Zweck, Sa'ed H Zyoud, Christopher J L Murray†, Susan M Sawyer†, and Stein Emil Vollset†.

*Co-first authors

†Co-senior authors

Affiliations

Yong Loo Lin School of Medicine (M Ng PhD, N W Chew MD, Prof N Venketasubramanian MSc), Department of Medicine (B Chong MBBS), Cardiovascular Metabolic Translational Research Program (M

Dalakoti MPH), Saw Swee Hock School of Public Health (S Ramazanu PhD), Department of Surgery (K Tan PhD), National University of Singapore, Singapore, Singapore; Institute for Health Metrics and Evaluation (M Ng PhD, Prof E Gakidou PhD, J Lo BA, A J Ahlstrom MSc, N Ahmad BS, C M Antony MA, A Y Aravkin PhD, C Bisignano MPH, D Bryazka BA, R M Cogen BA, X Dai PhD, Prof R Dandona PhD, Prof L Dandona MD, Prof S D Dharmaratne MD, Prof S I Hay FMedSci, N J Kassebaum MD, Prof S S Lim PhD, P A Lindstedt MPH, T Mestrovic PhD, Prof A H Mokdad PhD, E C Mullany BA, Prof M Naghavi PhD, E M O'Connell BA, C Raggi MS, A E Smith MPA, G Smith MS, R J D Sorensen PhD, Prof T Vos PhD, C Yuan PhD, P Zheng PhD, Prof C J L Murray DPhil, Prof S E Vollset DrPH), Department of Health Metrics Sciences, School of Medicine (Prof E Gakidou PhD, A Y Aravkin PhD, X Dai PhD, Prof R Dandona PhD, Prof S D Dharmaratne MD, Prof S I Hay FMedSci, N J Kassebaum MD, Prof S S Lim PhD, A Misganaw PhD, Prof A H Mokdad PhD, Prof M Naghavi PhD, Prof T Vos PhD, P Zheng PhD, Prof C J L Murray DPhil, Prof S E Vollset DrPH), Department of Applied Mathematics (A J Ahlstrom MSc, A Y Aravkin PhD), School of Medicine (E J Boyko MD), Department of Anesthesiology & Pain Medicine (N J Kassebaum MD), Department of Global Health (R J D Sorensen PhD), University of Washington, Seattle, WA, USA; Department of Clinical Governance and Quality Improvement (Y H Abate MSc), Aleta Wondo General Hospital, Aleta Wondo, Ethiopia; Department of Legal and Economic Studies (C Abbafati PhD), Department of Public Health and Infectious Diseases (M S Cattaruzza PhD), La Sapienza University, Rome, Italy; Centre for Regenerative Medicine and Health (N Abbas PhD), Chinese Academy of Sciences, Hong Kong, China; Department of Neuroscience (N Abbas PhD), Department of Infectious Diseases and Public Health (I Elsohaby PhD, G Fekadu PhD), City University of Hong Kong, Hong Kong, China; Department of Orthopedic Surgery (M Abbasian MD), Thomas Jefferson University, Philadelphia, PA, USA; Department of Orthopaedic Surgery (M Abbasian MD), Department of Anesthesiology (Prof D Abtahi MD, S Salimi MD), Department of Internal Medicine (S Afaghi MD), Department of Biotechnology (S Aghamiri PhD), National Nutrition and Food Technology Research Institute (M Ajami PhD), Internal Medicine Department (H Borhany MD), Psychiatric Nursing and Management Department (F Ghadirian PhD), Faculty of Medicine (A Ghaffari Jolfayi MD), Social Determinants of Health Research Center (S Ghamari MD, A Kolahi MD, A Nikoobar BSc, M Rashidi MD), Food Technology Research (Z Hadian PhD), Research Institute for Endocrine Sciences (S Masrouri MD), Skull Base Research Center (I Mohammadzadeh MD), Research Center for Social Determinants of Health (M Niknam PhD), Department of Immunology (A Rasouli-Saravani PhD), Department of Medical Education (S Tabatabai PhD), Shahid Beheshti University of Medical Sciences, Tehran, Iran (M Khayamzadeh MD); Department of Epidemiology (S Abd ElHafeez DrPH), Tropical Health Department (R M Ghazy PhD), Alexandria University, Alexandria, Egypt; Department of Medical Laboratory Science (Prof W M Abdel-Rahman PhD), Department of Pharmacy Practice and Pharmacotherapeutics (Prof K H Alzoubi PhD, Prof H A Omar PhD), Department of Physiotherapy (A Arumugam PhD), Clinical Sciences Department (H J Barqawi MPhil, Prof M M Saber-Ayad PhD), Department of Basic Biomedical Sciences (Prof Y Bustanji PhD), Department of Basic Medical Sciences (M A Eladl PhD, W El-Huneidi PhD), Department of Clinical Sciences (Prof M M Ramadan PhD), College of Medicine (Prof B A Saddik PhD, Prof M A Saleh PhD), Sharjah Institute of Medical Sciences (F Saheb Sharif-Askari PhD), College of Pharmacy (Prof M H Semreen PhD), Research Institute of Medical & Health Sciences (Prof M H Semreen PhD), Department of Medicinal Chemistry (S S M Soliman PhD), University of Sharjah, Sharjah, United Arab Emirates; Department of Tropical Medicine and Infectious Diseases (S Abd-Elsalam PhD), Tanta University, Tanta, Egypt; Minimally Invasive Surgery Research Center (A Abdollahi MD, A Kabir MD), Health Management and Economics Research Center (J Arabloo PhD), School of Medicine (M Bastan MD, M Dodangeh MD), Department of Medical Laboratory Sciences (F

Dorostkar PhD), Department of Medicine (M Fotouhi MD), Department of Cardiology (A Ghaffari Jolfayi MD), Endocrine Research Center (A Karimi Behnagh MD), Department of Echocardiography (A Karimi Behnagh MD), Department of Obstetrics & Gynecology (P Khalili MD), Physiology Research Center (H Pazoki Toroudi PhD), Department of Physiology (H Pazoki Toroudi PhD), Center for Technology and Innovation in Cardiovascular Informatics (S Shool MD), Iran University of Medical Sciences, Tehran, Iran (M Moradi MD); Department of Medicine (Prof M Abdoun PhD), University of Setif Algeria, Sétif, Algeria; Department of Health, Sétif, Algeria (Prof M Abdoun PhD); Community and Maternity Nursing Unit (D M Abdulah MPH), Department of Pathology and Microbiology (M S Ahmed PhD), University of Duhok, Duhok, Iraq; National Institute of Epidemiology (R Abdulkader PhD), Indian Council of Medical Research, Chennai, India; Department of Physiotherapy (A Abdullahi PhD, A W Awotidebe PhD, J S Usman PhD), Department of Community Medicine (Prof M A Gadanya MD), Bayero University Kano, Kano, Nigeria; Department of Physiotherapy (A Abdullahi PhD), Federal University Wukari, Wukari, Nigeria; Department of Emergency Medicine (A Abedi MD), Department of Immunology (S Athari PhD), Department of Critical Care and Emergency Nursing (N Hanifi PhD), Zanjan University of Medical Sciences, Zanjan, Iran; School of Health Sciences (H M Abeywickrama PhD), Niigata University, Niigata, Japan; Midwifery Department (A Abie MSc), College of Medicine and Health Sciences (M M Adane PhD, H B Netsere MSc), Department of Midwifery (B A Alemayehu MSc), Department of Epidemiology and Biostatistics (A A Asrat MPH), Department Nutrition and Dietetics (M T Bayih MSc), Department of Adult Health Nursing (E A Mengistie MSc), Bahir Dar University, Bahir Dar, Ethiopia; Department of Family and Community Health (R G Aboagye MPH), Department of Population and Behavioural Sciences (H Amu PhD, E Manu PhD), Department of Epidemiology and Biostatistics (S A Bosoka MPhil, R K Dowou MPhil), University of Health and Allied Sciences, Ho, Ghana; Cardiovascular Research Center (S Abohashem MPH), Department of Orthopaedic Surgery (A Ebrahimi MD), Department of Medicine (D X Zheng MD), Massachusetts General Hospital, Boston, MA, USA (A Eighaei Sedeh MD, M Kim MD); Department of Radiology (S Abohashem MPH), Department of Pediatrics (S Aly MD), Division of Cardiovascular Medicine (G Chi MD), Division of Cardiology (I Y Elgendy MD), Department of Neurosurgery (M L Jalloh MD), T. H. Chan School of Public Health (S B Kankam MD), Department of Medicine (M Kokkorakis BSc), Department of Health Policy and Oral Epidemiology (Z S Natto DrPH), T.H. Chan School of Public Health (P M S Pradhan MD, E Zweck MD), Division of Global Health Equity (P Rohloff MD), Beth Israel Deaconess Medical Center (S Sharfaei MD), Joslin Diabetes Center (S Tye PhD), Harvard Medical School (A Zhong MA), Harvard University, Boston, MA, USA; Department of Nursing (H Abualruz PhD), Al Zaytoonah University of Jordan, Amman, Jordan; Department of Pharmacology and Toxicology (B Abubakar PhD), Department of Veterinary Public Health and Preventive Medicine (A Shittu MSc), Usmanu Danfodiyo University, Sokoto, Sokoto, Nigeria; Clinical Science Department (Prof M O Folayan PhD), Department of Biochemistry and Nutrition (K Oyebola PhD), Nigerian Institute of Medical Research, Lagos, Nigeria (B Abubakar PhD); Clinical Pharmacy and Therapeutics Department (Prof R K Abu Farha PhD), Department of Clinical Nutrition and Dietetics (Prof M E M Faris PhD), Applied Science Private University, Amman, Jordan; Medical Research Center (H J Abukhadijah MPH), Department of Surgery (A Alansari MD), Department of Geriatric and Long Term Care (H Al Hamad MD, B Sathian PhD), Rumailah Hospital (H Al Hamad MD), Research Department (K Singh PhD), Hamad Medical Corporation, Doha, Qatar; Institute of Community and Public Health (Prof N M Abu-Rmeileh PhD), Birzeit University, Ramallah, Palestine; Department of Pharmacology and Therapeutics (Prof S Aburuz PhD), Institute of Public Health (I Elbarazi DrPH, Prof M Grivna PhD, Prof S M Shah PhD), Family Medicine Department (M A Khan MSc), College of Medicine and Health Sciences (J Nauman PhD), United Arab Emirates University, Al Ain, United Arab

Emirates; College of Pharmacy (Prof S Aburuz PhD), School of Nursing (Prof M M Ahmad PhD), University of Jordan, Amman, Jordan; Department of Biochemistry and Molecular Medicine (A Abu-Zaid PhD), College of Medicine (Prof O Baltatu PhD), College of Pharmacy (R M H Temsah PharmD), Alfaisal University, Riyadh, Saudi Arabia; College of Graduate Health Sciences (A Abu-Zaid PhD), University of Tennessee, Memphis, TN, USA; Department of Diagnostic and Interventional Radiology (L C Adams PhD), School of Medicine and Health (F Busch MD), Technical University of Munich, Munich, Germany; School of Medicine (J Zhou PhD), Stanford University, Palo Alto, CA, USA (L C Adams PhD); School of Medicine (I Y Addo PhD), School of Architecture, Design, and Planning (Prof T Astell-Burt PhD), Concord Clinical School (R Chimoriya PhD), Faculty of Medicine and Health (W He PhD), University of Sydney, Sydney, NSW, Australia; Centre for Social Research in Health (I Y Addo PhD), School of Population Health (X Feng PhD, Prof B A Saddik PhD, Prof A E Schutte PhD, X Xu PhD), International Centre for Future Health Systems (J Lin PhD), School of Risk and Actuarial Studies (Y Si PhD), University of New South Wales, Sydney, NSW, Australia; Department of Immunology (K A Adedokun MSc), Roswell Park Comprehensive Cancer Center, Buffalo, NY, USA; Translational Research Team (N A Adegoke PhD), The University of Sydney, Sydney, NSW, Australia; Department of HIV and Infectious Diseases (A V Adepoju MD), Jhpiego, Abuja, Nigeria; Department of Adolescent Research and Care (A V Adepoju MD), Adolescent Friendly Research Initiative and Care, Ado Ekiti, Nigeria; Department of Veterinary Medicine and Surgery (R Adesola DVM), University of Missouri, Columbia, MO, USA; Department of Pharmacology and Therapeutics (T E Adeyeoluwa PhD), Department of Microbiology (I A Anuoluwa PhD, Y Oluwafemi PhD), Department of Environmental and Occupational Health (B S Anuoluwa MPH), Department of Biosciences and Biotechnology (A J Udoakang PhD), University of Medical Sciences, Ondo, Ondo, Nigeria; Department of Veterinary Medicine (T E Adeyeoluwa PhD), Parasitology Unit (A B Bello PhD), Department of Epidemiology and Medical Statistics (A F Fagbamigbe PhD), College of Medicine (A P Okekunle PhD), Department of Medicine (O V Olalusi MD, Prof M O Owolabi DrM), University of Ibadan, Ibadan, Nigeria; Apollo Institute Of Medical Sciences & Research Chittoor (Prof U Adiga PhD), Apollo Hospital, CHITTOOR, India; Department of Public Health (Q Adnani PhD), Padjadjaran University (Universitas Padjadjaran), Bandung, Indonesia; Department of Community Medicine (Prof S Afzal PhD), King Edward Memorial Hospital, Lahore, Pakistan; Department of Public Health (Prof S Afzal PhD), Public Health Institute, Lahore, Pakistan; Department of Life Sciences (M S Afzal PhD, Prof M Umair PhD), School of Sciences (M N Saqib PhD), University of Management and Technology, Lahore, Pakistan; Department of Community Medicine (T C Agampodi PhD), Department of Family Medicine (Prof D Rathish PhD), Department of Parasitology (Prof K G Weerakoon PhD), Rajarata University of Sri Lanka, Anuradhapura, Sri Lanka; Health Research and Innovation Sciences Center (C Agostinis Sobrinho PhD), Klaipeda University, Klaipeda, Lithuania; SPRINT Sport Physical Activity and Health Research & Innovation Center (C Agostinis Sobrinho PhD), Sport Physical Activity and Health Research & Innovation Center (SPRINT) (Prof L M R Silva PhD), Polytechnic Institute of Guarda, Guarda, Portugal; Department of Public Health Sciences (W Agyemang-Duah PhD), Queen's University, Kingston, ON, Canada; School of Medicine and Psychology (D Ahmad PhD), Australian National University, Canberra, ACT, Australia; Public Health Foundation of India, Gandhinagar, India (D Ahmad PhD); Department of Health and Biological Sciences (S Ahmad PhD), Abasyn University, Peshawar, Pakistan; Department of Natural Sciences (S Ahmad PhD), Gilbert and Rose-Marie Chagoury School of Medicine (Prof L Roever PhD), Lebanese American University, Beirut, Lebanon; College of Medicine (A Ahmad PhD, M Tabish MPharm), Department of Pharmacology (A R Pathan PhD), Shaqra University, Shaqra, Saudi Arabia; College of Applied Sciences (Prof F Ahmad PhD), Almaarefa University, Riyadh, Saudi Arabia; Department of

Biosciences (H Ahmed PhD), COMSATS Institute of Information Technology, Islamabad, Pakistan; Department of Epidemiology (M B Ahmed PhD, D Shiferaw MPH), Institute of Health Science (A I Mohamed MSc), Jimma University, Jimma, Ethiopia (B Feyisa MPH); College of Medicine and Public Health (M B Ahmed PhD, M Derbew Molla MSc, T G Gebremeskel PhD, A M Hopkins PhD, B Kaambwa PhD, G R Naik PhD, S Ullah PhD), Health Economics Unit (B Kaambwa PhD), Department of Nursing and Health Sciences (S Shorofi PhD), Flinders University, Adelaide, SA, Australia; Institute of Endemic Diseases (A Ahmed MSc), Unit of Basic Medical Sciences (E E Siddig MD), University of Khartoum, Khartoum, Sudan; Swiss Tropical and Public Health Institute (A Ahmed MSc), University of Basel, Basel, Switzerland; College of Nursing (M S Ahmed MSc), Majmaah University, Al Majmaah, Saudi Arabia; Brody School of Medicine (S Ahmed PhD), East Carolina University, Greenville, NC, USA; Institute of Global Health and Development (S Akhtar MPH), Aga Khan University, Karachi, Pakistan; Department of Cardiology (M Akkaif PhD), Fudan University, Shanghai, China; Chicago College of Osteopathic Medicine (A E Akrami BS), Midwestern University, Downers Grove, IL, USA; Feinberg School of Medicine (A E Akrami BS, A Keshwani MPH), Medical Scientist Training Program (S Marzouk MA), Department of Preventive Medicine (M Teramoto MD), Northwestern University, Chicago, IL, USA; Department of Biology (T A Alalwan PhD), College of Health and Sport Sciences (A G Vaithinathan MSc), University of Bahrain, Zallaq, Bahrain; Department of Research and Development (Z Al-Aly MD), Division of Public Health Sciences (S Al Hasan PhD), Department of Surgery (Y Cao DSc, C Wang PhD), Washington University in St. Louis, St. Louis, MO, USA; Clinical Epidemiology Center (Z Al-Aly MD), US Department of Veterans Affairs (VA), St. Louis, MO, USA; Murdoch Business School (K Alam PhD), Murdoch University, Perth, WA, Australia; School of Nursing (R M Al-amer PhD), Department of Basic Sciences (Z Altaany PhD), Department of Basic Medical Sciences (R A Karasneh PhD, Prof M M Khatatbeh PhD), Yarmouk University, Irbid, Jordan; School of Nursing and Midwifery (R M Al-amer PhD), Department of Engineering (G R Naik PhD), Translational Health Research Institute (K Rana PhD), Western Sydney University, Sydney, NSW, Australia; Department of Clinical Pharmacy (F Y Al-Ashwal PhD), Al-Ayen Iraqi University, Thi-Qar, Iraq; Department of Clinical Pharmacy and Pharmacy Practice (F Y Al-Ashwal PhD), University of Science and Technology, Sana'a, Yemen; Department of Community and Mental Health (Prof M Albashtawy PhD), Al al-Bayt University, Mafraq, Jordan; Division of Gastroenterology and Hepatology (W A Aldhaleei MD, A S Bhagavathula PhD), Mayo Clinic, Jacksonville, FL, USA; Department of Bacteriology, Immunology, and Mycology (Prof A M Algammal PhD), Suez Canal University, Ismailia, Egypt; Department of Cardiac Sciences (Prof K F Alhabib MD), Department of Physiology (Prof S A Meo PhD), University Diabetes Center (A Sultan Meo MPH), Pediatric Intensive Care Unit (Prof M Temsah MD), King Saud University, Riyadh, Saudi Arabia; Information Science Department (D Alhuwail PhD), Kuwait University, Sabah Alsalem University City, Kuwait; Health Informatics Unit and Geohealth Lab (D Alhuwail PhD), Dasman Diabetes Institute, Dasman, Kuwait; Department of Biosciences (R Ali PhD), Centre for Interdisciplinary Research in Basic Sciences (CIRBSc) (S Anwar PhD), Centre for Interdisciplinary Research in Basic Sciences (T Mohammad PhD), Centre For Interdisciplinary Research In Basic Sciences (CIRBSc) (A Shamsi PhD), Jamia Millia Islamia, New Delhi, India; Department of Zoology (A Ali PhD), Abdul Wali Khan University Mardan, Mardan, Pakistan; Department of Geography (W Ali PhD), Sultan Qaboos University, Muscat, Oman; Department of Medical Rehabilitation (Physiotherapy) (M U Ali PhD), University of Maiduguri, Maiduguri, Nigeria; Department of Rehabilitation Sciences (M U Ali PhD, M Khan MPH, J S Usman PhD), School of Nursing (S Tyrovolas PhD), Hong Kong Polytechnic University, Hong Kong, China; Institute of Health and Wellbeing (S M Alif PhD), Federation University Australia, Melbourne, VIC, Australia; School of Public Health and Preventive Medicine (S M Alif PhD, S

Talic PhD), School of Public Health and Preventative Medicine (Prof M Asghari-Jafarabadi PhD), Department of Epidemiology and Preventive Medicine (M A Awoke MPH, R M Islam PhD), Monash Centre for Health Research and Implementation (MCHRI) (S A Tiruneh MPH), Monash University, Melbourne, VIC, Australia; Department of Clinical and Community Pharmacy (Prof S W Al-Jabi PhD, Prof S H Zyoud PhD), An-Najah National University, Nablus, Palestine; Department of Public Health and Community Medicine (Prof S M Aljunid PhD), International Medical University, Kuala Lumpur, Malaysia; International Centre for Casemix and Clinical Coding (Prof S M Aljunid PhD), National University of Malaysia, Bandar Tun Razak, Malaysia; College of Life Sciences (Prof A Alkhatib PhD), Birmingham City University, Birmingham, UK; Independent Consultant, Amman, Jordan (S Al-Marwani MSc); Department of Physical Therapy and Rehabilitation Sciences (Prof M A Alomari PhD), Department of Rehabilitation Sciences and Physical Therapy (Prof M A Alomari PhD), Department of Allied Medical Sciences (A Alrawashdeh PhD), Department of Rehabilitation Sciences (M Al-Wardat PhD), Faculty of Medicine (A Alzoubi PhD), Department of Clinical Pharmacy (Prof K H Alzoubi PhD), Jordan University of Science and Technology, Irbid, Jordan; Liver, Digestive, and Lifestyle Health Research Section (S A Alqahtani MD), King Faisal Specialist Hospital & Research Center, Riyadh, Saudi Arabia; Division of Gastroenterology and Hepatology (S A Alqahtani MD), Weill Cornell Medicine, New York, NY, USA; Department of Community Medicine (R M M Al-Raddadi MD), Department of Family and Community Medicine (Prof N S Butt PhD), Rabigh Faculty of Medicine (Prof A Malik PhD), Department of Dental Public Health (Z S Natto DrPH), King Abdulaziz University, Jeddah, Saudi Arabia; Department of Nursing (I Alrimawi PhD), Department of Medicine (M Hemmati MD, C J Sabet MA), Georgetown University, Washington, DC, USA; Macro-Fiscal Policy Department (S M Alrousan PhD), Ministry of Finance, Dubai, United Arab Emirates; Department of Family and Community Medicine (N Z Alshahrani MD), University of Jeddah, Jeddah, Saudi Arabia; Department of Internal Medicine (O Al Ta'ani MD), Allegheny Health Network, Pittsburgh, PA, USA; Jordan Medical Association, Amman, Jordan (Z Al Ta'ani MD); Institute of Molecular Biology and Biotechnology (A Altaf PhD, S Shahid PhD), University College of Medicine & Dentistry (Prof M Arooj PhD), University Institute of Food Science and Technology (S Bashir PhD), Research Centre for Health Sciences (RCHS) (S Shahid PhD), Faculty of Sciences (Prof A B Waqar PhD), The University of Lahore, Lahore, Pakistan; Faculty of Pharmacy (Y Al Thaher PhD), Philadelphia University, Amman, Jordan; School of Pharmacy (Y Al Thaher PhD), Cardiff University, Cardiff, UK; Research Group in Health Economics (Prof N Alvis-Guzman PhD), Universidad de Cartagena (University of Cartagena), Cartagena, Colombia; Research Group in Hospital Management and Health Policies (Prof N Alvis-Guzman PhD), Universidad de la Costa (University of the Coast), Barranquilla, Colombia; Department of Medical Sciences (Prof Y M Al-Worafi PhD), Azal University for Human Development, Sana'a, Yemen; Department of Clinical Sciences (Prof Y M Al-Worafi PhD), University of Science and Technology of Fujairah, Fujairah, United Arab Emirates; Department of Pediatric Cardiology (S Aly MD), Boston Children's Hospital, Boston, MA, USA; Department of Pediatrics (Prof H Aly MD), Department of Internal Medicine (J Bhalla MD, U Saraswati MD), Cleveland Clinic, Cleveland, OH, USA; Department of Physiotherapy (H Alzahrani PhD), Taif University, Taif, Saudi Arabia; Department of Pathological Sciences (A Alzoubi PhD), College of Medicine (S Dutta PhD), Center for Medical and Bio-Allied Health Sciences Research (Prof M J Shahwan PhD, A Shamsi PhD), Ajman University, Ajman, United Arab Emirates (Prof N Hassan PhD); Maternal and Child Health Division (MCHD) (M Al-Zubayer MSc), Maternal and Child Health Division (R Banik MS), International Centre for Diarrhoeal Disease Research, Bangladesh (icddr,b), Dhaka, Bangladesh; Spiritual Health Research Center (S Amiri PhD), Baqiyatallah University of Medical Sciences, Tehran, Iran; Department of Maternal and Child Wellbeing (D A Amugsi PhD), African Population and Health Research

Center, Nairobi, Kenya; Department of Medicine (G A Amusa MD), University of Jos, Jos, Nigeria; Department of Internal Medicine (G A Amusa MD), Jos University Teaching Hospital, Jos, Nigeria; Department of General Medicine (R A Ananda MD), Eastern Health, Box Hill, VIC, Australia; Faculty of Pharmacy (Prof R Ancuceanu PhD), Department of Cardiology (Prof C Andrei PhD), Department of Legal Medicine and Bioethics (Prof S Hostiuc PhD), Department of Internal Medicine (M Hostiuc PhD), Department of Anatomy and Embryology (R I Negoi PhD), Department of General Surgery (I Negoi PhD), Department of Dermatology (M Tampa PhD), Carol Davila University of Medicine and Pharmacy, Bucharest, Romania; Department of Diabetology (R M Anjana PhD), Madras Diabetes Research Foundation, Chennai, India; Department of Physiotherapy, School of Allied Health Sciences (S Ansari PhD), Galgotias University, Greater Noida, India; School of Pharmacy (M T Ansari PhD), University of Nottingham Malaysia, Semenyih, Malaysia; Regenerative Medicine, Organ Procurement and Transplantation Multi-disciplinary Center (S Anvari MD), Pediatric Diseases Research Center (R Bayat MSc, A Hassanzade Rad PhD), Gastrointestinal and Liver Disease Research Center (B Eftekhari MD), Department of Social Medicine and Epidemiology (A Feizkhah MD), Gastrointestinal and Liver Diseases Research Center (S Hassanipour PhD, F Sheida MD), Caspian Digestive Disease Research Center (S Hassanipour PhD), Guilan University of Medical Sciences, Rasht, Iran; School of Chemical and Life Sciences (SCLS) (S Anwar PhD), Jamia Hamdard, New Delhi, India; Rural Health Research Institute (A E Anyasodor PhD, S B Aychiluhm MPH, Prof J Sun PhD), Charles Sturt University, Orange, NSW, Australia; School of Medicine and Public Health (G C Apostol MD), Ateneo De Manila University, Pasig City, Philippines; Inter-Agency Committee on Environmental Health (G C Apostol MD), Department of Health Philippines, Manila, Philippines; Division of Gastroenterology, Hepatology, and Nutrition (J Arab MD), Division of Infectious Diseases (P R Ching MD), Virginia Commonwealth University, Richmond, VA, USA; Gastroenterology Department (J Arab MD), Pontifical Catholic University of Chile, Santiago, Chile; College of Pharmacy (M Arafat PhD), Al Ain University, Abu Dhabi, United Arab Emirates; College of Art and Science (D Areda PhD), Ottawa University, Surprise, AZ, USA; School of Life Sciences (D Areda PhD), Arizona State University, Tempe, AZ, USA; Faculty of Nursing (H Arifin M.Kep.), Division of Epidemiology (K D Artanti DrPH), Universitas Airlangga (Airlangga University), Surabaya, Indonesia; Department of Medical Laboratory Sciences (M Arkew MSc), Department of Public Health (S D Darcho MPH), School of Public Health (A Oumer PhD), Haramaya University, Harar, Ethiopia; Department of Cardiovascular, Endocrine-Metabolic Diseases and Aging (B Armocida MD), Istituto Superiore di Sanità (ISS), Rome, Italy; Department of Neurobiology, Care Sciences and Society (Prof J Ärnlöv PhD), Karolinska Institutet, Stockholm, Sweden; School of Health and Social Studies (Prof J Ärnlöv PhD), Dalarna University, Falun, Sweden; Institute for Biomedical Problems (A A Artamonov PhD), K.A. Timiryazev Institute of Plant Physiology (M V Titova PhD), Russian Academy of Sciences, Moscow, Russia; Department of Physiotherapy (A Arumugam PhD), Department of Forensic Medicine and Toxicology (S M Bakkannavar MD), Department of Pharmacology (S Gangachannaiah MD, R R Shenoy PhD), Kasturba Medical College, Mangalore (R Holla MD), Kasturba Medical College Manipal (J P Raj DM), Kasturba Medical College Mangalore (M Rao MD), Kasturba Medical College (D Upadhya PhD), Manipal Academy of Higher Education, Manipal, India; Cabrini Research (Prof M Asghari-Jafarabadi PhD), Cabrini Health, Malvern, VIC, Australia; Pioneer Journal of Biostatistics and Medical Research (PJBMR), Pakistan, Pakistan (T Ashraf PhD); Deakin Health Economics/School of Health and Social Development (B K Y Asiamah-Asare PhD), Institute for Physical Activity and Nutrition (S Paudel PhD), Deakin University, Melbourne, VIC, Australia; School of Medicine and Public Health (P Atorkey PhD), University of Newcastle, Newcastle, NSW, Australia; Australian College of Applied Professions (P Atorkey PhD), Australian College of Applied

Professions, Sydney, NSW, Australia; Department of Forensic Medicine (A Atreya MD), Department of Community Medicine (S Nepal MD), Lumbini Medical College, Palpa, Nepal; кафедра (Z M Aumoldaeva MSc), Al Farabi Kazakh National University, Almaty, Kazakhstan; Department of Health Sciences (H Awad PhD), Higher College of Technology, Abu Dhabi, United Arab Emirates; School of Nursing and Public Health (A W Awotidebe PhD), University of KwaZulu-Natal, Durban, South Africa; Institute of Public Health (S B Aychiluhm MPH), Department of Biochemistry (M Derbew Molla MSc), School of Nursing (H B Netsere MSc), Department of Clinical Pharmacy (A K Sendekie MSc), University of Gondar, Gondar, Ethiopia; Nursing (A Azargoonjahromi BSc), Nutrition Research Center (M Babashahi PhD), Department of Pathology (S Faraji PhD), Research Center for Traditional Medicine and History of Medicine (Prof M Hashempur PhD), Trauma Research Center (M Karajizadeh PhD), Health Policy Research Center (M Nouri PhD), Non-communicable Disease Research Center (S G Sepanlou MD), Department of Medicine (A Yarahmadi PhD), Shiraz University of Medical Sciences, Shiraz, Iran; Department of Medicine (A Azimi MD, F Tabatabaei MD), Non-communicable Diseases Research Center (M Bastan MD, S Ghamari MD, M Keykhaei MD, M Rashidi MD, N Rezaei MD), Iranian Research Center for HIV/AIDS (IRCHA) (O Dadras PhD), School of Medicine (H Farrokhpour MD, S Khanmohammadi MD), Department of Surgery (R Foroumadi MD), Sina trauma and surgery research center (M L Jalloh MD), Cardiovascular Diseases Research Institute (Y Karimi MD), Students' Scientific Research Center (SSRC) (M Keykhaei MD), Children's Medical Center (Prof F Kompani MD), Endocrinology and Metabolism Research Institute (Prof B Larijani MD, O Tabatabaei Malazy PhD), Department of Physical Medicine and Rehabilitation (N Maghbouli MD), Department of Cardiology (E Mahmoudi MD), Department of Cardiovascular Diseases (S Nematollahi MD), Digestive Diseases Research Institute (Prof A Pourshams MD, S G Sepanlou MD), Non-Communicable Diseases Research Center (S Saeedi Moghaddam MSc), Research Center for Immunodeficiencies (A Saghazadeh MD), Department of Neurology (M Shafie MD), Sina Hospital (A Sharifan PharmD), Department of Endocrinology and Metabolism Population Sciences (A Sheikhy MD), Sina Trauma and Surgery Research Center (S Shool MD), Department of Pathology (Prof S Tavangar MD), Tehran University of Medical Sciences, Tehran, Iran (M Mahalleh MD); Department of Anesthesia (S A Aziz PhD), Cihan University -Sulaimaniya, Sulaymaniyah, Iraq; Department of Basic Sciences (S A Aziz PhD), College of Science (F M Rahman PhD), University of Sulaimani, Sulaymaniyah, Iraq; Institute of Biotechnology and Genetic Engineering (S Aziz MS), The University of Agriculture, Peshawar, Pakistan; ASIDE Healthcare, Lewes, DE, USA (A Azzam MD); Faculty of Medicine (A Azzam MD), October 6 University, 6th of October City, Egypt; Geriatric Unit (D Azzolino PhD), Fondazione IRCCS Ca' Granda Ospedale Maggiore Policlinico, Milan, Italy; Centre for Adolescent Health (P S Azzopardi PhD, K I Cini MClinEpi, K L Francis MBiostat, J A Kerr PhD, Prof S M Sawyer MD), Population Health Theme (Prof G C Patton MD), Murdoch Children's Research Institute, Parkville, VIC, Australia; Department of Paediatrics (P S Azzopardi PhD, Prof G C Patton MD), Population Interventions Unit (B Dhungel DrPH), Department of Medicine (Prof F K Jebasingh DM), University of Melbourne, Melbourne, VIC, Australia; Department of Population Medicine (Prof G Babu PhD), Department of Rehabilitation Sciences (S F Kanaan PhD), QU Health (M Mohammed PhD), Social and Economic Survey Research Institute (Prof A Perianayagam PhD), Qatar University, Doha, Qatar; Department of Forensic Science (A D Badiye PhD, N Kapoor PhD), Government Institute of Forensic Science Nagpur, Nagpur, India; Rashtrasant Tukadoji Maharaj Nagpur University, Nagpur, India (A D Badiye PhD); Health Research Institute (Prof N Bagheri PhD), University of Canberra, Canberra, ACT, Australia; Community Medicine (Y Bahurupi MD), All India Institute of Medical Sciences, Nagpur, India; Clinical Research Center (R Bai MD), Children's Hospital of Nanjing Medical University, Nanjing, China; International Medical School (A A Baig PhD), Management and Science

University, Alam, Malaysia; Division of Biological Sciences (S Balakrishnan PhD), Tamil Nadu State Council for Science and Technology, Chennai, India; Center of Innovation, Technology and Education (CITE) (Prof O Baltatu PhD), Anhembi Morumbi University, São José dos Campos, Brazil; Department of Medicine (K Bam MPH), Department of Anatomy and Developmental Biology (Y Mathangasinghe PhD), Monash University, Clayton, VIC, Australia; Department of Hypertension (Prof M Banach PhD), Medical University of Lodz, Lodz, Poland; Polish Mothers' Memorial Hospital Research Institute, Lodz, Poland (Prof M Banach PhD); Miller School of Medicine (M Bardhan MD), University of Miami, Miami, FL, USA; Center for Nutrition and Health Research (I Campos-Nonato PhD, E Denova-Gutiérrez DSc), National Institute of Public Health, Cuernavaca, Mexico (S Barquera PhD); Department of Non-communicable Diseases (L Barua MPH), Bangladesh University of Health Sciences, Dhaka, Bangladesh; Alpha Genomics Private Limited, Islamabad, Pakistan (Z Basharat PhD); Department of Academics (S Basu MD), Indian Institute of Public Health, Gurgaon, India; Human Anatomy and Histology (Prof N Beeraka PhD), Department of Epidemiology and Evidence-Based Medicine (R V Polibin PhD), I.M. Sechenov First Moscow State Medical University, Moscow, Russia; Center of Research Excellence in Stillbirth (T Begum PhD), Poche Centre for Indigenous Health (Prof A A Mamun PhD), The University of Queensland, Brisbane, QLD, Australia (M Moni PhD); Health System and Population Studies Division (T Begum PhD), Maternal and Child Health Division (S Noor MS), International Centre for Diarrhoeal Disease Research, Bangladesh, Dhaka, Bangladesh; Department of Physiotherapy and Paramedicine (U M Bello PhD), Glasgow Caledonian University, Glasgow, UK; Department of Biological Sciences (Prof L Belo PhD), Research Unit on Applied Molecular Biosciences (UCIBIO) (Prof L Belo PhD), Associated Laboratory for Green Chemistry (LAQV) (M Carvalho PhD), Institute for Research and Innovation in Health (i3S) (Prof N Cruz-Martins PhD), Applied Molecular Biosciences Unit (Prof C Fortuna Rodrigues PhD), Faculty of Engineering (Prof C Fortuna Rodrigues PhD), Department of Chemical Sciences (A Reis-Mendes PhD), Faculty of Medicine (J R Rocha-Gomes MD), University of Porto, Porto, Portugal; Department of Internal Medicine (I M Bensenor PhD), Center for Clinical and Epidemiological Research (A B Oliveira PhD), University of São Paulo, São Paulo, Brazil; Dipartimento di Scienze Mediche e Chirurgiche (M Bergami PhD), Department of Medical and Surgical Sciences (Prof R Bugiardini MD, E Cenko MD, M Sassano MD), University of Bologna, Bologna, Italy; Department of Nutrition and Dietetics (K Berhe MPH), Mekelle University, Mekelle, Ethiopia; School of public health (A A Berihun MA), Department of Biostatistics (A Columbus MS), Department of Epidemiology (T G Hundie MD), Johns Hopkins University, Baltimore, MD, USA; Community and Family Medicine (A S Bhadoria MD), All India Institute of Medical Sciences, Rishikesh, India; Community Health (A S Bhadoria MD), University of South Wales, South Wales, UK; Department of Public Health (A S Bhagavathula PhD), North Dakota State University, Fargo, ND, USA; Institute of Applied Health Research (N Bhala PhD), University of Nottingham, Nottingham, UK; Institute of Applied Health Research (N Bhala PhD, K Malhotra MBBS), NIHR Global Health Research Unit on Global Surgery (S K Kamarajah MD), Department of Metabolism and Systems Science (S Tariq PhD), University of Birmingham, Birmingham, UK; Department of Cardiovascular Medicine (J Bhalla MD, U Saraswati MD), Mayo Clinic Foundation for Medical Education and Research, Rochester, MN, USA; Department of Medicine (R Bharadwaj PhD), Department of Neurology (B S Srichawla MD), University of Massachusetts Medical School, Worcester, MA, USA; Department of Community Medicine and Family Medicine (Prof P Bhardwaj MD), School of Public Health (Prof P Bhardwaj MD), Department of Anatomy (N Bhardwaj MD), Department of Pharmacology (A Saravanan MD, M Shamim MBBS, S Singh MD, K Tiwari MBBS), Department of Biochemistry (S Tomo MD), All India Institute of Medical Sciences, Jodhpur, India; Global Health Neurology Lab (S Bhaskar MD), NSW Brain Clot Bank, Sydney, NSW, Australia; Division of

Cerebrovascular Medicine and Neurology (S Bhaskar MD), National Cerebral and Cardiovascular Center, Suita, Japan; Department of General Medicine (A N Bhat MD), Department of Community Medicine (N Joseph MD, R Motappa MD, R Thapar MD), Department of Forensic Medicine and Toxicology (Prof J Padubidri MD, P H Shetty MD), Manipal Academy of Higher Education, Mangalore, India; The Translational and Clinical Institute (P Bhattacharjee MD), Newcastle University, Newcastle upon Tyne, UK; Department of Clinical Medicine (P Bhattacharjee MD), Cambridge University Hospitals NHS Foundation Trust, Cambridge, UK; School of Sport & Health Sciences (S Bhattacharjee MPH), University of Brighton, Brighton, UK; Department of Public Health Research (S Bhattacharjee MPH), Bengal Rural Welfare Service (BRWS), Kolkata, India; Department of Human Genetics and Molecular Medicine (Prof J S Bhatti PhD, U Sharma PhD), Department of Microbiology (A Singh PhD), Department of Biochemistry (B Singh PhD), Department of Zoology (B Vellingiri PhD), Central University of Punjab, Bathinda, India; Department of Medical Lab Technology (Prof G K Bhatti PhD), University Centre for Research and Development (S Kalra DM), Chandigarh University, Mohali, India; Division of Infection, Immunity and Respiratory Medicine (A Bikov PhD), Division of Psychology and Mental Health (M R Radojčić PhD), University of Manchester, Manchester, UK; Department of Pulmonology (A Bikov PhD), Semmelweis University, Budapest, Hungary; Department of Radiology (C Bilgin MD), Neurovascular Research Laboratory (C Bilgin MD), Mayo Clinic College of Medicine, Rochester, MN, USA; Department of Community Medicine and Family Medicine (B Biswas MD), Department of Pharmacology (S T Y MD), All India Institute of Medical Sciences, Deoghar, India; Health and Social Research Center (B Bizzozero Peroni PhD), Universidad de Castilla-La Mancha, Cuenca, Spain; Higher Institute of Physical Education (B Bizzozero Peroni PhD), Universidad de la República, Rivera, Uruguay; Department of Community Medicine and Global Health (Prof E Bjertness PhD), University of Oslo, Oslo, Norway; Department of Global Public Health and Primary Care (Prof T Bjørge PhD), Department of Psychosocial Science (Prof D Sagoe PhD), University of Bergen, Bergen, Norway; Department of Research (M W Wojewodzic PhD), Cancer Registry of Norway, Oslo, Norway (Prof T Bjørge PhD); Department of Biomedical Sciences (S Bolla PhD), Nazarbayev University, Astana, Kazakhstan; Disease Surveillance Department (S A Bosoka MPhil), Ghana Health Service, Ho, Ghana; Department of Medicine (Prof S Bouaoud DrPH), Faculty of Medicine (Prof A Ouyahia PhD), University Ferhat Abbas of Setif, Setif, Algeria; Department of Epidemiology and Preventive Medicine (Prof S Bouaoud DrPH), University Hospital Saadna Abdenour, Setif, Algeria; General Medicine Service (E J Boyko MD), Department of Veterans Affairs, Seattle, WA, USA; Department of Epidemiology (D Braithwaite PhD), University of Florida, Gainesville, FL, USA; Cancer Population Sciences Program (D Braithwaite PhD), University of Florida Health Cancer Center, Gainesville, FL, USA; Department of Sports and Computer Science (J Brazo-Sayavera PhD), Universidad Pablo de Olavid (Pablo de Olavide University), Seville, Spain; Division of Clinical Epidemiology and Aging Research (Prof H Brenner MD), German Cancer Research Center, Heidelberg, Germany; Center for Neuroscience (G Britton PhD), Institute for Scientific Research and High Technology Services, Panama City, Panama; Gorgas Memorial Institute for Health Studies, Panama City, Panama (G Britton PhD); College of Health Sciences (L P Bui PhD), VinUniversity, Hanoi, Vietnam; Research Advancement Consortium in Health, Hanoi, Vietnam (L P Bui PhD); Department of Radiology (F Busch MD), Department of Public Health and Primary Care (M Dalakoti MPH, Prof P Willeit PhD), University of Cambridge, Cambridge, UK; School of Pharmacy (Prof Y Bustanji PhD), The University of Jordan, Amman, Jordan; School of Public Health Sciences (Z A Butt PhD), University of Waterloo, Waterloo, ON, Canada; Al Shifa School of Public Health (Z A Butt PhD), Al Shifa Trust Eye Hospital, Rawalpindi, Pakistan; Department of Clinical Pharmacy (Prof D Calina PhD), University of Medicine and Pharmacy of Craiova,

Romania, Craiova, Romania; Institute of Biomedical Engineering (Prof L A Campos PhD), Anhembi Morumbi University, Sao Jose dos Campos, Brazil; Department of Biomedical Engineering (Prof L A Campos PhD), São José dos Campos Technology Park, Sao Jose dos Campos, Brazil; Department of Anesthesiology (S Cao MD), Third Xiangya Hospital of Central South University, Changsha, China; Unit of Hygiene and Public Health (A Capodici MD), Romagna Local Health Authority, Forlì-Cesena, Italy; Interdisciplinary Research Center for Health Science (A Capodici MD), Sant'Anna School of Advanced Studies, Pisa, Italy; IMPInstitute for Mental and Physical Health and Clinical Translation (IMPACT) (A F Carvalho MD), Deakin University, Geelong, VIC, Australia; Faculty of Health Sciences (M Carvalho PhD), University Fernando Pessoa, Porto, Portugal; Department of Pharmacological and Biomolecular Sciences (Prof A L Catapano PhD), Department of Food, Environmental and Nutritional Sciences (I Cioffi PhD), Department of Clinical Sciences and Community Health (Prof C La Vecchia MD), University of Milan, Milan, Italy; MultiMedica Sesto San Giovanni IRCCS, Sesto San Giovanni, Italy (Prof A L Catapano PhD); Graduate Program in Nutrition and Health (M Cattafesta PhD), Federal University of Espírito Santo, Vitoria, Brazil; Department of Medical, Surgical, and Health Sciences (Prof L Cegolon PhD, Prof M D'Oria MD), University of Trieste, Trieste, Italy; Public Health Unit (Prof L Cegolon PhD), University Health Agency Giuliano-Isontina (ASUGI), Trieste, Italy; Department of Nutrition (Prof F Cembranel DSc), Department of Physical Education (Prof D A S Silva PhD), Federal University of Santa Catarina, Florianópolis, Brazil; Mary MacKillop Institute for Health Research (Prof E Cerin PhD), Australian Catholic University, Melbourne, VIC, Australia; School of Public Health (Prof E Cerin PhD), Department of Medicine (H Chou MSc), University of Hong Kong, Hong Kong, China; Regional Epidemiological Observatory Department (A Cernigliaro MSc), Sicilian Regional Health Authority, Palermo, Italy; Non-communicable Diseases Division (J Chadwick MD), National Institute of Epidemiology, Chennai, India; Department of Biotechnology (Prof C Chakraborty PhD), Adamas University, Kolkata, India; Institute for Skeletal Aging & Orthopedic Surgery (Prof C Chakraborty PhD), Hallym University, Chuncheon, South Korea; Department of Medicine and Therapeutic (R N C Chan MBChB), Prince of Wales Hospital, Hong Kong, China; College of Medicine (J Chang PhD), Institute of Epidemiology and Preventive Medicine (Y L Samodra PhD), National Taiwan University, Taipei, Taiwan; Department of Nursing (J Chang PhD), National Taiwan University Hospital, Taipei, Taiwan; Temerty Faculty of Medicine (V Chattu MD), University of Toronto, Toronto, ON, Canada; Department of Community Medicine (V Chattu MD), Datta Meghe Institute of Medical Sciences, Sawangi, India; Department of Biology (A A Chaudhary PhD), Al-Imam Mohammad Ibn Saud Islamic University, Riyadh, Saudi Arabia; Department of Oral Medicine and Radiology (Prof A Chaurasia MD), Internal Medicine Department (J Tewari MBBS), King George's Medical University, Lucknow, India; Department of Stomatology (G Chen DMD), Department of Occupational and Environmental Health (H Lai PhD), Huazhong University of Science and Technology, Wuhan, China; Hubei Province Key Laboratory of Oral and Maxillofacial Development and Regeneration, Wuhan, China (G Chen DMD); Fuwai Hospital (A Chen PhD), Peking Union Medical College Hospital (J Qiu MD), Chinese Academy of Medical Sciences, Beijing, China; Clinical Research Center (H Chen PhD), Zhujiang Hospital of Southern Medical University, Guangzhou, China; Department of Paediatrics (E T W Cheng MBChB), Faculty of Medicine (J Huang MD), Jockey Club School of Public Health and Primary Care (C Zhong PhD), The Chinese University of Hong Kong, Hong Kong, China; Concord Institute of Academic Surgery (R Chimoriya PhD), Sydney Local Health District, Sydney, NSW, Australia; Cancer Big Data Center (D Choi PhD), National Cancer Center, Goyang, South Korea; Centre for Research Impact & Outcome (H Chopra PhD), Chitkara University, Rajpura, India; Department of Biosciences (S Chopra MPH), Center for Global Health Research (Prof A Sahebkar PhD), Saveetha Dental College and Hospitals (S Selvaraj PhD, M

Tovani-Palone PhD), Centre for Global Health Research (M Tabish MPharm), Saveetha University, Chennai, India; Department of Community Medicine (Prof S G Choudhari MD), Jawaharlal Nehru Medical College, Wardha, India; The Interdisciplinary Research Group on Biomedicine and Health (D Chu PhD), Faculty of Applied Sciences (D Chu PhD), VNU International School (VNUIS), Hanoi, Vietnam; Department of Health Behavior (S Chung MPH), Department of Biomedical Engineering (M Negahdary PhD), Center for Remote Health Technologies & Systems (M Negahdary PhD), Texas A&M University, College Station, TX, USA; Department of Health Informatics (S Chung PhD), Brain Sciences (Prof M Kivimäki PhD), University College London, London, UK; Health Data Research UK, London, UK (S Chung PhD); School of Nursing and Midwifery (M Chutiyami PhD), Discipline of Physiotherapy (P Stubbs PhD), University of Technology Sydney, Sydney, NSW, Australia; Department of Paediatrics (K I Cini MClinEpi), Centre for Adolescent Health (Prof S M Sawyer MD), University of Melbourne, Parkville, VIC, Australia; Sports Science Research Centre (D Collado-Mateo PhD), Rey Juan Carlos University, Madrid, Spain; Physical Activity and Health Institute (D Collado-Mateo PhD), Autonomous University of Chile, Talca, Chile; Department of Cardiovascular Sciences (N Conrad PhD, A Schuermans BSc, J Van den Eynde BSc), Faculty of Medicine (A Schuermans BSc), Katholieke Universiteit Leuven, Leuven, Belgium; Department of Family Medicine and Public Health (Prof M H Criqui MD), University of California San Diego, La Jolla, CA, USA; Department of Diagnostic and Therapeutic Technologies (Prof N Cruz-Martins PhD), Cooperativa de Ensino Superior Politécnico e Universitário (Polytechnic and University Higher Education Cooperative), Vila Nova de Famalicão, Portugal; Department of Public Health, Environments, and Society (Prof S Cummins PhD), Department of Non-Communicable Disease Epidemiology (M Iwagami PhD), London School of Hygiene & Tropical Medicine, London, UK; Department of Medical and Surgical Sciences and Advanced Technologies "GF Ingrassia" (Prof E D'Amico MD), Department of General Surgery and Medical-Surgical Specialties (Prof G Isola PhD), University of Catania, Catania, Italy; Department of Brain Sciences (L D'Anna PhD), WHO Collaborating Centre for Public Health Education and Training (Q Lin MPH, D L Rawaf MD), Department of Surgery and Cancer (Prof E Mossialos PhD), Department of Primary Care and Public Health (Prof S Rawaf MD, C Tabche MSc), Imperial College London, London, UK; Cardio-Thoraco-Vascular Department (Prof M D'Oria MD), Azienda Sanitaria Universitaria Giuliano Isontina, Trieste, Italy; Research Center for Child Psychiatry (O Dadras PhD), Heart Center (V Kytö MD), University of Turku, Turku, Finland; Health Policy Research (M R Mathur PhD), Public Health Foundation of India, Gurugram, India (Prof R Dandona PhD, Prof L Dandona MD, G Kumar PhD, A Pandey PhD); Department of Internal medicine (P Danpanichkul MD), Texas Tech University, Lubbock, TX, USA; Environmental Health (R Darvishi Cheshmeh Soltani PhD), Arak University of Medical Sciences, Arak, Iran; School of Nursing (A da Silva PhD), Department of Maternal-Child Nursing and Public Health (Prof D C Malta PhD, Prof F P Matozinhos PhD, Prof A C Micheletti Gomide Nogueira de Sá MSc, E J S Prates BS), Vaccination Research Observatory (T Rodrigues da Silva PhD), Federal University of Minas Gerais, Belo Horizonte, Brazil; Population Health Research Center (Prof K Davletov PhD), Director of the Scientific and Technological Park (I R Fakhradiyev PhD), Laboratory of Experimental Medicine (T Fazylov MD), Science and Technology Park (A Ibrayeva PhD), Research and Publication Activity Division (M Kulimbet MSc), Science Department (A Shamsutdinova MD), Atchabarov Scientific-Research Institute of Fundamental and Applied Medicine (A Zhumagaliuly MD), Kazakh National Medical University, Almaty, Kazakhstan; School of Medicine (I Delgado-Enciso DSc), University of Colima, Colima, Mexico; Department of Research (I Delgado-Enciso DSc), State Cancerology Institute of Colima, IMSS-BIENESTAR, Colima, Mexico; Department of Biological Sciences (I Dergaa PhD), University of Manouba, Manouba, Tunisia; Department of Social Sciences (I Dergaa PhD), University of Jendouba, El Kef, Tunisia; Department of

Public Health (A T Desale MPH, S A Tiruneh MPH), Debre Tabor University, Debre Tabor, Ethiopia; Chettinad Hospital & Research Institute (Prof V Devanbu MD), Chettinad Academy of Research and Education, Chennai, India; JSS Medical College Department of Biochemistry (D Devegowda PhD), Department of Biochemistry (Prof A Prashant PhD), Jagadguru Sri Shivarathreeswara University, Mysuru, India; Department of Pharmacy (S Dewan PhD), United International University, Dhaka, Bangladesh; Pharmacology Division (S Dewan PhD), Center for Life Sciences Research Bangladesh, Dhaka, Bangladesh; Sheffield Teaching Hospitals NHS Foundation Trust, Sheffield, UK (A Dhali MBBS); Department of Community Medicine (Prof S D Dharmaratne MD), University of Peradeniya, Peradeniya, Sri Lanka; Research Department (M Dhimal PhD), Nepal Health Research Council, Kathmandu, Nepal; Institute of Occupational, Social and Environmental Medicine (M Dhimal PhD), Goethe University, Frankfurt am Main, Germany; Faculty of Science (Prof D Diaz PhD), National Autonomous University of Mexico, Mexico City, Mexico; Department of Experimental and Clinical Medicine (M Dinu PhD), University of Florence, Florence, Italy; Department of Public Health (S Dohare MD, A Mehmood PhD), Epidemiology Program (M Khan MD), College of Nursing and Health Sciences (M Shanawaz MD), College of Public Health and Tropical Medicine (J Varghese PhD), Jazan University, Jazan, Saudi Arabia; Department of Social Medicine and Health Care Organisation (Prof K G Dokova PhD), Medical University of Varna, Varna, Bulgaria; Faculty of Medicine (N Dolatkhah PhD), Social Determinants of Health Research Center (Prof S Mohammad-Alizadeh-Charandabi PhD), Midwifery Department (Prof S Mohammad-Alizadeh-Charandabi PhD), Tabriz University of Medical Sciences, Tabriz, Iran; Center for Health Sciences (C B do Prado MSc), Federal University of Espírito Santo, Vitória, Brazil; Independent Consultant, South Plainfield, NJ, USA (O P Doshi MS); Department of Cardiology (R P Doshi MD), Hackettstown Medical Center, Hackettstown, NJ, USA; Newton Medical Center, Sparta, NJ, USA (R P Doshi MD); Faculty of Health, Medicine and Life Sciences (FHML) (V S Dsouza MSc), Maastricht University, Maastricht, Netherlands; School of Stomatology (M Du PhD), Department of Periodontology (M Du PhD), Shandong University, Jinan, China; Postgraduate Program in Health Sciences (S C Dumith PhD), Federal University of Rio Grande, Rio Grande, Brazil; Allied Health and Human Performance (D Dumuid PhD), Department of Allied Health and Human Performance (T Y Tiruye PhD), University of South Australia, Adelaide, SA, Australia; Postgraduate Program in Epidemiology (Prof B B Duncan MD, Prof M I Schmidt MD), Department of Social Medicine (R Mattiello PhD), Federal University of Rio Grande do Sul, Porto Alegre, Brazil; Department of Conservative Dentistry with Endodontics (A M Dziedzic DSc), Medical University of Silesia, Katowice, Poland; Isenberg School of Management (A Eighaei Sedeh MD), University of Massachusetts Amherst, Amherst, MA, USA; Faculty of Science and Health (M Ekholuenetale PhD), University of Portsmouth, Hampshire, UK; Anatomy and Embryology (M A Eladl PhD), Department of Cardiology (Prof M M Ramadan PhD), Faculty of Pharmacy (Prof M A Saleh PhD), Mansoura University, Mansoura, Egypt; Almoosa College of Health Sciences, Al Ahsa, Saudi Arabia (R A El Arab PhD); Deanship of Preparatory Year and Supporting Studies (Prof S El-Ashker PhD), Division of Forensic Medicine (Prof R G Menezes MD), Imam Abdulrahman Bin Faisal University, Dammam, Saudi Arabia; Department of Public Health and Community Medicine (Prof I F El Bayoumy DrPH), Tanta University, Tanta City, Egypt; School of Public Health (Prof I F El Bayoumy DrPH), Texila American University, Guyana, Guyana; Division of Cardiovascular Medicine (I Y Elgendy MD), University of Kentucky, Lexington, KY, USA; Faculty of Medicine (M Elhadi MD), University of Tripoli, Tripoli, Libya; Houston Methodist Hospital, Houston, TX, USA (M Elhadi MD); College of Public Health and Health Informatics (Prof A A El-Metwally PhD), King Saud bin Abdulaziz University for Health Sciences, Riyadh, Saudi Arabia; Medical Genomics Research Department (Prof M Umair PhD), King Abdullah International

Medical Research Center, Riyadh, Saudi Arabia (Prof A A El-Metwally PhD); Egypt Center for Research and Regenerative Medicine (ECRRM), Cairo, Egypt (M A Elmonem PhD); School of Pharmacy and Pharmaceutical Sciences (M Elnaem PhD), Ulster University, Coleraine, UK; Deanery of Biomedical Sciences (R Elsheikh MD), Postgraduate School (U A Eze MD), College of Medicine and Veterinary Medicine (G Verras MSc), University of Edinburgh, Edinburgh, UK; Department of Animal Medicine (I Elsohaby PhD), Cardiovascular Department (Prof A M A Saad MD), Zagazig University, Zagazig, Egypt (Prof M I Husseiny PhD); Department of Pediatrics (C Eltaha MD), University of Texas, Dallas, TX, USA; Department of Public Health and Tropical Medicine (T I Emeto PhD), College of Medicine, Dentistry and Public Health (Prof R C Franklin PhD), James Cook University, Townsville, QLD, Australia; Gastroenterology and hepatology department (M Eslami MD), Department of Cardiovascular Medicine (A Kumar MD, H Pham MD), Department of Radiology (F Nugen PhD), Department of Physiology and Biomedical Engineering (F Pourghazi MD), Mayo Clinic, Rochester, MN, USA; Department of Ophthalmology (U A Eze MD), Federal Medical Centre, Asaba, Nigeria; Department of Electrical and Computer Engineering (H Fadavian MSc), Tarbiat Modares University, Tehran, Iran; Research Centre for Healthcare and Community (A F Fagbamigbe PhD), Faculty of Health and Life Sciences (O P Kurmi PhD), Coventry University, Coventry, UK; Department of Medicine (I R Fakhradiyev PhD), Department of Health Policy and Management (Prof J Kim PhD), Korea University, Seoul, South Korea (Prof M Shin PhD); Environmental Statistics Unit (C S e Farinha PhD), National Institute of Statistics, Lisbon, Portugal; Ecological Economics and Environmental Management (C S e Farinha PhD), NOVA University of Lisbon, Lisbon, Portugal; Department of Family Medicine (U Farooque MD), Luton & Dunstable University Hospital, Luton, UK; Endocrinology and Metabolism Research Institute (H Farrokhpour MD), Department of Epidemiology (S Khanmohammadi MD, S Nejadghaderi MD, S Rashedi MD), Department of Epidemiology and Biostatistics (S Khosravi MD), School of Medicine (A Sheikhy MD), Non-Communicable Diseases Research Center (NCDRC), Tehran, Iran; Department of Crop Science and Biotechnology (S A Fasusi PhD), Dankook University, Cheonan, South Korea; Department of Biology and Medicine (P Fazeli MSc), School of Public Health (A Haq BS), Brown University, Providence, RI, USA; Department of Pharmacy (G Fekadu PhD), Institute of Health Sciences (B Feyisa MPH), Department of Public Health (D R Terefa MSc), Wollega University, Nekemte, Ethiopia; National Institute of Environmental Health (X Feng PhD), Chinese Center for Disease Control and Prevention, Beijing, China; Center for Biotechnology and Fine Chemistry (J C Fernandes PhD), Catholic University of Portugal, Porto, Portugal; Cardiovascular Health and Imaging Laboratory (R Fernandez-Jimenez PhD), Centro Nacional de Investigaciones Cardiovasculares (CNIC), Madrid, Spain; Department of Cardiology (R Fernandez-Jimenez PhD), Hospital Clinico San Carlos, IdISSC, Madrid, Spain; Department of Social Sciences (Prof N Ferreira PhD), University of Nicosia, Nicosia, Cyprus; Institute of Public Health (F Fischer PhD), Charité Universitätsmedizin Berlin (Charité Medical University Berlin), Berlin, Germany; Center for Research in Indigenous Health (D Flood MD), Maya Health Alliance, Tecpán, Guatemala; Department of Internal Medicine (D Flood MD), University of Michigan, Ann Arbor, MI, USA; Institute of Gerontology (N A Foigt PhD), National Academy of Medical Sciences of Ukraine, Kyiv, Ukraine; Department of Child Dental Health (Prof M O Folayan PhD), Obafemi Awolowo University, Ile-Ife, Nigeria; Department of Cell Biology and Biotechnology (A A Fomenkov PhD), K.A. Timiryazev Institute of Plant Physiology, Moscow, Russia; Bariatric and Metabolic Institute (R Foroumadi MD), Cleveland Clinic, Cleveland, Ohio, USA; Department of Neuroscience (M Foschi MD), Multiple Sclerosis Research Center, Ravenna, Italy; Department of Biotechnological and Applied Clincial Sciences (M Foschi MD), University of L'Aquila, L'Aquila, Italy; Department of Radiology (M Fotouhi MD), University of Southern California, Los Angeles, CA, USA; Faculty of Physical Culture (A

Gába PhD), Palacký University Olomouc, Olomouc, Czech Republic; Department of Community Medicine (Prof M A Gadanya MD), Aminu Kano Teaching Hospital, Kano, Nigeria; Department of Community Medicine (Prof A M Gaidhane MD), Datta Meghe Institute of Medical Sciences, Wardha, India; Department of Food Technology (Y Galali ResM), Salahaddin University-Erbil, Erbil, Iraq; Department of Nutrition and Dietetics (Y Galali ResM), Cihan University-Erbil, Erbil, Iraq; Department of Medical Epidemiology (S Gallus PhD), Department of Environmental Health Sciences (A Lugo PhD), Mario Negri Institute for Pharmacological Research, Milan, Italy; School of Public Health (B Ganesan PhD), Institute of Health & Management, Australia, Melbourne, VIC, Australia; Human Nutrition Laboratory (W P Gastélum Espinoza MSc), Department of Human Nutrition Research (J A Magaña Gómez PhD), Autonomous University of Sinaloa, Culiacán, Mexico; Department of Health Sciences, Bachelor's degree in Nutrition (W P Gastélum Espinoza MSc), Autonomous University of Occident, Culiacán, Mexico; Department of Midwifery (M W Gebregergis MSc), Department of Medical Laboratory Sciences (H N Meles MSc), Adigrat University, Adigrat, Ethiopia; College of Medicine and Public Health (T G Gebremeskel PhD), Department of Nursing (A N Iyasu MSc, G M Tsegay MSc), Aksum University, Aksum, Ethiopia; Department of Public Health (L Getacher PhD), Debre Berhan University, Debre Berhan, Ethiopia; Family and Community Medicine Department (R M Ghazy PhD), King Khalid University, Abha, Saudi Arabia; Country Office (A U Gil PhD), World Health Organization (WHO), Astana, Kazakhstan; Adelaide Medical School (T K Gill PhD), University of Adelaide, Adelaide, SA, Australia; Third Department of Neurology (E V Gnedovskaya PhD), Research Center of Neurology, Moscow, Russia; Department of Health Systems and Policy Research (Prof M Golechha PhD), Indian Institute of Public Health, Gandhinagar, India; Department of Life Sciences, Health and Healthcare Professions (Prof D Golinelli MD), Link Campus University, Rome, Italy; Health Services Research, Evaluation and Policy Unit (Prof D Golinelli MD), AUSL della Romagna, Ravenna, Italy; Department of Public Health and Preventive Medicine (Prof M Grivna PhD), Charles University, Prague, Czech Republic; Department of Endocrinology (A Grover MD), National Institutes of Health, Bethesda, MD, USA; School of Population Health (Z Guan MPH), School of Public Health (T R Miller PhD), School of Pharmacy (A K Sendekie MSc), Curtin University, Perth, WA, Australia; Department of Epidemiology and Biostatistics (S Guan MD), Anhui Medical University, Hefei, China; Post Graduate School of Public Health (G Guarducci MD), University of Siena, Siena, Italy; Department of Clinical Science (M I M Gubari PhD), University of Sulaimani, Sulaimani, Iraq; Harrington Heart and Vascular Institute (A Guha MD), Department of Neonatology (I Qattea MD), Case Western Reserve University, Cleveland, OH, USA; Division of Cardiovascular Medicine (A Guha MD), Ohio State University, Columbus, OH, USA; Department of Community Medicine (D A Gunawardane MD, S N K Navaratna MD), University of Peradeniya, Kandy, Sri Lanka; Division of Epidemiology (Z Guo PhD), Vanderbilt University Medical Center, Nashville, TN, USA; Department of Preventive Cardiology & Medicine (Prof R Gupta MD), Eternal Heart Care Centre & Research Institute, Jaipur, India; Department of Medicine (Prof R Gupta MD), Mahatma Gandhi University Medical Sciences, Jaipur, India; Department of Nephrology (A K Gupta PharmD), Max Super Specialty Hospital, New Delhi, India; Non-communicable Diseases Division (NCD) (A K Gupta PharmD), Indian Council of Medical Research, New Delhi, India; Department of Cardiology (R Gupta MBBS), Lehigh Valley Health Network, Allentown, PA, USA; Department of Toxicology (S Gupta MSc), Shriram Institute for Industrial Research, Delhi, India; Faculty of Medicine Health and Human Sciences (Prof V K Gupta PhD), Australian Institute of Health Innovation (P Peprah MSc), Macquarie University, Sydney, NSW, Australia; Department of Biomedical Gerontology (R S Gutiérrez-Murillo PhD), Pontifical Catholic University of Rio Grande do Sul, Porto Alegre, Brazil; Research Unit (J Guzman-Esquivel PhD), Mexican Institute of Social Security, Colima,

Mexico; Department of Clinical Pharmacology and Medicine (Prof N R Hadi PhD), University of Kufa, Najaf, Iraq; Food and Nutrition Research Institute, Tehran, Iran (Z Hadian PhD); Biochemistry Department (Prof N M Hamdy PhD), Department of Entomology (A M Samy PhD), Medical Ain Shams Research Institute (MASRI) (A M Samy PhD), Ain Shams University, Cairo, Egypt; Department of Public Health (S Hameed PhD), Green International University, Lahore, Lahore, Pakistan; School of Health and Environmental Studies (Prof S Hamidi DrPH), Hamdan Bin Mohammed Smart University, Dubai, United Arab Emirates; University Centre for Rural Health (Northern Rivers) (M Hamiduzzaman PhD), University of Sydney, Lismore, NSW, Australia; Sakarya University, Turkey, Sakarya, Turkiye (A Hanif PhD); Centre for Neuromuscular and Neurological Disorders (Perron Institute) (Prof G J Hankey MD), The University of Western Australia, Perth, WA, Australia; Stroke Research Centre (Prof G J Hankey MD), Perron Institute for Neurological and Translational Science, Perth, WA, Australia; Faculty of Medicine (N I Harlianto MD), Julius Centre for Health Sciences and Primary Care (G A Kayode PhD), Julius Global Health (Prof T O Olanrewaju MSc), Utrecht University, Utrecht, Netherlands; Department of Radiology (N I Harlianto MD), University Medical Center Utrecht, Utrecht, Netherlands; Research Unit (J M Haro MD), Parc Sanitari Sant Joan de Deu, Barcelona, Spain; Department of Mental Health (J M Haro MD), Carlos III Health Institute (Prof R Tabarés-Seisdedos PhD), Biomedical Research Networking Center for Mental Health Network (CiberSAM), Madrid, Spain; Sekolah Tinggi Ilmu Kesehatan Indonesia Maju (Indonesian Advanced College of Health Sciences) (R K Hartono MPH), Institution of Public Health Sciences, Jakarta, Indonesia; Faculty of Nursing (F Hasan PhD, D S Romadlon PhD), Chulalongkorn University, Bangkok, Thailand; Department of Pharmacy (Prof M S Hasnain PhD), Marwadi University, Rajkot, India; Department of Neurology (Prof A Hassan MD), Cairo University, Cairo, Egypt; Skaane University Hospital (R J Havmoeller PhD), Skaane County Council, Malmö, Sweden; Faculty of Kinesiology (Prof J J Hebert PhD), University of New Brunswick, Fredericton, NB, Canada; School of Allied Health (Prof J J Hebert PhD), Murdoch University, Murdoch, WA, Australia; Independent Consultant, Santa Clara, CA, USA (G Heidari MD); Department of Medicine (M Hemmati MD), MedStar Health, Washington, DC, USA; Graduate School of Medicine (Y Hiraike PhD), Department of Global Health Policy (Prof S Nomura PhD, S K Rauniyar PhD), University of Tokyo, Tokyo, Japan; School of Dentistry (N Hoan DDS), Department of Allergy, Immunology and Dermatology (D H Nguyen MD), Hanoi Medical University, Hanoi, Vietnam; School of Medicine (M Hoang MD), Faculty of Medicine (T T Truyen MD), Tan Tao University, Long An, Vietnam; School of Social Sciences (P Hoogar PhD), The Apollo University, Chittoor, India; Department of Physics (A Hossain PhD), Department of Population Science and Human Resource Development (Prof M Rahman DrPH), University of Rajshahi, Rajshahi, Bangladesh; School of Health and Society (H Hosseinzadeh PhD), University of Wollongong, Wollongong, NSW, Australia; Department of Clinical Legal Medicine (Prof S Hostiuc PhD), National Institute of Legal Medicine Mina Minovici, Bucharest, Romania; Health and Environmental Risk Division (Z Htay PhD), National Institute for Environmental Studies, Japan, Tsukuba, Japan; Department of Psychology (C Hu PhD), Tsinghua University, Beijing, China; Artur Riggs Diabetes & Metabolism Research Institute (Prof M I Husseiny PhD), Cancer Prevention and Research Institute, Duarte, CA, USA; International Master Program for Translational Science (H Huynh BS), Nursing School (M Kurniasari PhD), Department of Global Health and Health Security (K Latief PhD), School of Nursing (A L Wicaksana MS), Taipei Medical University, Taipei, Taiwan; Department of Public Health (Prof I Iavicoli PhD), Università di Napoli Federico II (University of Naples Federico II), Naples, Italy; West Africa RCC (O S Ilesanmi PhD), Africa Centre for Disease Control and Prevention, Abuja, Nigeria; Department of Community Medicine (O S Ilesanmi PhD), Department of Neurology (O V Olalusi MD), Department of Medicine (Prof M O Owolabi DrM), University College Hospital, Ibadan, Ibadan,

Nigeria; Faculty of Medicine (I M Ilic PhD, Prof M M Santric-Milicevic PhD), School of Public Health and Health Management (Prof M M Santric-Milicevic PhD), School of Medicine (R Vukovic PhD), University of Belgrade, Belgrade, Serbia; Faculty of Medical Sciences (Prof M D Ilic PhD), University of Kragujevac, Kragujevac, Serbia; Department of Clinical Pharmacy (M Imam PhD), Department of Electrical Engineering (I Malik PhD), Department of Health and Rehabilitation Sciences (Prof G Nambi PhD), Prince Sattam bin Abdulaziz University, Al Kharj, Saudi Arabia; Department of Health Research (L R Inbaraj MD), ICMR National Institute for Research in Tuberculosis, Chennai, India; Faculty of Health and Life Sciences (A Inok PhD), University of Exeter, Exeter, UK; Faculty of Pharmacy (L M Irham PhD), Universitas Ahmad Dahlan, Yogyakarta, Indonesia; School of Pharmacy (M Islam PhD), BRAC University, Dhaka, Bangladesh; Institute for Physical Activity and Nutrition (Prof S Islam PhD), Deakin University, Burwood, VIC, Australia; Department of Clinical Pharmacy & Pharmacy Practice (Prof N Ismail PhD), Asian Institute of Medicine, Science and Technology, Bedong, Malaysia; Malaysian Academy of Pharmacy, Puchong, Malaysia (Prof N Ismail PhD); Public Health Department of Social Medicine (Prof H Iso MD), Osaka University, Suita, Japan; Department of Medicine (M C Ituka MD), University of Yaoundé, Yaounde, Cameroon; Department of Health Services Research (M Iwagami PhD), Department of Public Health Medicine (Prof K Yamagishi MD), University of Tsukuba, Tsukuba, Japan; Department of Global Health (C J Iwu-Jaja PhD), South African Medical Research Council, Cape Town, South Africa; Department of Global Health (C J Iwu-Jaja PhD), Department of Epidemiology (J L Tamuzi MSc), Stellenbosch University, Cape Town, South Africa; Department of Community Medicine and Family Medicine (V J MD), All India Institute of Medical Sciences, Gorakhpur, India; Department of Physical and Medicine (L Jacob MD), Université Paris Cité, Paris, France; Research and Development Unit (L Jacob MD), Biomedical Research Networking Center for Mental Health Network (CiberSAM), Barcelona, Spain; UCL Institute for Global Health (Prof S Jaffar PhD), University of London, London, UK; College of Medicine and Medical Sciences (H Jahrami PhD), Arabian Gulf University, Manama, Bahrain; Ministry of Health, Manama, Bahrain (H Jahrami PhD); Department of Internal Medicine (A Jain MD), University of Iowa Hospitals and Clinics, Iowa City, IA, USA; Department of Health and Safety (A A Jairoun PhD), Dubai Municipality, Dubai, United Arab Emirates; The World Academy of Sciences UNESCO, Trieste, Italy (Prof M Jakovljevic PhD); Shaanxi University of Technology, Hanzhong, China (Prof M Jakovljevic PhD); Department of Medicine (S Javaid MD), University of Mississippi Medical Center, Jackson, MS, USA; Department of Medicine (S Javaid MD), Jinnah Sindh Medical University, Karachi, Pakistan; Centre of Studies and Research (S Jayapal PhD), Ministry of Health, Muscat, Oman; Postgraduate Institute of Medicine (U Jayarajah MD, S N K Navaratna MD), Department of Anatomy, Genetics and Biomedical Informatics (Y Mathangasinghe PhD), Department of Surgery (D P Wickramasinghe MD), University of Colombo, Colombo, Sri Lanka; Department of Surgery (U Jayarajah MD), National Hospital, Colombo, Sri Lanka; Department of Biochemistry (Prof S Jayaram MD), Government Medical College, Mysuru, India; Department of Epidemiology (R Jebai MPH), Florida International University, Miami, FL, USA; Department of Endocrinology, Diabetes and Metabolism (Prof F K Jebasingh DM), Christian Medical College and Hospital (CMC), Vellore, India; Department of Public Health (A Jema MPH), Madda Walabu University, Goba, Ethiopia; Faculty of Veterinary Medicine (M Jokar DVM), Department of Medicine (Prof M Tonelli MD), University of Calgary, Calgary, AB, Canada; Young Researchers and Elite Club (M Jokar DVM), Islamic Azad University, Karaj, Iran; Rothschild Foundation Hospital (Prof J B Jonas MD), Institut Français de Myopie, Paris, France; Singapore Eye Research Institute (Prof J B Jonas MD), Singapore Eye Research Institute, Singapore, Singapore; Department of Community Medicine (J Jose MD), Jubilee Mission Medical College & Research Institute, Thrissur, Thrissur, India; Department of Economics (C E Joshua

BSc), National Open University, Benin City, Nigeria; Department of Family Medicine and Public Health (J J Jozwiak PhD), University of Opole, Opole, Poland; Institute of Family Medicine and Public Health (M Jürisson PhD), University of Tartu, Tartu, Estonia; School of Public Health (Z Kabir PhD), University College Cork, Cork, Ireland; Department of Pharmacology (A K Kakkar MD), Post Graduate Institute of Medical Education and Research, Chandigarh, India; Department of Endocrinology (S Kalra DM), Bharti Hospital Karnal, Karnal, India; Faculty of Dentistry (K K Kanmodi MPH), University of Puthisastra, Phnom Penh, Cambodia; Office of the Executive Director (K K Kanmodi MPH), Cephas Health Research Initiative Inc, Ibadan, Nigeria; 2nd Cardiology Department (P Karakasis MSc), Second Department of Cardiology (Prof D Patoulias PhD), 2nd Department of Cardiology (P Stachteas MSc), Aristotle University of Thessaloniki, Thessaloniki, Greece; Surgery Research Unit (Prof J H Kauppila MD), University of Oulu, Oulu, Finland; Department of Molecular Medicine and Surgery (Prof J H Kauppila MD), Karolinska Institute, Stockholm, Sweden; International Research Center of Excellence (G A Kayode PhD), Institute of Human Virology Nigeria, Abuja, Nigeria; Department of Surgery (D Kehagias PhD), University of Patras, Patras, Greece; Department of Psychological Medicine (J A Kerr PhD), University of Otago, Christchurch, New Zealand; Department of Human Nutrition (E Kesse-Guyot PhD), National Research Institute for Agriculture, Food and Environment, Jouy-en-Josas, France; Department of Health, Medicine and Human Biology (M Touvier PhD), Sorbonne Paris Nord University, Bobigny, France (E Kesse-Guyot PhD); Department of Public Health and Health Policy (I Khaing MPH), Hiroshima University, Hiroshima, Japan; Amity Institute of Forensic Sciences (H Khajuria PhD, B P Nayak PhD), Amity Institute of Public Health (M Shannawaz PhD), Amity University, Noida, India; Department of Biostatistics (Prof A Khalilian PhD), Department of Medical-Surgical Nursing (S Shorofi PhD), Mazandaran University of Medical Sciences, Sari, Iran; Department of Public Health (Prof M Khalis PhD), Mohammed VI Center for Research and Innovation, Rabat, Morocco; Higher Institute of Nursing Professions and Health Techniques, Rabat, Morocco (Prof M Khalis PhD); Institute of Population Health Sciences (N Khan PhD), Newcastle University, Newcastle Upon Tyne, UK; Department of Community Medicine (M Khan MPH), National Institute of Preventive and Social Medicine, Dhaka, Bangladesh; Natural and Medical Sciences Research Center (A Khan PhD), Dept of Biological Sciences and Chemistry (Z Naureen PhD), University of Nizwa, Nizwa, Oman; Primary Care Department (M A Khan MSc), NHS North West London, London, UK; College of Health, Wellbeing and Life Sciences (Prof K Khatab PhD), Sheffield Hallam University, Sheffield, UK; College of Arts and Sciences (Prof K Khatab PhD), Ohio University, Zanesville, OH, USA; Academy of Medical Science, Tehran, Iran (M Khayamzadeh MD); Department of Biochemistry (F Khidri PhD), Liaquat University Of Medical and Health Sciences, Jamshoro, Pakistan; Department of Epidemiology and Biostatistics (F Khorashadizadeh PhD), Neyshabur University of Medical Sciences, Neyshabur, Iran; Department of Internal Medicine (A A Khosla MD), Corewell Health East William Beaumont University Hospital, Royal Oak, MI, USA; Department of Medical Oncology (A A Khosla MD), Miami Cancer Institute, Miami, FL, USA; Department of Clinical Research (S Khosravi MD), Icahn School of Medicine at Mount Sinai, New York City, NY, USA; Research Department (M Khosrowjerdi PhD), Inland Norway University of Applied Sciences, Elverum, Norway; Department of Public Health (J Khubchandani PhD), New Mexico State University, Las Cruces, NM, USA; Faculty of Health Sciences (H Khusun PhD), University of Muhammadiyah Prof. Dr. Hamka, Jakarta, Indonesia; Program Division (H Khusun PhD), SEAMEO Regional Center for Food and Nutrition, Jakarta, Indonesia; Graduate School of Public Health (K Kim PhD), Kosin University, Busan, South Korea; Broad Institute of MIT and Harvard, Cambridge, MA, USA (M Kim MD); School of Traditional Chinese Medicine (Y Kim PhD), Xiamen University Malaysia, Sepang, Malaysia; Millennium Prevention, Inc., Westwood, MA, USA (R W Kimokoti MD); School of Health

Sciences (Prof A Kisa PhD), Kristiania University College, Oslo, Norway; Department of International Health and Sustainable Development (Prof A Kisa PhD), Tulane University, New Orleans, LA, USA; School of Pharmacy (Prof L Kishore PhD), Maharaja Agrasen University, Himachal Pradesh, India; Department of Public Health (Prof M Kivimäki PhD), University of Helsinki, Helsinki, Finland; Department of General Practice and Family Medicine (Prof O Korzh DSc), Kharkiv National Medical University, Kharkiv, Ukraine; Department of Epidemiology (Prof K Kostev PhD), IQVIA, Frankfurt am Main, Germany; University Hospital Marburg, Marburg, Germany (Prof K Kostev PhD); Kasturba Medical College, Manipal (S Koulmane Laxminarayana MD), Manipal Academy of Higher Education, Udupi, India; School of Pharmacy (Prof I A Kretchy PhD), University of Ghana, Legon, Ghana; Department of Anthropology (Prof K Krishan PhD), Institute of Forensic Science & Criminology (V Sharma PhD), Panjab University, Chandigarh, India; School of Applied Science (C Kua PhD), Republic Polytechnic, Singapore, Singapore; Department of Demography (Prof B Kuate Defo PhD), Department of Social and Preventive Medicine (Prof B Kuate Defo PhD), University of Montreal, Montreal, QC, Canada; Center of Medicine and Public Health (M Kulimbet MSc), Asfendiyarov Kazakh National Medical University, Almaty, Kazakhstan; Department of Medicine (V Kulkarni MS), Digital Health and Informatics Directorate (Prof S M McPhail PhD), Queensland Health, Brisbane, QLD, Australia; Centre for Digital Transformation (V Kumar PhD), Indian Institute of Management, Ahmedabad, Ahmedabad, India; Centre for Studies in Economics and Planning (V Kumar PhD), Central University of Gujarat, Gandhinagar, India; Department of Public Health (S Kundu MPH), School of Medicine and Dentistry (M N Wanjau PhD), Griffith University, Gold Coast, QLD, Australia; Section of Cardiology (Prof S K Kunutsor PhD), University of Manitoba, Winnipeg, MB, Canada; Translational Health Sciences (Prof S K Kunutsor PhD), University of Bristol, Bristol, UK; Department of Medicine (O P Kurmi PhD), Department of Psychiatry and Behavioural Neurosciences (Prof A T Olagunju PhD), Department of Health Research Methods, Evidence, and Impact (O O Olasupo PhD), McMaster University, Hamilton, ON, Canada; Faculty of Medicine and Health Science (M Kurniasari PhD), Universitas Kristen Satya Wacana, Salatiga, Indonesia; Department of Public Health and Epidemiology (D Kusuma DSc), Khalifa University of Science and Technology, Abu Dhabi, United Arab Emirates; Faculty of Public Health (D Kusuma DSc, Prof I Trihandini PhD), Centre for Family Welfare (K Latief PhD), University of Indonesia, Depok, Indonesia; Clinical Research Center (V Kytö MD), Turku University Hospital, Turku, Finland; Nuffield Department of Population Health (B Lacey DPhil), University of Oxford, Oxford, UK; National Institute for Health Research (NIHR) Oxford Biomedical Research Centre, Oxford, UK (B Lacey DPhil); Integrated Department of Epidemiology, Health Policy, Preventive Medicine and Pediatrics (Prof C Lahariya MD), Foundation for People-centric Health Systems, New Delhi, India; Centre for Health: The Specialty Practice, New Delhi, India (Prof C Lahariya MD); School of Digital Science (D Lai PhD), Institute of Applied Data Analytics (D Lai PhD), Universiti Brunei Darussalam (University of Brunei Darussalam), Bandar Seri Begawan, Brunei; Department of Respiratory and Critical Care Medicine (H Lai PhD), Northern Jiangsu People's Hospital, Yangzhou, China; Unidad de Genética y Salud Pública (Prof I Landires MD), Instituto de Ciencias Médicas, Las Tablas, Panama; Ministry of Health (Prof I Landires MD), Hospital Joaquín Pablo Franco Sayas, Las Tablas, Panama; Faculty of Medicine (N Le MD), Department of Internal Medicine (T H Tran MD), University of Medicine and Pharmacy at Ho Chi Minh City, Ho Chi Minh City, Vietnam; Department of Cardiovascular Research (N Le MD), Methodist Hospital, Merrillville, IN, USA; Department of Medical Science (M Lee PhD), Ajou University School of Medicine, Suwon, South Korea; Pattern Recognition and Machine Learning Lab (Prof S Lee PhD), Gachon University, Seongnam, South Korea; Department of Family Medicine (W Lee PhD), University of Texas Medical Branch, Galveston, TX, USA; Department of Precision Medicine (Prof S Lee MD), Sungkyunkwan University, Suwon-si, South

Korea; Southampton Clinical Trials Unit (P H Lee PhD), Department of Surgery (G Verras MSc), University of Southampton, Southampton, UK; Department of Health Promotion and Health Education (M Li PhD), National Taiwan Normal University, Taipei, Taiwan; Department of Endocrinology and Metabolism (Y Li PhD), The First Hospital of China Medical University, Shenyang, China; Population Studies Center (W Li PhD), Department of Biostatistics, Epidemiology, and Informatics (J Puvvula PhD), University of Pennsylvania, Philadelphia, PA, USA; Department of Food Science and Human Nutrition (Q Lin MPH), Iowa State University, Ames, IA, USA; Department of Medical Sciences (D Lindholm MD), Uppsala University, Uppsala, Sweden; Department of Medicine (D Lindholm MD), Norrtälje Hospital (Tiohundra), Norrtälje, Sweden; Departments of Epidemiology and Biostatistics and Medicine (S Liu MD), University of California Irvine, Irvine, CA, USA; Department of Molecular Epidemiology (E Llanaj PhD), German Institute of Human Nutrition Potsdam-Rehbrücke, Potsdam, Germany; German Center for Diabetes Research (DZD), München-Neuherberg, Germany (E Llanaj PhD); One Health Research Group (J López-Gil PhD), Universidad de Las Américas (University of the Americas), Quito, Ecuador; Institute of Nutritional Sciences (Prof S Lorkowski PhD), Friedrich Schiller University Jena, Jena, Germany; Competence Cluster for Nutrition and Cardiovascular Health (nutriCARD), Jena, Germany (Prof S Lorkowski PhD); School of Medicine (Prof G Lucchetti PhD), Federal University of Juiz de Fora, Juiz de Fora, Brazil; Dodoma Medical Research Centre (A M Lutambi PhD), National Institute for Medical Research, Dodoma, Tanzania; Department of Clinical Data Science and Evidence (L Lv PhD), Novo Nordisk, Plainsboro, NJ, USA; Department of Medicine (E Lytvyak MD), University of Alberta, Edmonton, AB, Canada; Centre for Public Health and Wellbeing (Z Ma PhD), University of the West of England, Bristol, UK; Department of Periodontology (Prof M Machoy PhD), Pomeranian Medical University, Szczecin, Poland; Cihan University Sulaimaniya Research Center (CUSRC) (N H Mahmood PhD), Cihan University Sulaimaniya, Sulaymaniyah City, Kurdistan Region, Iraq; Department of Pharmacology (Prof R Maiti MD), All India Institute of Medical Sciences, Bhubaneswar, India; Cyprus International Institute for Environmental and Public Health (Prof K C C Makris PhD), Cyprus University of Technology, Limassol, Cyprus; Rama Medical College Hospital and Research Centre, Uttar Pradesh, India (K Malhotra MBBS); Department of Epidemiology and Biostatistics (Prof M Mansourian PhD, K Mehrabani-Zeinabad PhD), Department of Physiology (H Rezazadeh PhD), Cardiac Rehabilitation Research Center (Prof M Sadeghi MD), Isfahan University of Medical Sciences, Isfahan, Iran; Biomedical Engineering Research Center (CREB) (Prof M Mansourian PhD, H Marateb PhD), Universitat Politècnica de Catalunya (Barcelona Tech - UPC), Barcelona, Spain; Department of Biomedical Engineering (H Marateb PhD, M Noroozi BSc), University of Isfahan, Isfahan, Iran; Department of Food, Environmental and Nutritional Sciences (DeFENS) (M Marino PhD), Department of Food, Environmental and Nutritional Sciences (Prof S Perna PhD), University of Milan, Milano, Italy; Department of Biochemistry (A Marjani PhD), Golestan Research Center of Gastroenterology and Hepatology (G Roshandel PhD), Golestan University of Medical Sciences, Gorgan, Iran; Department of Non-communicable Diseases and Mental Health (R Martinez-Piedra BSc), Pan American Health Organization, Washington, DC, USA; Faculty of Public Health (Prof S Martini PhD), Universitas Airlangga (University of Airlangga), Surabaya, Indonesia; Indonesian Public Health Association, Surabaya, Indonesia (Prof S Martini PhD); Department of Nutrition and Dietetics (M Martorell PhD), Centre for Healthy Living (M Martorell PhD), University of Concepción, Concepción, Chile; Department of Clinical and Experimental Medicine (Prof S Masi PhD), Department of Clinical and Experimental Medicine (D Trico MD), University of Pisa, Pisa, Italy; Institute of Population Health Sciences (M R Mathur PhD), Institute of Population Health (I Putra PhD), University of Liverpool, Liverpool, UK; Faculty of Medical Sciences (Prof T Matthias MD), University of Sri Jayewardenepura, Sri

Lanka, Nugegoda, Sri Lanka; Nuffield Department of Population Health (M Mazidi PhD), University of Oxford, London, UK; Australian Centre for Health Services Innovation (Prof S M McPhail PhD), Queensland University of Technology, Kelvin Grove, QLD, Australia; Department of Healthcare (Prof E A Mechili PhD), University of Vlora, Vlora City, Albania; Clinic of Social and Family Medicine (Prof E A Mechili PhD), Department of Medicine (Prof A Tsatsakis DSc), University of Crete, Heraklion, Greece; National Heart, Lung and Blood Institute (Prof R Mehboob PhD), National Heart, Lung, and Blood Institute, Bethesda, MD, USA; Research and Development Department (Prof R Mehboob PhD), Lahore Medical Research Center, Lahore, Pakistan; Neurology Department (Prof M Mehndiratta MD), Janakpuri Super Specialty Hospital Society, New Delhi, India; Department of Neurology (Prof M Mehndiratta MD), Govind Ballabh Institute of Medical Education and Research, New Delhi, India; Department of Public Health (T Mekene Meto MPH), Arba Minch University, Arba Minch, Ethiopia; Universidad Nacional Mayor de San Marcos, Lima, Peru (W Mendoza MD); University Centre Varazdin (T Mestrovic PhD), University North, Varazdin, Croatia; Department of Paediatrics (Prof S Mettananda DPhil), Department of Pharmacology (Prof C D K Mettananda PhD), University of Kelaniya, Ragama, Sri Lanka; University Paediatrics Unit (Prof S Mettananda DPhil), Clinical Medicine Department (Prof C D K Mettananda PhD), Colombo North Teaching Hospital, Ragama, Sri Lanka; Pacific Institute for Research & Evaluation, Beltsville, MD, USA (T R Miller PhD); Department of Public Health Dentistry (Prof G Mini PhD), Saveetha Institute of Medical and Technical Sciences (SIMATS), Chennai, India; Global Institute of Public Health (Prof G Mini PhD), Ananthapuri Hospitals and Research Institute, Trivandrum, India; Internal Medicine Programme (Prof E M Mirrakhimov PhD), Kyrgyz State Medical Academy, Bishkek, Kyrgyzstan; Department of Atherosclerosis and Coronary Heart Disease (Prof E M Mirrakhimov PhD), National Center of Cardiology and Internal Disease, Bishkek, Kyrgyzstan; National Data Management Center for Health (A Misganaw PhD), Ethiopian Public Health Institute, Addis Ababa, Ethiopia; Department of Endocrinology & Metabolism (Prof M Mittal MD), All India Institute of Medical Sciences, Bhopal, India; College of Health Science (A I Mohamed MSc), College of Applied and Natural Science (J Mohamed MSc), University of Hargeisa, Hargeisa, Somalia; RAK College of Nursing (M Mohamed PhD), RAK Medical and Health Sciences University, Ras Alkhima, United Arab Emirates; Nursing College (M Mohamed PhD), Sohag University, Sohag, Egypt; Molecular Biology Unit (N S Mohamed MSc), Bio-Statistical and Molecular Biology Department (N S Mohamed MSc), Sirius Training and Research Centre, Khartoum, Sudan; Department of Biophysics (T Mohammad PhD), Department of Laboratory Medicine (A Singh PhD), All India Institute of Medical Sciences, New Delhi, India; Health Systems and Policy Research Unit (Prof S Mohammed PhD), Department of Paediatrics (S Musa MSc), Department of Community medicine (U M Umar MPH), Ahmadu Bello University, Zaria, Nigeria; Heidelberg Institute of Global Health (HIGH) (Prof S Mohammed PhD), Department of Ophthalmology (S Panda-Jonas MD), Heidelberg University, Heidelberg, Germany; Department of Biomedical and Dental Sciences and Morphofunctional Imaging (Prof S Mondello MD), Messina University, Messina, Italy; AI & Cyber Futures Institute (M Moni PhD), Charles Sturt University, Bathurst, NSW, Australia; Division of Plastic and Reconstructive Surgery (S D Morrison MD), University of Washington Medical Center, Seattle, WA, USA; Department of Health Policy (Prof E Mossialos PhD), London School of Economics and Political Science, London, UK; Department of Surgery (F Mulita PhD), General University Hospital of Patras, Patras, Greece; Faculty of Medicine (F Mulita PhD), University of Thessaly, Larissa, Greece; Department of Community and Global Health (Y Munkhsaikhan MD), Health Science and Nursing (D W Sari PhD), The University of Tokyo, Tokyo, Japan; Clinical Epidemiology Research Unit (E Murillo-Zamora PhD), Mexican Institute of Social Security, Villa de Alvarez, Mexico; Postgraduate in Medical Sciences (E Murillo-Zamora PhD), Universidad de Colima,

Colima, Mexico; College of Medicine (Prof G Mustafa MD), Shaqra University, Riyadh, Shaqra, Saudi Arabia; Department of Pediatrics & Pediatric Pulmonology (Prof G Mustafa MD), Institute of Mother & Child Care, Multan, Pakistan; Department of Research Methods (S Muthu PhD), Orthopaedic Research Group, Coimbatore, India; Department of Biotechnology (S Muthu PhD), Karpagam Academy of Higher Education (Deemed to be University), Coimbatore, India; Department of Psychiatry (W Myung PhD), Department of Food and Nutrition (A P Okekunle PhD), Seoul National University, Seoul, South Korea; Department of Neuropsychiatry (W Myung PhD), Seoul National University Bundang Hospital, Seongnam, South Korea; Department of Computer Science (P Naghavi MS), University of Illinois, Urbana, IL, USA; Department of Medicine (H Naik MS), School of Nursing (A Pashaei MSc), University of British Columbia, Vancouver, BC, Canada; Suraj Eye Institute, Nagpur, India (V Nangia MD); Department for the Control of Disease, Epidemics, and Pandemics (J Nansseu MD), Ministry of Public Health, Yaoundé, Cameroon; Department of Public Heath (J Nansseu MD), University of Yaoundé I, Yaoundé, Cameroon; National Dental Research Institute Singapore (G G Nascimento PhD), Duke-NUS Medical School, Singapore, Singapore; Department of Medicine (M Nassar PhD), University at Buffalo, Buffalo, NY, USA; Department of Internal Medicine (M Nassar PhD), Mount Sinai Health System, Queens, NY, USA; Department of Circulation and Medical Imaging (J Nauman PhD), Norwegian University of Science and Technology, Trondheim, Norway; Zilber College of Public Health (M Nayon MPH), University of Wisconsin Milwaukee, Milwaukee, WI, USA; Department of Health Promotion (A Nazri-Panjaki MSc), Health Promotion Research Center (H Okati-Aliabad PhD), Zahedan University of Medical Sciences, Zahedan, Iran; Department of Cardiology (R I Negoi PhD), Cardio-Aid, Bucharest, Romania; Department of General Surgery (I Negoi PhD), Emergency University Hospital Bucharest, Bucharest, Romania; HIV/STI Surveillance Research Center (S Nejadghaderi MD), Kerman University of Medical Sciences, Kerman, Iran; Department of Biological Sciences (J W Ngunjiri PhD), University of Embu, Embu, Kenya; Institute for Global Health Innovations (C T Nguyen MPH), Duy Tan University, Hanoi, Vietnam; Harvard T.H. Chan School of Public Health (D Nguyen BS), Harvard University, Cambridge, MA, USA; Department of Medical Engineering (D Nguyen BS), University of South Florida, Tampa, FL, USA; Faculty of Public Health (T Nguyen PhD), Vietnam National University, Hanoi, Vietnam; Faculty of Medicine (T Nguyen PhD), Duy Tan University, Da Nang, Vietnam; Cardiovascular Laboratory (D H Nguyen MD), Methodist Hospital, Merrillville, Merrillville, IN, USA; Hitotsubashi Institute for Advanced Study (HIAS) (P T Nguyen DrPH), Hitotsubashi University, Tokyo, Japan; Institute for Cancer Control (P T Nguyen DrPH), National Cancer Center, Chuo-ku, Japan; International Islamic University Islamabad, Islamabad, Pakistan (R K Niazi PhD); Department of Humanities and Social Science (L Nieddu PhD), University for International Studies in Rome (UNINT), Rome, Italy; Department of Internal Medicine and Specialties (J Nkeck MD), University of Yaoundé I, Yaounde, Cameroon; Global Research Institute (Prof S Nomura PhD), Keio University, Tokyo, Japan; Department of Statistics (S Noor MS), Shahjalal University of Science and Technology, Sylhet, Bangladesh; Department of Microbiology and Molecular Genetics (M Noreen PhD), The Women University Multan, Multan, Pakistan; School of Medicine (Nawsherwan PhD), Xiamen University, Xiamen, China; Division of Cardiology (J Noubiap MD), Department of Neurosurgery (Y Senol MD), Department of Bioengineering and Therapeutical Sciences (Prof M Zastrozhin PhD), University of California San Francisco, San Francisco, CA, USA; Health Research Institute (M Nouri PhD), School of Medicine (S Soraneh MD), Babol University of Medical Sciences, Babol, Iran; Department of Paediatrics (C A Nri-Ezedi PhD), Nnamdi Azikiwe University, Awka, Nigeria; School of Information (F Nugen PhD), Health Initiative of the Americas (Prof V E Villalobos-Daniel PhD), University of California Berkeley, Berkeley, CA, USA; Department of Public Health (D Nurrika PhD), Banten School of Health Science, South

Tangerang, Indonesia; Ministry of Research, Technology and Higher Education (D Nurrika PhD), Higher Education Service Institutions (LL-DIKTI) Region IV, Bandung, Indonesia; Department of Physiology (O J Nzoputam PhD), University of Benin, Edo, Nigeria; Department of Physiology (O J Nzoputam PhD), Benson Idahosa University, Benin City, Nigeria; Department of Applied Economics and Quantitative Analysis (Prof B Oancea PhD), University of Bucharest, Bucharest, Romania; Bioinformatics Department (Prof B Oancea PhD), National Institute of Research and Development for Biological Sciences, Bucharest, Romania; Sheffield Centre for Health and Related Research (J O Oguta MSc), University of Sheffield, Sheffield, UK; Department of Preventive Medicine (Prof I Oh MD), Department of Pediatrics (Prof D Yon MD), Kyung Hee University, Seoul, South Korea; School of Pharmacy (O C Okonji MSc), University of the Western Cape, Cape Town, South Africa; Department of Psychiatry (Prof A T Olagunju PhD), University of Lagos, Lagos, Nigeria; Division of Nephrology, Department of Medicine (Prof T O Olanrewaju MSc), University of Ilorin, Ilorin, Nigeria; Cardiology Department (Prof G M M Oliveira PhD), Federal University of Rio de Janeiro, Rio de Janeiro, Brazil; Associação Brasileira de Cefaleia em Salvas e Enxaqueca (ABRACES), São Paulo, Brazil (A B Oliveira PhD); Department of Pharmacology and Toxicology (Prof H A Omar PhD), Beni-Suef University, Beni-Suef, Egypt; Diplomacy and Public Relations Department (A Omar Bali PhD), University of Human Development, Sulaymaniyah, Iraq; Institute of Diagnostic and Interventional Radiology and Neuroradiology (M Opitz MD), University Hospital Essen, Essen, Germany; Department of Pharmacotherapy and Pharmaceutical Care (M Ordak PhD), Department of Biochemistry and Pharmacogenomics (M Zielińska MPharm), Medical University of Warsaw, Warsaw, Poland; Department of Medicine (Prof A Ortiz MD), Autonomous University of Madrid (Universidad Autónoma de Madrid), Madrid, Spain; Department of Nephrology and Hypertension (Prof A Ortiz MD), The Institute for Health Research Foundation Jiménez Díaz University Hospital, Madrid, Spain; Department of Biological Sciences (A Osborne MSc), Njala University, Sierra Leone, Freetown, Sierra Leone; Department of Biology (W M S Osman PhD), Khalifa University, Abu Dhabi, United Arab Emirates; Department of Clinical Pharmacy and Pharmacy Practice (A A M Osman MSc), University of Gezira, Wad Madani, Sudan; School of Medicine (U L Osuagwu PhD), Western Sydney University, Bathurst, NSW, Australia; Department of Optometry and Vision Science (U L Osuagwu PhD), University of KwaZulu-Natal, KwaZulu-Natal, South Africa; Department of Statistics and Econometrics (A Otoiu PhD, I Petcu PhD), Bucharest University of Economic Studies, Bucharest, Romania; Department of Public Health (A Oumer PhD), Dire Dawa University, Dire Dawa, Ethiopia; Division of Infectious Diseases (Prof A Ouyahia PhD), University Hospital of Setif, Setif, Algeria; West African Center for Cell Biology of Infectious Pathogens (I A Owusu PhD), University of Ghana, Legon-Accra, Ghana; Department of Respiratory Medicine (Prof M P P A DNB), JSS Medical College (Prof P K Santhekadur PhD), Jagadguru Sri Shivarathreeswara University, Mysore, India; National School of Public Health (A Padron-Monedero PhD), Institute of Health Carlos III, Madrid, Spain; Centre for Biotechnology (S K Panda PhD), Siksha 'O' Anusandhan (Deemed to be University), Bhubaneswar, India; Centre for Research and Development (Prof S R Pandi-Perumal MSc), Chandigarh University, Punjab, India; Division of Research and Development (Prof S R Pandi-Perumal MSc), Lovely Professional University, Phagwara, India; Vision and Eye Research Institute (Prof S Pardhan PhD), Anglia Ruskin University, Cambridge, UK; Department of Forensic Medicine and Toxicology (U Parekh MD), All India Institute of Medical Sciences, Rajkot, India; Department of Community Medicine (P P Parija MD), All India Institute of Medical Sciences, Jammu, India; Division of Health Policy and Management (R R Parikh MD), University of Minnesota, Minneapolis, MN, USA; Department of Preventive Medicine (Prof E Park PhD), Institute of Health Services Research (Prof E Park PhD), Yonsei University, Seoul, South Korea; Department of Medical Sciences (R Passera PhD), University of Torino,

Torino, Italy; Department of Imaging (R Passera PhD), AOU Città della Salute e della Scienza di Torino, Torino, Italy; Department of Physiotherapy (H M Patel PhD), Ashok & Rita Patel Institute of Physiotherapy (S Sunny PhD), Charotar University of Science and Technology, Anand, India; Research Consultancy (A R Pathan PhD), Author Gate Publications, Malegaon, India; Department of Biomedical Sciences (U Pensato MD), Humanitas University, Pieve Emanuele (MI), Italy; School of Population Health (Prof G Pereira PhD), Curtin University, Bentley, WA, Australia; Centre for Fertility and Health (Prof G Pereira PhD), Department of Chemical Toxicology (M W Wojewodzic PhD), Norwegian Institute of Public Health, Oslo, Norway; Institute of Collective Health (Prof M Pereira PhD), Federal University of Bahia, Salvador, Brazil; Mario Negri Institute for Pharmacological Research, Bergamo, Italy (N Perico MD, Prof G Remuzzi MD); Facultad de Medicina (Faculty of Medicine) (F E Petermann-Rocha PhD), Universidad Diego Portales (Diego Portales University), Santiago, Chile; School of Cardiovascular and Metabolic Health (F E Petermann-Rocha PhD), University of Glasgow, Glasgow, UK; Department of Internal Medicine (H Pham MD), University of Arizona, Tucson, AZ, USA; Department of Internal Medicine (D S Popovic PhD), University of Novi Sad, Novi Sad, Serbia; Clinic for Endocrinology, Diabetes and Metabolic Disorders (D S Popovic PhD), Clinical Center of Vojvodina, Novi Sad, Serbia; Department of Humanities and Social Sciences (Prof J Pradhan PhD), National Institute of Technology Rourkela, Rourkela, India; Department of Community Medicine and Public Health (P M S Pradhan MD), Tribhuvan University, Kathmandu, Nepal; Department of Clinical Research and Epidemiology (M Prasad MD), Institute of Liver and Biliary Sciences, New Delhi, India; Department of Medical Oncology (Prof V Radhakrishnan MD), Cancer Institute (W.I.A), Chennai, India; Institute of Health and Wellbeing (Prof M Rahman PhD), Federation University Australia, Berwick, VIC, Australia; School of Nursing and Midwifery (Prof M Rahman PhD), La Trobe University, Melbourne, VIC, Australia; College of Health Sciences (F M Rahman PhD), Cihan University - Sulaimaniya, Sulaymaniyah, Iraq; College of Medicine and Health Sciences (M Rahman PhD), National University of Science and Technology, Sohar, Oman; Department of Radiology and Biomedical Imaging (S Rahmani MD), Department of Psychiatry (T Rhee PhD), Yale University, New Haven, CT, USA; Department of Public Health (V Rahmanian PhD), Torbat Jam Faculty of Medical Sciences, Torbat Jam, Iran; Department of Nutrition Science (S Rahmawaty PhD), Muhammadiyah University of Surakarta, Surakarta, Indonesia; Society for Health and Demographic Surveillance, Suri, India (R Rai PhD); Institute of Nutrition (R Rai PhD), Mahidol University, Salaya, Thailand; Department of Medical, Surgical and Experimental Sciences (I Raimondo MD), University of Sassari, Sassari, Italy; Gynecology and Breast Care Center (I Raimondo MD), Mater Olbia Hospital, Olbia, Italy; Centre for Chronic Disease Control, New Delhi, India (P Rajput PhD); Department of Anatomy (C Ramasamy MD), Govt. Siddhartha Medical College, Vijayawada, India; Department of Radiology (S Ramasamy MD), Stanford University, Stanford, CA, USA; School of Nursing & Health Sciences (S Ramazanu PhD), Hong Kong Metropolitan University, Hong Kong, China; Department of Research (C L Ranabhat PhD), Eastern Scientific LLC, Richmond, KY, USA; Planetary Health Research Centre (PHRC), Kathmandu, Nepal (C L Ranabhat PhD); Department of Oral Pathology, Microbiology and Forensic Odontology (S Rao MDS), Sharavathi Dental College and Hospital, Shimogga, India; Brigham and Women's Hospital (S Rashedi MD), Harvard Medical School, Boston, MA, USA; Department of Neurosurgery (I Rautalin PhD), Helsinki University Hospital, Helsinki, Finland; The National Institute for Stroke and Applied Neurosciences (I Rautalin PhD), Auckland University of Technology, Auckland, New Zealand; Inovus Medical, St Helens, UK (D L Rawaf MD); Academic Public Health England (Prof S Rawaf MD), Public Health England, London, UK; Department of Biological Sciences (Prof E M M Redwan PhD), King Abdulaziz University, Jeddah, Egypt; Department of Protein Research (Prof E M M Redwan PhD), Research and Academic Institution,

Alexandria, Egypt; Institute for Health, Health Care Policy and Aging Research (S Rege PhD), Rutgers University, New Brunswick, NJ, USA; Department of Epidemiology and Biostatistics (Prof M Rezaeian PhD), Rafsanjan University of Medical Sciences, Rafsanjan, Iran (H Rezazadeh PhD); Department of Public Health Sciences (T Rhee PhD), University of Connecticut, Farmington, CT, USA; Department of Geography and Demography (M Rodrigues PhD), University of Coimbra, Coimbra, Portugal; Department of Nursing in Women's Health (T Rodrigues da Silva PhD), Federal University of São Paulo, São Paulo, Brazil; Department of Pharmacology and Toxicology (Prof J A B Rodriguez PhD), University of Antioquia, Medellin, Colombia; Warwick Medical School (Prof J A B Rodriguez PhD), University of Warwick, Coventry, UK; Department of Clinical Research (Prof L Roever PhD), University of Sao Paulo, Ribeirão Preto, Brazil; Center for Indigenous Health Research (P Rohloff MD), Wuqu' Kawoq Maya Health Alliance, Tecpan, Guatemala; Department of Public Health (M Rony MPH), Bangladesh Open University, Gazipur, Bangladesh; Department of Analytical and Applied Economics (Prof H Rout PhD, C Swain MPhil), RUSA Centre of Excellence in Public Policy and Governance (Prof H Rout PhD), Utkal University, Bhubaneswar, India; Department of Biochemistry and Food Analysis (N Roy PhD), Patuakhali Science and Technology University, Patuakhali, Bangladesh; Department of Internal Medicine (G M Rwegerera MD), University of Botswana, Gaborone, Botswana; Department of Medical Pharmacology (Prof M M Saber-Ayad PhD), Public Health and Community Medicine Department (M R Salem MD), Cairo University, Giza, Egypt; Faculty of Health and Dentistry (Prof K P Sadarangani PhD), Diego Portales University, Santiago de Chile, Chile; Autonomous University of Chile, Santiago de Chile, Chile (Prof K P Sadarangani PhD); Department of Pharmaceutical Chemistry (Prof M Saeb PhD), International Medical University, Gdańsk, Poland; Operational Research Center in Healthcare (Prof U Saeed PhD), Near East University (NEU), Nicosia Cyprus, Turkiye; International Center of Medical Sciences Research (ICMSR), Islamabad, Pakistan (Prof U Saeed PhD); Kiel Institute for the World Economy, Kiel, Germany (S Saeedi Moghaddam MSc); Faculty of Medicine, Bioscience and Nursing (S Safi PhD), MAHSA University, Selangor, Malaysia; Interdisciplinary Research Centre in Biomedical Materials (IRCBM) (S Safi PhD), COMSATS Institute of Information Technology, Lahore, Pakistan; Biotechnology Research Center (Prof A Sahebkar PhD), Faculty of Medicine (F Shahrahmani MD), Department of Medical Informatics (S Tabatabaei PhD), Clinial Research Development Unit (S Tabatabaei PhD), Department of Medicine (A Yarahmadi PhD), Mashhad University of Medical Sciences, Mashhad, Iran; Department of Community Medicine and Family Medicine (S S Sahoo MD, M Verma MD), All India Institute of Medical Sciences, Bathinda, India; Department of Statistics (M R Sajid PhD), University of Gujrat, Gujrat, Pakistan; Department of Integrated Health Education (Prof L B Salaroli PhD), Federal University of Espirito Santo, Vitória, Brazil; Benang Merah Research Center (BMRC), Minahasa Utara, Indonesia (Y L Samodra PhD); Department of Anatomy (Prof V P Samuel PhD), Ras Al Khaimah Medical and Health Sciences University, Ras Al Khaimah, United Arab Emirates; Research Development Coordination Section (M N Saqib PhD), Pakistan Health Research Council, Islamabad, Pakistan; Indira Gandhi Medical College and Research Institute, Puducherry, India (A Saravanan MD); Faculty of nursing (D W Sari PhD), Universitas Airlangga (Airlangga University), Kab. Sidoarjo, Indonesia; Department of Food Processing Technology (T Sarkar PhD), West Bengal State Council of Technical Education, Malda, India; Department of Environmental Health Engineering (M Sarmadi MSc), Health Sciences Research Center (M Sarmadi MSc), Torbat Heydariyeh University of Medical Sciences, Torbat Heydariyeh, Iran; Department of Oral Pathology and Microbiology (Prof S C Sarode PhD, Prof G S Sarode PhD), Dr. D. Y. Patil Vidyapeeth, Pune (Deemed to be University), Pune, India; Faculty of Health & Social Sciences (B Sathian PhD), Bournemouth University, Bournemouth, UK; Department of Preventive and Social Medicine (G Saya MD), Jawaharlal Institute of Postgraduate

Medical Education and Research, Puducherry, India; Faculty of Business and Computing (Prof C Schinckus PhD), University of the Fraser Valley, Abbotsford, BC, Canada; Department of Finance (Prof C Schinckus PhD), International School of Management, Paris, France; Cardiovascular Program (X Xu PhD), The George Institute for Global Health, Sydney, NSW, Australia (Prof A E Schutte PhD); Alberta Health Services, Calgary, Canada (S Sebastian MD); Dr. D. Y. Patil Dental College and Hospital (S Selvaraj PhD), Dr. D. Y. Patil Vidyapeeth, Pune, India; Department of Biomedical Sciences (P Sengupta PhD), Gulf Medical University, Ajman, United Arab Emirates; Emergency Department (S Senthilkumaran PhD), Manian Medical Centre, Erode, India; Department of Medicine and Surgery (Y Sethi M.B.B.S.), Government Doon Medical College, Dehradun, India; National Heart, Lung, and Blood Institute (A Seylani BS), National Institutes of Health, Rockville, MD, USA; Rita A Patel Institute of Physiotherapy (S Shah PhD), The Charutar Vidya Mandal (CVM) University, Anand, India; Department of Family Medicine (Prof S M Shah PhD), Aga Khan University, Karachi, Pakistan; Department of Pathology and Laboratory Medicine (S Sham MD), Northwell Health, New York, NY, USA; School of Medicine (M Shams-Beyranvand MSc), Alborz University of Medical Sciences, Karaj, Iran; National University of Ireland - Galway, Galway, Ireland (D Shan MD); Columbia University, New York, NY, USA (D Shan MD); Department of Medicine (M Sharath MBBS), Bangalore Medical College and Research Institute, Bangalore, India; Department of Safety Services (S Sharfaei MD), Baim Institute for Clinical Research, Boston, MA, USA; Department for Evidence-based Medicine and Evaluation (A Sharifan PharmD), University for Continuing Education Krems, Krems, Austria; Department of Hemato-oncology (A Sharma MD), Fortis Hospital, Noida, India; Department of Social and Behavioral Health (Prof M Sharma PhD), University of Nevada Las Vegas, Las Vegas, NV, USA; Tokyo Foundation for Policy Research, Tokyo, Japan (Prof K Shibuya MD); Department of Public Health (D Shiferaw MPH), Dambi Dollo University, Dembi Dollo, Ethiopia; Finnish Institute of Occupational Health, Helsinki, Finland (R Shiri PhD); Department of Public Health (R Shrestha MPH), Nepal Development Society, Pokhara, Nepal; Research Unit for Global Health (R Shrestha MPH), Aarhus University, Aarhus, Denmark; Kenneth H. Cooper Institute (Prof K Shuval PhD), Texas Tech University Health Sciences Center, Dallas, TX, USA; Advanced Materials Division (N R S Sibuyi PhD), Mintek, Randburg, South Africa; Department of Biotechnology (N R S Sibuyi PhD), University of the Western Cape, Bellville, South Africa; Department of Medical Microbiology and Infectious Diseases (E E Siddig MD), Erasmus University, Rotterdam, Netherlands; Department of Cardiothoracic Imaging (A Siddiqi MD), Emory University, Atlanta, GA, USA; Anthropological Survey of India (M Sikdar PhD), Anthropological Survey of India, Mysore, Karnataka, India; CICS-UBI Health Sciences Research Center (Prof L M R Silva PhD), University of Beira Interior, Covilhã, Portugal; School of Medicine (Prof J A Singh MD), Baylor College of Medicine, Houston, TX, USA; Department of Medicine Service (Prof J A Singh MD), US Department of Veterans Affairs (VA), Houston, TX, USA; Department of Pharmacology (H Singh DM), Government Medical College and Hospital, Chandigarh, India; Department of Human Genetics (P Singh PhD), Punjabi University, Patiala, India; Books Committee (V Y Skryabin MD), Royal College of Psychiatrists, London, UK (V Y Skryabin MD); Department of Infectious Diseases and Epidemiology (A A Skryabina MD), Department of Internal Disease (A V Starodubova DSc), Pirogov Russian National Research Medical University, Moscow, Russia; Student Research Committee (S Soraneh MD), Urmia University of Medical Sciences, Urmia, Iran; 3rd Department of Cardiology (M Spartalis PhD), University of Athens, Athens, Greece; Nutrition and Dietetics Department (A V Starodubova DSc), Federal Research Institute of Nutrition, Biotechnology and Food Safety, Moscow, Russia; Global Observatory on Pollution and Health (Prof K Straif PhD), Boston College, Chestnut Hill, MA, USA; ISGlobal Instituto de Salud Global de Barcelona, Barcelona, Spain (Prof K Straif PhD); Department of Medical Sciences (Prof V

Subramaniyan PhD), Sunway University, Subang Jaya, Malaysia; Department of Human Anatomy (M O Suleiman Odidi PhD), Federal University, Dutse, Dutse, Nigeria; GKT School of Medical Education (A Sulkowski BSc), School of Life Course and Population Sciences (Prof Y Wang PhD), King's College London, London, UK; School of Medicine, Medical Sciences and Nutrition (A Sultan Meo MPH), University of Aberdeen, Aberdeen, UK; Institute of Integrated Intelligence and Systems (Prof J Sun PhD), Griffith University, Brisbane, QLD, Australia; Department of Biomedical Sciences (Z Sun PhD), Universiti Putra Malaysia, Selangor, Malaysia; Department of Clinical Research and Development (Prof L Szarpak PhD), LUXMED Group, Warsaw, Poland; Collegium Medicul (Prof L Szarpak PhD), John Paul II Catholic University of Lublin, Lublin, Poland; Department of Medicine (Prof R Tabarés-Seisdedos PhD), University of Valencia, Valencia, Spain; Department of Environmental, Agricultural and Occupational Health (J Taiba PhD), University of Nebraska Medical Center, Omaha, NE, USA; Sri Ramachandra Medical College and Research Institute, Chennai, India (J Taiba PhD); Department of Dermato-Venereology (M Tampa PhD), Dr. Victor Babes Clinical Hospital of Infectious Diseases and Tropical Diseases, Bucharest, Romania; Department of Medicine (J L Tamuzi MSc), Northlands Medical Group, Omuthiya, Namibia; Department of Radiology (M Tanwar MD), University of Alabama at Birmingham, Birmingham, AL, USA; Department of Pharmacology and Therapeutics (S Tariq PhD), The University of Faisalabad, Faisalabad, Pakistan; Taking Our Best Shot, Houston, TX, USA (N Y Tat MS); Department of Research and Innovation (N Y Tat MS), Enventure Medical Innovation, Houston, TX, USA; Outpatient Department (D R Terefa MSc), Wollega University, Bedele Town, Ethiopia; Faculty of Public Health (J H V Ticoalu MPH), Universitas Sam Ratulangi (Sam Ratulangi University), Manado, Indonesia; Public Health Department (T Y Tiruye PhD), Department of Public Health (T E Wonde MPH), Debre Markos University, Debre Markos, Ethiopia; Laboratory of Public Health Indicators Analysis and Health Digitalization (M V Titova PhD), Moscow Institute of Physics and Technology, Moscow, Russia; Nutritional Epidemiology Research Team (EREN) (M Touvier PhD), National Institute for Health and Medical Research (INSERM), Paris, France; High Institute of Sport and Physical Education of Sfax (K Trabelsi PhD), University of Sfax, Sfax, Tunisia; School of Medicine and Dentistry (M T N Tran PhD), Queensland University of Technology, GoldCoast, QLD, Australia; Health Informatics Department (M T N Tran PhD), Hanoi Medical University, Ha Noi, Vietnam; Department of Business Analytics (T H Tran MD), University of Massachusetts Dartmouth, Dartmouth, MA, USA; Molecular Neuroscience Research Center (N Tran Minh Duc MD), Shiga University of Medical Science, Shiga, Japan; Department of Cardiology (Prof G Tse PhD), Tianjin Medical University, Tianjin, China; Kent and Medway Medical School, Canterbury, UK (Prof G Tse PhD); Department of Internal Medicine (M Tumurkhuu PhD), Wake Forest University, Winston-Salem, NC, USA; Department of Nutrition and Food Studies (S Tyrovolas PhD), George Mason University, Fairfax, VA, USA; International Center for Chemical and Biological Sciences (S Ullah MSc), University of Karachi, Karachi, Pakistan; Federal University of Health Sciences Azare (L Umar PhD), Federal Teaching Hospital, Azare, Bauchi-State, Nigeria; Federal Teaching Hospital Azare (L Umar PhD), Federal Medical Centre, Azare, Nigeria; Department of Cardiovascular, Endocrine-metabolic Diseases and Aging (B Unim PhD), National Institute of Health, Rome, Italy; Amity Institute of Biotechnology (E Upadhyay PhD), Amity University Rajasthan, Jaipur, India; College of Sciences (D Ustunsoz BS), Louisiana State University and A&M College, Baton Rouge, LA, USA; UKK Institute, Tampere, Finland (Prof T J Vasankari PhD); Faculty of Medicine and Health Technology (Prof T J Vasankari PhD), Tampere University, Tampere, Finland; Department of Infectious Disease (Prof S Vaziri MD), Kermanshah University of Medical Sciences, Kermanshah, Iran; Department of Human Genetics & Molecular Biology (B Vellingiri PhD), Bharathiar University, Coimbatore, India; Raffles Neuroscience Centre (Prof N Venketasubramanian MSc), Raffles Hospital,

Singapore, Singapore; Department of Internal Medicine (A Verma MD), Texas Tech University, Odessa, TX, USA; Department of Neurology (S Vidale MD), Infermi Hospital, Rimini, Italy; Department of Neurology & Stroke Unit (S Vidale MD), Sant'Anna Hospital, Como, Italy; Center for Disease and Control Programs (Prof V E Villalobos-Daniel PhD), Ministry of Health, Mexico City, Mexico; Department of Cardiology (M Vinayak MD), Icahn School of Medicine at Mount Sinai, New York, NY, USA; Department of Health Care Administration and Economics (Prof V Vlassov MD), National Research University Higher School of Economics, Moscow, Russia; Department of Pediatric Endocrinology (R Vukovic PhD), Mother and Child Healthcare Institute of Serbia "Dr Vukan Cupic", Belgrade, Serbia; Research Organization for Health (M Wahidin PhD), National Research and Innovation Agency, Jakarta, Indonesia; Faculty of Public Health (M Wahiduzzaman PhD), Bangladesh University of Health Sciences (BUHS), Dhaka, Bangladesh; Department of Cardiology (M Wahiduzzaman PhD), National Healthcare Network-Diabetic Association of Bangladesh, Dhaka, Bangladesh; Department of Neurosurgery (S Wang MD), Capital Medical University, Beijing, China; Department of Neurosurgery (S Wang MD), Beijing Tiantan Hospital, Beijing, China; Brigham and Women's Hospital, Boston, MA, USA (C Wang PhD); Shandong University of Traditional Chinese Medicine (X Wang MD), Shandong University of Traditional Chinese Medicine, Jinan, China; School of Nursing Sciences (M N Wanjau PhD), University of Nairobi, Nairobi, Kenya; Key Laboratory of Computer-Aided Drug Design (M Waqas PhD), Guangdong Medical University, Dongguan, China; Department of Biotechnology and Genetic Engineering (M Waqas PhD), Hazara University Mansehra, Mansehra, Pakistan; Department of Orthopaedics (F Wei PhD), General Hospital of Central Theater Command, Wuhan, China; Fourth Military Medical University, Xi'an, China (F Wei PhD); Department of Medical Surgical Nursing (A L Wicaksana MS), Gadjah Mada University, Yogyakarta, Indonesia; Institute of Clinical Epidemiology, Public Health, Health Economics, Medical Statistics and Informatics (Prof P Willeit PhD), Medical University Innsbruck, Innsbruck, Austria; Faculty of Public Health (U Wongsin PhD), Mahidol University, Bangkok, Thailand; Australian Centre for Health Services Innovation (Q Xia PhD), Queensland University of Technology, Brisbane, QLD, Australia; Department of Intelligent Medical Engineering (Prof W Xie DrPH), Anhui Medical University, Anhui, China; Department of Endocrinology (Prof S Xu PhD), University of Science and Technology of China, Hefei, China; School of Medicine (Prof S Xu PhD), University of Rochester, Rochester, NY, USA; Department of Public Health (Prof K Yamagishi MD, Prof N Yonemoto PhD), Faculty of Medicine (Y Yano MD), Juntendo University, Tokyo, Japan; School of Traditional Chinese Medicine (Prof H Yao PhD), Beijing University of Chinese Medicine, Beijing, China; Pritzker School of Medicine (Prof H Yao PhD), University of Chicago, Chicago, IL, USA; Research Center of Physiology (H Yaribeygi PhD), Semnan University of Medical Sciences, Semnan, Iran; Department of Family Medicine (S A Yesuf MSc), St. Paul's Hospital Millennium Medical College, Addis Ababa, Ethiopia; Independent Consultant, Addis Ababa, Ethiopia (S A Yesuf MSc); Department of Epidemiology (D Yin DrPH), Xuzhou Medical University, Xuzhou, China; Department of Biostatistics (Prof N Yonemoto PhD), University of Toyama, Toyama, Japan; Department of Epidemiology and Biostatistics (Prof C Yu PhD), Wuhan University, Wuhan, China; Cancer Institute (D Yuce MD), Hacettepe University, Ankara, Turkiye; Department of Clinical Pharmacy and Outcomes Sciences (I Yunusa PhD), University of South Carolina, Columbia, SC, USA; Department of Health Sciences (S Zaman PhD), James Madison University, Harrisonburg, VA, USA; Research and Development Department (I Zare BSc), Sina Medical Biochemistry Technologies, Shiraz, Iran; Department of Administration (Prof M Zastrozhin PhD), PGxAI, San Francisco, CA, USA; Department of Public Health (M G M Zeariya PhD), University of Hail, Hail, Saudi Arabia; Department of Zoology and Entomology (M G M Zeariya PhD), Al-Azhar University, Cairo, Egypt; Department of Internal Medicine (X Zhang MD), Graduate Medical Education (X Zhang MD), Albert

Einstein College of Medicine, Bronx, NY, USA; Medical Oncology Department of Gastrointestinal Cancer (L Zhang MS), Cancer Hospital of Dalian University of Technology, Shenyang, China; School of Biomedical Engineering (L Zhang MS), Dalian University of Technology, Dalian, China; School of Public Policy and Administration (J Zhang BA), Xi 'an Jiaotong University, Xi'an, China; Tianjin Medical University General Hospital (Z Zhang MD), Tianjin Centers for Disease Control and Prevention, Tianjin, China; School of Public Health (C J P Zhang PhD), University of Hong Kong, Hong Kong SAR, China; School of Public Health and Emergency Management (B Zhu PhD), Southern University of Science and Technology, Shenzhen, China; Endocrinology and Metabolism Research Center (G Zoghi MD), Hormozgan University of Medical Sciences, Bandar Abbas, Iran; Institute of Child and Adolescent Health (Prof Z Zou MD), Peking University, Beijing, China; Department of Cardiology, Pulmonology, and Vascular Medicine (E Zweck MD), Heinrich-Heine-University, Duesseldorf, Germany; Clinical Research Centre (Prof S H Zyoud PhD), An-Najah National University Hospital, Nablus, Palestine; GBD Collaborating Unit (Prof S E Vollset DrPH), Norwegian Institute of Public Health, Bergen, Norway

Authors' Contributions

Managing the overall research enterprise

Xiaochen Dai, Emmanuela Gakidou, Simon I Hay, Stephen S Lim, Paulina A Lindstedt, Erin Mullany, Christopher J L Murray, Erin O'Connell, and Amanda E Smith.

Writing the first draft of the manuscript

Peter Azzopardi, Karly I Cini, Rebecca M Cogen, Xiaochen Dai, Dorothea Dumuid, Kate Louise Francis, Emmanuela Gakidou, Jessica A Kerr, Marie Ng, and Susan M Sawyer

Primary responsibility for applying analytical methods to produce estimates

Dana Bryazka, Rebecca M Cogen, and Xiaochen Dai

Primary responsibility for seeking, cataloguing, extracting, or cleaning data; designing or coding figures and tables

Noah Ahmad, Rebecca M Cogen, and Xiaochen Dai

Providing data or critical feedback on data sources

Yohannes Habtegiorgis Abate, Cristiana Abbafati, Samar Abd ElHafeez, Rizwan Suliankatchi Abdulkader, Auwal Abdullahi, Armita Abedi, Richard Gyan Aboagye, Shady Abohashem, Bilyaminu Abubakar, Hana J Abukhadijah, Niveen ME Abu-Rmeileh, Ahmed Abu-Zaid, Mesafint Molla Adane, Kamoru Ademola Adedokun, Usha Adiga, Qorinah Estiningtyas Sakilah Adnani, Muhammad Sohail Afzal, Saira Afzal, Danish Ahmad, Noah Ahmad, Sajjad Ahmad, Ayman Ahmed, Haroon Ahmed, Mehrunnisha Sharif Ahmed, Meqdad Saleh Ahmed, Muktar Beshir Ahmed, Hanadi Al Hamad, Zain Al Ta'ani, Yazan Al Thaher, Rasmieh Mustafa Al-amer, Mohammed Albashtawy, Abdelazeem M Algammal, Khalid F Alhabib, Dari Alhuwail, Abid Ali, Waad Ali, Sheikh Mohammad Alif, Syed Mohamed Aljunid, Intima Alrimawi, Awais Altaf, Nelson Alvis-Guzman, Mohammad Al-Wardat, Hosam Alzahrani, Abdallah Alzoubi, Md. Akib Al-Zubayer, Hubert Amu, Ranjit Mohan Anjana, Sumbul Ansari, Saeid Anvari, Saleha Anwar, Geminn Louis Carace Apostol, Juan Pablo Arab, Jalal Arabloo, Hidayat Arifin, Benedetta Armocida, Tahira Ashraf, Seyyed Shamsadin Athari, Prince Atorkey, Alok Atreya, Zaure Maratovna Aumoldaeva, Adedapo Wasiu Awotidebe, Setognal Birara Aychiluhm, Ahmed Y. Azzam, Domenico Azzolino, Mina Babashahi, Giridhara

Rathnaiah Babu, Ashish D Badiye, Senthilkumar Balakrishnan, Ovidiu Constantin Baltatu, Maciej Banach, Rajon Banik, Mainak Bardhan, Hiba Jawdat Barqawi, Zarrin Basharat, Shahid Bashir, Mohammad-Mahdi Bastan, Mulat Tirfie Bayih, Narasimha M Beeraka, Abdulrahman Babatunde Bello, Akshaya Srikanth Bhagavathula, Ravi Bharadwaj, Sonu Bhaskar, Ajay Nagesh Bhat, Priyadarshini Bhattacharjee, Gurjit Kaur Bhatti, Jasvinder Singh Bhatti, Cem Bilgin, Bijit Biswas, Espen Bjertness, Hamed Borhany, Souad Bouaoud, Dejana Braithwaite, Dana Bryazka, Felix Busch, Luciana Aparecida Campos, Monica Cattafesta, Francieli Cembranel, Joshua Chadwick, Vijay Kumar Chattu, Akhilanand Chaurasia, Guangjin Chen, Nicholas WS Chew, Ritesh Chimoriya, Bryan Chong, Hitesh Chopra, Shivani Chopra, Dinh-Toi Chu, Sunghyun Chung, Alyssa Columbus, Nathalie Conrad, Michael H Criqui, Natalia Cruz-Martins, Xiaochen Dai, Lalit Dandona, Rakhi Dandona, Lucio D'Anna, Samuel Demissie Darcho, Reza Darvishi Cheshmeh Soltani, Meseret Derbew Molla, Ismail Dergaa, Vinoth Gnana Chellaiyan Devanbu, Devananda Devegowda, Samath Dhamminda Dharmaratne, Meghnath Dhimal, Klara Georgieva Dokova, Fariba Dorostkar, Ojas Prakashbhai Doshi, Rajkumar Prakashbhai Doshi, Robert Kokou Dowou, Bruce B Duncan, Alireza Ebrahimi, Michael Ekholuenetale, Rabie Adel El Arab, Ibrahim Farahat El Bayoumy, Ashraf A El-Metwally, Mohamed A Elmonem, Chadi Eltaha, Ugochukwu Anthony Eze, Heidar Fadavian, Adeniyi Francis Fagbamigbe, Ildar Ravisovich Fakhradiyev, Carla Sofia e Sá Farinha, Hossein Farrokhpour, Timur Fazylov, Alireza Feizkhah, Morenike Oluwatoyin Folayan, Maryam Fotouhi, Muktar A Gadanya, Emmanuela Gakidou, Yaseen Galali, Silvano Gallus, Balasankar Ganesan, Teferi Gebru Gebremeskel, Lemma Getacher, Mahaveer Golechha, Davide Golinelli, Shi-Yang Guan, Avirup Guha, Rajeev Gupta, Sapna Gupta, Najah R Hadi, Zahra Hadian, Nadia M Hamdy, Mohammad Hamiduzzaman, Nasrin Hanifi, Allie Haq, Josep Maria Haro, Faizul Hasan, Soheil Hassanipour, Rasmus J Havmoeller, Wen-Qiang He, Jeffrey J Hebert, Golnaz Heidari, Mehdi Hemmati, Nguyen Quoc Hoan, Mai Hoang, Praveen Hoogar, Hong-Han Huynh, Olayinka Stephen Ilesanmi, Lalu Muhammad Irham, Sheikh Mohammed Shariful Islam, Nahlah Elkudssiah Ismail, Gaetano Isola, Haitham Jahrami, Ammar Abdulrahman Jairoun, Mihajlo Jakovljevic, Mohamed Lamrana Jalloh, Syed Sarmad Javaid, Sathish Kumar Jayapal, Shubha Jayaram, Jost B Jonas, Charity Ehimwenma Joshua, Jacek Jerzy Jozwiak, Mikk Jürisson, Billingsley Kaambwa, Zubair Kabir, Sivesh Kathir Kamarajah, Samuel Berchi Kankam, Neeti Kapoor, Nicholas J Kassebaum, Gbenga A Kayode, Dimitrios Kehagias, Ariz Keshwani, Himanshu Khajuria, Maseer Khan, Mohammad Jobair Khan, Moien AB Khan, Khaled Khatab, Feriha Fatima Khidri, Atulya Aman Khosla, Min Seo Kim, Yun Jin Kim, Adnan Kisa, Michail Kokkorakis, Oleksii Korzh, Karel Kostev, Sindhura Lakshmi Koulmane Laxminarayana, Irene Akwo Kretchy, Kewal Krishan, Chong-Han Kua, Barthelemy Kuate Defo, G Anil Kumar, Vijay Kumar, Satyajit Kundu, Dian Kusuma, Ville Kytö, Chandrakant Lahariya, Kamaluddin Latief, Nhi Huu Hanh Le, Munjae Lee, Sang-woong Lee, Seung Won Lee, Wei-Chen Lee, Yongze Li, Stephen S Lim, Queran Lin, Simin Liu, Erand Llanaj, Justin Lo, Stefan Lorkowski, Alessandra Lugo, Ellina Lytvyak, Zheng Feei Ma, Nastaran Maghbouli, Nozad H Mahmood, Elham Mahmoudi, Kashish Malhotra, Deborah Carvalho Malta, Sammer Marzouk, Manu Raj Mathur, Steven M McPhail, Enkeleint A Mechili, Tesfahun Mekene Meto, Walter Mendoza, Ritesh G Menezes, Ana Carolina Micheletti Gomide Nogueira de Sá, Erkin M Mirrakhimov, Awoke Misganaw, Mona Gamal Mohamed, Nouh Saad Mohamed, Mustapha Mohammed, Shafiu Mohammed, Ali H Mokdad, Rohith Motappa, Francesk Mulita, Erin C Mullany, Yanjinlkham Munkhsaikhan, Efren Murillo-Zamora, Christopher J L Murray, Mohsen Naghavi, Ganesh R Naik, Gopal Nambi, Zuhair S Natto, Biswa Prakash Nayak, Masoud Negahdary, Ionut Negoi, Ruxandra Irina Negoi, Henok Biresaw Netsere, Josephine W Ngunjiri, Cuong Tat Nguyen, Dang Nguyen, Trang Nguyen, Robina Khan Niazi, Luciano Nieddu, Shuhei Nomura, Syed Toukir Ahmed Noor, Mamoona Noreen, Masoud Noroozi, Jean Jacques Noubiap, Mehran Nouri, Fred Nugen, Dieta Nurrika, Bogdan Oancea, Erin M

O'Connell, Akinkunmi Paul Okekunle, Andrew T Olagunju, Oladotun Victor Olalusi, Yinka Doris Oluwafemi, Hany A Omar, Ahmed Omar Bali, Uchechukwu Levi Osuagwu, Abdu Oumer, Amel Ouyahia, Mayowa O Owolabi, Mahesh Padukudru P A, Jagadish Rao Padubidri, Sujogya Kumar Panda, Songhomitra Panda-Jonas, Anamika Pandey, Seithikurippu R Pandi-Perumal, Shahina Pardhan, Romil R Parikh, Eun-Cheol Park, Hemal M Patel, Aslam Ramjan Pathan, Prince Peprah, Gavin Pereira, Arokiasamy Perianayagam, Hoang Nhat Pham, Jalandhar Pradhan, Elton Junio Sady Prates, Jagadeesh Puvvula, Ibrahim Qattea, Jia-Yong Qiu, Venkatraman Radhakrishnan, Catalina Raggi, Jeffrey Pradeep Raj, Mahmoud Mohammed Ramadan, Chitra Ramasamy, Shakthi Kumaran Ramasamy, Sheena Ramazanu, Kritika Rana, Chhabi Lal Ranabhat, Mithun Rao, Sowmya J Rao, Sina Rashedi, Santosh Kumar Rauniyar, Ilari Rautalin, Salman Rawaf, Hossein Rezazadeh, Mónica Rodrigues, Jefferson Antonio Buendia Rodriguez, Leonardo Roever, Peter Rohloff, Debby Syahru Romadlon, Moustaq Karim Khan Rony, Godfrey M Rwegerera, Aly M A Saad, Cameron John Sabet, Kabir P Sadarangani, Basema Ahmad Saddik, Umar Saeed, Sher Zaman Safi, Mirza Rizwan Sajid, Marwa Rashad Salem, Abdallah M Samy, Prasanna K Santhekadur, Milena M Santric-Milicevic, Ushasi Saraswati, Brijesh Sathian, Maria Inês Schmidt, Sneha Annie Sebastian, Siddharthan Selvaraj, Mohammad H Semreen, Yigit Can Senol, Subramanian Senthilkumaran, Yashendra Sethi, Allen Seylani, Syed Mahboob Shah, Samiah Shahid, Sunder Sham, Muhammad Aaqib Shamim, Mehran Shams-Beyranvand, Anas Shamsi, Dan Shan, Mohammed Shannawaz, Medha Sharath, Amin Sharifan, Vishal Sharma, Kenji Shibuya, Aminu Shittu, Sina Shool, Mithun Sikdar, Luís Manuel Lopes Rodrigues Silva, Baljinder Singh, Harmanjit Singh, Jasvinder A Singh, Kalpana Singh, Valentin Yurievich Skryabin, Anna Aleksandrovna Skryabina, Georgia Smith, Reed J D Sorensen, Michael Spartalis, Bahadar S Srichawla, Vetriselvan Subramaniyan, Aleksander Sulkowski, Chandan Kumar Swain, Lukasz Szarpak, Sree Sudha T Y, Rafael Tabarés-Seisdedos, Shima Tabatabai, Celine Tabche, Jabeen Taiba, Stella Talic, Mircea Tampa, Ker-Kan Tan, Manoj Tanwar, Dufera Rikitu Terefa, Jay Tewari, Krishna Tiwari, Marcello Tonelli, Mathilde Touvier, Marcos Roberto Tovani-Palone, Mai Thi Ngoc Tran, Domenico Trico, Indang Trihandini, Guesh Mebrahtom Tsegay, Munkhtuya Tumurkhuu, Sok Cin Tye, Shahid Ullah, Muhammad Umair, Era Upadhyay, Jibrin Sammani Usman, Jef Van den Eynde, Joe Varghese, Tommi Juhani Vasankari, Balachandar Vellingiri, Narayanaswamy Venketasubramanian, Anjul Verma, Georgios-Ioannis Verras, Vasily Vlassov, Stein Emil Vollset, Theo Vos, Shu Wang, Xingxin Wang, Anggi Lukman Wicaksana, Dakshitha Praneeth Wickramasinghe, Peter Willeit, Tewodros Eshete Wonde, Utoomporn Wongsin, Suowen Xu, Kazumasa Yamagishi, Subah Abderehim Yesuf, Naohiro Yonemoto, Chuanhua Yu, Deniz Yuce, Ismaeel Yunusa, Iman Zare, Michael Zastrozhin, Jingya Zhang, Xiaoyi Zhang, Anthony Zhong, Abzal Zhumagaliuly, and Magdalena Zielińska.

Developing methods or computational machinery

Austin J Ahlstrom, Aleksandr Y Aravkin, Dana Bryazka, Rebecca M Cogen, Xiaochen Dai, Emmanuela Gakidou, Nicholas J Kassebaum, Justin Lo, Ali H Mokdad, Christopher J L Murray, Mohsen Naghavi, Erin M O'Connell, George C Patton, Catalina Raggi, Reed J D Sorensen, Stein Emil Vollset, Theo Vos, Chun-Wei Yuan, and Peng Zheng.

Providing critical feedback on methods or results

Yohannes Habtegiorgis Abate, Cristiana Abbafati, Nasir Abbas, Mohammadreza Abbasian, Samar Abd ElHafeez, Wael M Abdel-Rahman, Sherief Abd-Elsalam, Arash Abdollahi, Meriem Abdoun, Deldar Morad Abdulah, Rizwan Suliankatchi Abdulkader, Auwal Abdullahi, Armita Abedi, Hansani Madushika Abeywickrama, Alemwork Abie, Richard Gyan Aboagye, Shady Abohashem, Dariush Abtahi, Rana Kamal Abu Farha, Bilyaminu Abubakar, Hana J Abukhadijah, Salahdein Aburuz, Ahmed Abu-Zaid, Lisa C Adams,

Mesafint Molla Adane, Isaac Yeboah Addo, Kamoru Ademola Adedokun, Nurudeen A Adegoke, Abiola Victor Victor Adepoju, Ridwan Olamilekan Adesola, Temitayo Esther Adeyeoluwa, Usha Adiga, Qorinah Estiningtyas Sakilah Adnani, Siamak Afaghi, Muhammad Sohail Afzal, Saira Afzal, Thilini Chanchala Agampodi, Shahin Aghamiri, César Agostinis Sobrinho, Williams Agyemang-Duah, Aqeel Ahmad, Danish Ahmad, Fuzail Ahmad, Muayyad M Ahmad, Sajjad Ahmad, Ayman Ahmed, Haroon Ahmed, Mehrunnisha Sharif Ahmed, Muktar Beshir Ahmed, Syed Anees Ahmed, Ashley E Akrami, Hanadi Al Hamad, Syed Mahfuz Al Hasan, Omar Al Ta'ani, Zain Al Ta'ani, Yazan Al Thaher, Ziyad Al-Aly, Khurshid Alam, Rasmieh Mustafa Al-amer, Amani Alansari, Fahmi Y Al-Ashwal, Mohammed Albashtawy, Bezawit Abeje Alemayehu, Abdelazeem M Algammal, Khalid F Alhabib, Dari Alhuwail, Abid Ali, Mohammed Usman Ali, Rafat Ali, Waad Ali, Sheikh Mohammad Alif, Samah W Al-Jabi, Syed Mohamed Aljunid, Ahmad Alkhatib, Sabah Al-Marwani, Mahmoud A Alomari, Saleh A Alqahtani, Rajaa M Mohammad Al-Raddadi, Intima Alrimawi, Najim Z Alshahrani, Zaid Altaany, Awais Altaf, Nelson Alvis-Guzman, Mohammad Al-Wardat, Yaser Mohammed Al-Worafi, Hany Aly, Safwat Aly, Hosam Alzahrani, Abdallah Alzoubi, Karem H Alzoubi, Md. Akib Al-Zubayer, Sohrab Amiri, Hubert Amu, Dickson A Amugsi, Ganiyu Adeniyi Amusa, Roshan A Ananda, Robert Ancuceanu, Catalina Liliana Andrei, Ranjit Mohan Anjana, Mohammed Tahir Ansari, Sumbul Ansari, Boluwatife Stephen Anuoluwa, Iyadunni Adesola Anuoluwa, Saeid Anvari, Saleha Anwar, Anayochukwu Edward Anyasodor, Geminn Louis Carace Apostol, Juan Pablo Arab, Jalal Arabloo, Mosab Arafat, Demelash Areda, Hidayat Arifin, Mesay Arkew, Mahwish Arooj, Anton A Artamonov, Kurnia Dwi Artanti, Ashokan Arumugam, Mohammad Asghari-Jafarabadi, Tahira Ashraf, Bernard Kwadwo Yeboah Asiamah-Asare, Anemaw A Asrat, Thomas Astell-Burt, Seyyed Shamsadin Athari, Prince Atorkey, Alok Atreya, Hamzeh Awad, Mamaru Ayenew Awoke, Setognal Birara Aychiluhm, Ali Azargoonjahromi, Amirali Azimi, Sadat Abdulla Aziz, Shahkaar Aziz, Ahmed Y. Azzam, Domenico Azzolino, Peter S Azzopardi, Mina Babashahi, Giridhara Rathnaiah Babu, Nasser Bagheri, Yogesh Bahurupi, Ruhai Bai, Senthilkumar Balakrishnan, Ovidiu Constantin Baltatu, Maciej Banach, Rajon Banik, Mainak Bardhan, Hiba Jawdat Barqawi, Simon Barquera, Zarrin Basharat, Shahid Bashir, Mohammad-Mahdi Bastan, Saurav Basu, Reza Bayat, Mulat Tirfie Bayih, Narasimha M Beeraka, Tahmina Begum, Abdulrahman Babatunde Bello, Umar Muhammad Bello, Abiye Assefa Berihun, Ajeet Singh Bhadoria, Akshaya Srikanth Bhagavathula, Neeraj Bhala, Jaideep Singh Bhalla, Ravi Bharadwaj, Nikha Bhardwaj, Pankaj Bhardwaj, Sonu Bhaskar, Ajay Nagesh Bhat, Priyadarshini Bhattacharjee, Shuvarthi Bhattacharjee, Gurjit Kaur Bhatti, Jasvinder Singh Bhatti, Andras Bikov, Cem Bilgin, Bijit Biswas, Bruno Bizzozero Peroni, Espen Bjertness, Hamed Borhany, Samuel Adolf Bosoka, Souad Bouaoud, Edward J Boyko, Dejana Braithwaite, Javier Brazo-Sayavera, Hermann Brenner, Gabrielle Britton, Dana Bryazka, Raffaele Bugiardini, Linh Phuong Bui, Felix Busch, Yasser Bustanji, Nadeem Shafique Butt, Zahid A Butt, Luciana Aparecida Campos, Ismael Campos-Nonato, Si Cao, Andre F Carvalho, Márcia Carvalho, Monica Cattafesta, Luca Cegolon, Francieli Cembranel, Edina Cenko, Ester Cerin, Achille Cernigliaro, Joshua Chadwick, Chiranjib Chakraborty, Raymond N C Chan, Jung-Chen Chang, Vijay Kumar Chattu, Akhilanand Chaurasia, An-Tian Chen, Guangjin Chen, Haowei Chen, Esther T W Cheng, Nicholas WS Chew, Gerald Chi, Ritesh Chimoriya, Dong-Woo Choi, Bryan Chong, Hitesh Chopra, Shivani Chopra, Hou In Chou, Sonali Gajanan Choudhari, Dinh-Toi Chu, Sheng-Chia Chung, Sunghyun Chung, Muhammad Chutiyami, Karly I Cini, Iolanda Cioffi, Alyssa Columbus, Nathalie Conrad, Michael H Criqui, Natalia Cruz-Martins, Alanna Gomes da Silva, Omid Dadras, Xiaochen Dai, Mayank Dalakoti, Lucio D'Anna, Pojsakorn Danpanichkul, Samuel Demissie Darcho, Reza Darvishi Cheshmeh Soltani, Kairat Davletov, Ivan Delgado-Enciso, Meseret Derbew Molla, Ismail Dergaa, Aragaw Tesfaw Desale, Vinoth Gnana Chellaiyan Devanbu, Devananda Devegowda, Arkadeep Dhali, Samath Dhamminda Dharmaratne, Meghnath Dhimal, Bibha Dhungel, Daniel Diaz,

Camila Bruneli do Prado, Milad Dodangeh, Neda Dolatkhah, Mario D'Oria, Ojas Prakashbhai Doshi, Rajkumar Prakashbhai Doshi, Robert Kokou Dowou, Viola Savy Dsouza, Mi Du, Dorothea Dumuid, Sulagna Dutta, Arkadiusz Marian Dziedzic, Alireza Ebrahimi, Ashkan Eighaei Sedeh, Michael Ekholuenetale, Rabie Adel El Arab, Ibrahim Farahat El Bayoumy, Mohamed Ahmed Eladl, Islam Y Elgendy, Muhammed Elhadi, Waseem El-Huneidi, Ashraf A El-Metwally, Mohamed Hassan Elnaem, Randa Elsheikh, Ibrahim Elsohaby, Chadi Eltaha, Theophilus I Emeto, Ugochukwu Anthony Eze, Heidar Fadavian, Adeniyi Francis Fagbamigbe, Ildar Ravisovich Fakhradiyev, Seyed Nooreddin Faraji, Carla Sofia e Sá Farinha, MoezAlIslam Ezzat Mahmoud Faris, Umar Farooque, Hossein Farrokhpour, Samuel Aanuoluwapo Fasusi, Patrick Fazeli, Alireza Feizkhah, Ginenus Fekadu, Xiaoqi Feng, João C Fernandes, Bikila Regassa Feyisa, Florian Fischer, David Flood, Nataliya A Foigt, Artem Alekseevich Fomenkov, Roham Foroumadi, Celia Fortuna Rodrigues, Matteo Foschi, Maryam Fotouhi, Kate Louise Francis, Richard Charles Franklin, Aleš Gába, Muktar A Gadanya, Abhay Motiramji Gaidhane, Emmanuela Gakidou, Yaseen Galali, Silvano Gallus, Balasankar Ganesan, Wendy Paola Gastélum Espinoza, Miglas Welay Gebregergis, Teferi Gebru Gebremeskel, Lemma Getacher, Amir Ghaffari Jolfayi, Ramy Mohamed Ghazy, Tiffany K Gill, Elena V Gnedovskaya, Mahaveer Golechha, Michal Grivna, Ashna Grover, Shi-Yang Guan, Zhongyang Guan, Mohammed Ibrahim Mohialdeen Gubari, Avirup Guha, Damitha Asanga Gunawardane, Zheng Guo, Anish Kumar Gupta, Rahul Gupta, Sapna Gupta, Vivek Kumar Gupta, Roberth Steven Gutiérrez-Murillo, Jose Guzman-Esquivel, Najah R Hadi, Zahra Hadian, Nadia M Hamdy, Samer Hamidi, Mohammad Hamiduzzaman, Asif Hanif, Nasrin Hanifi, Allie Haq, Netanja I Harlianto, Risky Kusuma Hartono, Faizul Hasan, Md Saquib Hasnain, Soheil Hassanipour, Afagh Hassanzade Rad, Rasmus J Havmoeller, Simon I Hay, Wen-Qiang He, Golnaz Heidari, Mehdi Hemmati, Yuta Hiraike, Nguyen Quoc Hoan, Mai Hoang, Ramesh Holla, Praveen Hoogar, Ashley Mark Hopkins, Alamgir Hossain, Hassan Hosseinzadeh, Mihaela Hostiuc, Chengxi Hu, Junjie Huang, Tsegaye Gebreyes Hundie, Mohamed Ibrahim Husseiny, Hong-Han Huynh, Olayinka Stephen Ilesanmi, Irena M Ilic, Milena D Ilic, Mohammad Tarique Imam, Leeberk Raja Inbaraj, Arit Inok, Lalu Muhammad Irham, Md. Rabiul Islam, Sheikh Mohammed Shariful Islam, Nahlah Elkudssiah Ismail, Gaetano Isola, Mosimah Charles Ituka, Masao Iwagami, Chinwe Juliana Iwu-Jaja, Assefa N Iyasu, Vinothini J, Louis Jacob, Shabbar Jaffar, Haitham Jahrami, Akhil Jain, Ammar Abdulrahman Jairoun, Mihajlo Jakovljevic, Mohamed Lamrana Jalloh, Syed Sarmad Javaid, Sathish Kumar Jayapal, Umesh Jayarajah, Shubha Jayaram, Rime Jebai, Felix K Jebasingh, Alelign Tasew Jema, Mohammad Jokar, Jost B Jonas, Jobinse Jose, Nitin Joseph, Charity Ehimwenma Joshua, Jacek Jerzy Jozwiak, Mikk Jürisson, Billingsley Kaambwa, Ali Kabir, Zubair Kabir, Ashish Kumar Kakkar, Sanjay Kalra, Sivesh Kathir Kamarajah, Saddam Fuad Kanaan, Samuel Berchi Kankam, Kehinde Kazeem Kanmodi, Mehrdad Karajizadeh, Paschalis Karakasis, Yeganeh Karimi, Arman Karimi Behnagh, Nicholas J Kassebaum, Gbenga A Kayode, Dimitrios Kehagias, Jessica A Kerr, Ariz Keshwani, Mohammad Keykhaei, Inn Kynn Khaing, Himanshu Khajuria, Pantea Khalili, Alireza Khalilian, Mohamed Khalis, Ajmal Khan, Maseer Khan, Md Abdullah Saeed Khan, Mohammad Jobair Khan, Moien AB Khan, Nusrat Khan, Shaghayegh Khanmohammadi, Khaled Khatab, Moawiah Mohammad Khatatbeh, Maryam Khayamzadeh, Feriha Fatima Khidri, Fatemeh Khorashadizadeh, Atulya Aman Khosla, Jagdish Khubchandani, Helda Khusun, Jinho Kim, Kwanghyun Kim, Min Seo Kim, Yun Jin Kim, Ruth W Kimokoti, Adnan Kisa, Ladli Kishore, Mika Kivimäki, Michail Kokkorakis, Ali-Asghar Kolahi, Farzad Kompani, Oleksii Korzh, Sindhura Lakshmi Koulmane Laxminarayana, Irene Akwo Kretchy, Kewal Krishan, Chong-Han Kua, Barthelemy Kuate Defo, Ashish Kumar, Vijay Kumar, Satyajit Kundu, Setor K Kunutsor, Om P Kurmi, Maria Dyah Kurniasari, Dian Kusuma, Ville Kytö, Carlo La Vecchia, Chandrakant Lahariya, Daphne Teck Ching Lai, Hanpeng Lai, Iván Landires, Bagher Larijani, Kamaluddin Latief, Nhi Huu Hanh Le, Munjae Lee,

Sang-woong Lee, Seung Won Lee, Wei-Chen Lee, Ming-Chieh Li, Weilong Li, Yongze Li, Stephen S Lim, Jialing Lin, Queran Lin, Daniel Lindholm, Simin Liu, Erand Llanaj, Justin Lo, José Francisco López-Gil, Stefan Lorkowski, Giancarlo Lucchetti, Lei Lv, Ellina Lytvyak, Zheng Feei Ma, Monika Machoy, Javier A Magaña Gómez, Nastaran Maghbouli, Mehrdad Mahalleh, Nozad H Mahmood, Elham Mahmoudi, Rituparna Maiti, Konstantinos Christos C Makris, Kashish Malhotra, Ahmad Azam Malik, Deborah Carvalho Malta, Marjan Mansourian, Emmanuel Manu, Hamid Reza Marateb, Mirko Marino, Ramon Martinez-Piedra, Santi Martini, Miquel Martorell, Sammer Marzouk, Stefano Masi, Soroush Masrouri, Yasith Mathangasinghe, Manu Raj Mathur, Fernanda Penido Matozinhos, Thushara Matthias, Rita Mattiello, Mohsen Mazidi, Steven M McPhail, Enkeleint A Mechili, Riffat Mehboob, Asim Mehmood, Man Mohan Mehndiratta, Kamran Mehrabani-Zeinabad, Tesfahun Mekene Meto, Hadush Negash Meles, Walter Mendoza, Ritesh G Menezes, Emiru Ayalew Mengistie, Sultan Ayoub Meo, Tomislav Mestrovic, Chamila Dinushi Kukulege Mettananda, Sachith Mettananda, Ana Carolina Micheletti Gomide Nogueira de Sá, Ted R Miller, GK Mini, Erkin M Mirrakhimov, Madhukar Mittal, Ahmed Ismail Mohamed, Jama Mohamed, Mona Gamal Mohamed, Nouh Saad Mohamed, Taj Mohammad, Sakineh Mohammad-Alizadeh-Charandabi, Ibrahim Mohammadzadeh, Mustapha Mohammed, Shafiu Mohammed, Ali H Mokdad, Stefania Mondello, Mohammad Ali Moni, Maryam Moradi, Shane Douglas Morrison, Elias Mossialos, Rohith Motappa, Francesk Mulita, Erin C Mullany, Yanjinlkham Munkhsaikhan, Efren Murillo-Zamora, Christopher J L Murray, Ghulam Mustafa, Sathish Muthu, Mohsen Naghavi, Pirouz Naghavi, Ganesh R Naik, Hiten Naik, Gopal Nambi, Vinay Nangia, Jobert Richie Nansseu, Mahmoud Nassar, Zuhair S Natto, Javaid Nauman, Zakira Naureen, Biswa Prakash Nayak, Md Fahad Shahariar Nayon, Athare Nazri-Panjaki, Masoud Negahdary, Ionut Negoi, Ruxandra Irina Negoi, Seyed Aria Nejadghaderi, Soroush Nematollahi, Henok Biresaw Netsere, Josephine W Ngunjiri, Cuong Tat Nguyen, Dang Nguyen, Duc Hoang Nguyen, Phuong The Nguyen, Trang Nguyen, Robina Khan Niazi, Luciano Nieddu, Ali Nikoobar, Shuhei Nomura, Syed Toukir Ahmed Noor, Mamoona Noreen, Masoud Noroozi, Nawsherwan, Jean Jacques Noubiap, Mehran Nouri, Chisom Adaobi Nri-Ezedi, Fred Nugen, Dieta Nurrika, Ogochukwu Janet Nzoputam, Bogdan Oancea, Erin M O'Connell, James Odhiambo Oguta, Hassan Okati-Aliabad, Akinkunmi Paul Okekunle, Osaretin Christabel Okonji, Andrew T Olagunju, Oladotun Victor Olalusi, Omotola O Olasupo, Yinka Doris Oluwafemi, Hany A Omar, Ahmed Omar Bali, Marcel Opitz, Michal Ordak, Wael M S Osman, Uchechukwu Levi Osuagwu, Adrian Otoiu, Abdu Oumer, Amel Ouyahia, Mayowa O Owolabi, Irene Amoakoh Owusu, Kolapo Oyebola, Mahesh Padukudru P A, Jagadish Rao Padubidri, Sujogya Kumar Panda, Songhomitra Panda-Jonas, Seithikurippu R Pandi-Perumal, Shahina Pardhan, Utsav Parekh, Pragyan Paramita Parija, Romil R Parikh, Eun-Cheol Park, Ava Pashaei, Roberto Passera, Hemal M Patel, Aslam Ramjan Pathan, Dimitrios Patoulias, George C Patton, Susan Paudel, Prince Peprah, Gavin Pereira, Marcos Pereira, Simone Perna, Ionela-Roxana Petcu, Fanny Emily Petermann-Rocha, Hoang Nhat Pham, Roman V Polibin, Farzad Pourghazi, Akram Pourshams, Jalandhar Pradhan, Pranil Man Singh Pradhan, Manya Prasad, Akila Prashant, Elton Junio Sady Prates, I Gusti Ngurah Edi Putra, Jagadeesh Puvvula, Ibrahim Qattea, Venkatraman Radhakrishnan, Maja R Radojčić, Fryad Majeed Rahman, Mohammad Hifz Ur Rahman, Mosiur Rahman, Muhammad Aziz Rahman, Saeed Rahmani, Vahid Rahmanian, Setyaningrum Rahmawaty, Rajesh Kumar Rai, Jeffrey Pradeep Raj, Prashant Rajput, Mahmoud Mohammed Ramadan, Chitra Ramasamy, Shakthi Kumaran Ramasamy, Kritika Rana, Mithun Rao, Sowmya J Rao, Sina Rashedi, Mohammad-Mahdi Rashidi, Ashkan Rasouli-Saravani, Devarajan Rathish, Santosh Kumar Rauniyar, Ilari Rautalin, David Laith Rawaf, Salman Rawaf, Elrashdy M. Moustafa Mohamed Redwan, Sanika Rege, Nazila Rezaei, Mohsen Rezaeian, Hossein Rezazadeh, Taeho Gregory Rhee, João Rocha Rocha-Gomes, Mónica Rodrigues, Thales Philipe Rodrigues da Silva, Jefferson Antonio

Buendia Rodriguez, Leonardo Roever, Peter Rohloff, Debby Syahru Romadlon, Moustaq Karim Khan Rony, Gholamreza Roshandel, Himanshu Sekhar Rout, Nitai Roy, Godfrey M Rwegerera, Aly M A Saad, Maha Mohamed Saber-Ayad, Cameron John Sabet, Kabir P Sadarangani, Basema Ahmad Saddik, Mohammad Reza Saeb, Umar Saeed, Sahar Saeedi Moghaddam, Sher Zaman Safi, Amene Saghazadeh, Fatemeh Saheb Sharif-Askari, Soumya Swaroop Sahoo, Mirza Rizwan Sajid, Mohamed A Saleh, Marwa Rashad Salem, Sohrab Salimi, Yoseph Leonardo Samodra, Vijaya Paul Samuel, Abdallah M Samy, Prasanna K Santhekadur, Milena M Santric-Milicevic, Muhammad Arif Nadeem Saqib, Ushasi Saraswati, Dianis Wulan Sari, Tanmay Sarkar, Mohammad Sarmadi, Gargi Sachin Sarode, Sachin C Sarode, Michele Sassano, Brijesh Sathian, Susan M Sawyer, Ganesh Kumar Saya, Christophe Schinckus, Art Schuermans, Aletta Elisabeth Schutte, Sneha Annie Sebastian, Siddharthan Selvaraj, Mohammad H Semreen, Ashenafi Kibret Sendekie, Pallav Sengupta, Yigit Can Senol, Subramanian Senthilkumaran, Sadaf G Sepanlou, Yashendra Sethi, Mahan Shafie, Sweni Shah, Syed Mahboob Shah, Samiah Shahid, Fatemeh Shahrahmani, Muhammad Aaqib Shamim, Mehran Shams-Beyranvand, Anas Shamsi, Alfiya Shamsutdinova, Dan Shan, Mohd Shanawaz, Mohammed Shannawaz, Medha Sharath, Sadaf Sharfaei, Amin Sharifan, Anupam Sharma, Ujjawal Sharma, Vishal Sharma, Fateme Sheida, Ali Sheikhy, Rekha Raghuveer Shenoy, Kenji Shibuya, Desalegn Shiferaw, Rahman Shiri, Aminu Shittu, Sina Shool, Seyed Afshin Shorofi, Rajan Shrestha, Kerem Shuval, Yafei Si, Nicole R S Sibuyi, Emmanuel Edwar Siddig, Ahmed Kamal Siddiqi, Mithun Sikdar, Diego Augusto Santos Silva, Luís Manuel Lopes Rodrigues Silva, Amit Singh, Baljinder Singh, Harmanjit Singh, Jasvinder A Singh, Kalpana Singh, Puneetpal Singh, Valentin Yurievich Skryabin, Anna Aleksandrovna Skryabina, Amanda E Smith, Sameh S M Soliman, Soroush Soraneh, Reed J D Sorensen, Michael Spartalis, Bahadar S Srichawla, Antonina V Starodubova, Kurt Straif, Pete Stubbs, Vetriselvan Subramaniyan, Muritala Odidi Suleiman Odidi, Aleksander Sulkowski, Anusha Sultan Meo, Jing Sun, Zhong Sun, Sumam Sunny, Chandan Kumar Swain, Lukasz Szarpak, Sree Sudha T Y, Rafael Tabarés-Seisdedos, Fatemeh Sadat Tabatabaei, Seyyed Mohammad Tabatabaei, Ozra Tabatabaei Malazy, Shima Tabatabai, Celine Tabche, Mohammad Tabish, Jabeen Taiba, Stella Talic, Mircea Tampa, Jacques Lukenze Tamuzi, Ker-Kan Tan, Manoj Tanwar, Seyed Mohammad Tavangar, Mohamad-Hani Temsah, Masayuki Teramoto, Dufera Rikitu Terefa, Jay Tewari, Rekha Thapar, Jansje Henny Vera Ticoalu, Sofonyas Abebaw Tiruneh, Tenaw Yimer Tiruye, Mariya Vladimirovna Titova, Krishna Tiwari, Sojit Tomo, Marcello Tonelli, Mathilde Touvier, Marcos Roberto Tovani-Palone, Khaled Trabelsi, Mai Thi Ngoc Tran, Nguyen Tran Minh Duc, Domenico Trico, Indang Trihandini, Guesh Mebrahtom Tsegay, Munkhtuya Tumurkhuu, Sok Cin Tye, Stefanos Tyrovolas, Aniefiok John Udoakang, Saeed Ullah, Shahid Ullah, Muhammad Umair, Lawan Umar, Umar Muhammad Umar, Dinesh Upadhya, Era Upadhyay, Jibrin Sammani Usman, Jef Van den Eynde, Joe Varghese, Siavash Vaziri, Balachandar Vellingiri, Narayanaswamy Venketasubramanian, Madhur Verma, Georgios-Ioannis Verras, Victor E Villalobos-Daniel, Manish Vinayak, Vasily Vlassov, Stein Emil Vollset, Theo Vos, Rade Vukovic, Mugi Wahidin, Mohammad Wahiduzzaman, Cong Wang, Shu Wang, Xingxin Wang, Yanzhong Wang, Ahmed Bilal Waqar, Muhammad Waqas, Kosala Gayan Weerakoon, Fei-Long Wei, Anggi Lukman Wicaksana, Dakshitha Praneeth Wickramasinghe, Peter Willeit, Tewodros Eshete Wonde, Utoomporn Wongsin, Qing Xia, Wanqing Xie, Suowen Xu, Xiaoyue Xu, Yuichiro Yano, Haiqiang Yao, Amir Yarahmadi, Habib Yaribeygi, Subah Abderehim Yesuf, Dehui Yin, Dong Keon Yon, Naohiro Yonemoto, Deniz Yuce, Ismaeel Yunusa, Sojib Bin Zaman, Michael Zastrozhin, Mohammed G M Zeariya, Casper J P Zhang, Jingya Zhang, Liqun Zhang, Xiaoyi Zhang, Zhiqiang Zhang, David X Zheng, Anthony Zhong, Claire Chenwen Zhong, Jiayan Zhou, Bin Zhu, Magdalena Zielińska, Zhiyong Zou, Elric Zweck, and Sa'ed H Zyoud.

Drafting the work or revising it critically for important intellectual content

Yohannes Habtegiorgis Abate, Cristiana Abbafati, Samar Abd ElHafeez, Wael M Abdel-Rahman, Sherief Abd-Elsalam, Arash Abdollahi, Auwal Abdullahi, Armita Abedi, Hansani Madushika Abeywickrama, Shady Abohashem, Rana Kamal Abu Farha, Hasan Abualruz, Hana J Abukhadijah, Niveen ME Abu-Rmeileh, Salahdein Aburuz, Ahmed Abu-Zaid, Lisa C Adams, Mesafint Molla Adane, Isaac Yeboah Addo, Kamoru Ademola Adedokun, Nurudeen A Adegoke, Abiola Victor Victor Adepoju, Usha Adiga, Qorinah Estiningtyas Sakilah Adnani, Siamak Afaghi, Muhammad Sohail Afzal, Saira Afzal, Thilini Chanchala Agampodi, César Agostinis Sobrinho, Danish Ahmad, Fuzail Ahmad, Muayyad M Ahmad, Ayman Ahmed, Haroon Ahmed, Mehrunnisha Sharif Ahmed, Muktar Beshir Ahmed, Syed Anees Ahmed, Marjan Ajami, Samina Akhtar, Mohammed Ahmed Akkaif, Omar Al Ta'ani, Zain Al Ta'ani, Tariq A Alalwan, Khurshid Alam, Rasmieh Mustafa Al-amer, Amani Alansari, Fahmi Y Al-Ashwal, Mohammed Albashtawy, Wafa A Aldhaleei, Abdelazeem M Algammal, Dari Alhuwail, Abid Ali, Mohammed Usman Ali, Waad Ali, Samah W Al-Jabi, Ahmad Alkhatib, Mahmoud A Alomari, Saleh A Alqahtani, Ahmad Alrawashdeh, Intima Alrimawi, Sahel Majed Alrousan, Najim Z Alshahrani, Zaid Altaany, Awais Altaf, Nelson Alvis-Guzman, Mohammad Al-Wardat, Yaser Mohammed Al-Worafi, Hany Aly, Safwat Aly, Hosam Alzahrani, Abdallah Alzoubi, Karem H Alzoubi, Sohrab Amiri, Hubert Amu, Dickson A Amugsi, Ganiyu Adeniyi Amusa, Roshan A Ananda, Robert Ancuceanu, Sumbul Ansari, Catherine M Antony, Boluwatife Stephen Anuoluwa, Iyadunni Adesola Anuoluwa, Saeid Anvari, Saleha Anwar, Anayochukwu Edward Anyasodor, Geminn Louis Carace Apostol, Juan Pablo Arab, Jalal Arabloo, Hidayat Arifin, Mesay Arkew, Benedetta Armocida, Johan Ärnlöv, Mahwish Arooj, Kurnia Dwi Artanti, Ashokan Arumugam, Seyyed Shamsadin Athari, Prince Atorkey, Alok Atreya, Adedapo Wasiu Awotidebe, Amirali Azimi, Sadat Abdulla Aziz, Ahmed Y. Azzam, Domenico Azzolino, Peter S Azzopardi, Mina Babashahi, Giridhara Rathnaiah Babu, Ashish D Badiye, Yogesh Bahurupi, Ruhai Bai, Atif Amin Baig, Shankar M Bakkannavar, Senthilkumar Balakrishnan, Ovidiu Constantin Baltatu, Kiran Bam, Maciej Banach, Mainak Bardhan, Hiba Jawdat Barqawi, Simon Barquera, Lingkan Barua, Mohammad-Mahdi Bastan, Reza Bayat, Mulat Tirfie Bayih, Tahmina Begum, Abdulrahman Babatunde Bello, Umar Muhammad Bello, Luis Belo, Isabela M Bensenor, Maria Bergami, Kidanemaryam Berhe, Ajeet Singh Bhadoria, Akshaya Srikanth Bhagavathula, Neeraj Bhala, Jaideep Singh Bhalla, Ravi Bharadwaj, Sonu Bhaskar, Ajay Nagesh Bhat, Priyadarshini Bhattacharjee, Shuvarthi Bhattacharjee, Gurjit Kaur Bhatti, Jasvinder Singh Bhatti, Andras Bikov, Catherine Bisignano, Bijit Biswas, Bruno Bizzozero Peroni, Espen Bjertness, Tone Bjørge, Srinivasa Rao Bolla, Hamed Borhany, Samuel Adolf Bosoka, Souad Bouaoud, Edward J Boyko, Dejana Braithwaite, Javier Brazo-Sayavera, Hermann Brenner, Gabrielle Britton, Dana Bryazka, Raffaele Bugiardini, Felix Busch, Yasser Bustanji, Daniela Calina, Luciana Aparecida Campos, Ismael Campos-Nonato, Yin Cao, Angelo Capodici, Andre F Carvalho, Márcia Carvalho, Alberico L Catapano, Monica Cattafesta, Maria Sofia Cattaruzza, Luca Cegolon, Francieli Cembranel, Edina Cenko, Ester Cerin, Joshua Chadwick, Vijay Kumar Chattu, Anis Ahmad Chaudhary, Akhilanand Chaurasia, An-Tian Chen, Guangjin Chen, Haowei Chen, Nicholas WS Chew, Ritesh Chimoriya, Patrick R Ching, Bryan Chong, Shivani Chopra, Dinh-Toi Chu, Sunghyun Chung, Karly I Cini, Rebecca M Cogen, Daniel Collado-Mateo, Alyssa Columbus, Nathalie Conrad, Michael H Criqui, Natalia Cruz-Martins, Steven Cummins, Alanna Gomes da Silva, Xiaochen Dai, Mayank Dalakoti, Emanuele D'Amico, Lucio D'Anna, Pojsakorn Danpanichkul, Samuel Demissie Darcho, Ivan Delgado-Enciso, Edgar Denova-Gutiérrez, Meseret Derbew Molla, Ismail Dergaa, Devananda Devegowda, Syed Masudur Rahman Dewan, Arkadeep Dhali, Samath Dhamminda Dharmaratne, Meghnath Dhimal, Bibha Dhungel, Daniel Diaz, Monica Dinu, Camila Bruneli do Prado, Milad Dodangeh, Sushil Dohare, Klara Georgieva Dokova, Neda Dolatkhah, Mario D'Oria, Ojas Prakashbhai Doshi, Rajkumar Prakashbhai Doshi, Robert Kokou

Dowou, Mi Du, Samuel C Dumith, Dorothea Dumuid, Bruce B Duncan, Arkadiusz Marian Dziedzic, Behrad Eftekhari, Ashkan Eighaei Sedeh, Michael Ekholuenetale, Rabie Adel El Arab, Ibrahim Farahat El Bayoumy, Said El-Ashker, Iffat Elbarazi, Islam Y Elgendy, Muhammed Elhadi, Ashraf A El-Metwally, Mohamed A Elmonem, Mohamed Hassan Elnaem, Randa Elsheikh, Chadi Eltaha, Theophilus I Emeto, Maysa Eslami, Ugochukwu Anthony Eze, Heidar Fadavian, Adeniyi Francis Fagbamigbe, Seyed Nooreddin Faraji, Carla Sofia e Sá Farinha, MoezAlIslam Ezzat Mahmoud Faris, Samuel Aanuoluwapo Fasusi, João C Fernandes, Rodrigo Fernandez-Jimenez, Nuno Ferreira, Florian Fischer, David Flood, Nataliya A Foigt, Morenike Oluwatoyin Folayan, Roham Foroumadi, Matteo Foschi, Maryam Fotouhi, Kate Louise Francis, Aleš Gába, Muktar A Gadanya, Emmanuela Gakidou, Silvano Gallus, Balasankar Ganesan, Shivaprakash Gangachannaiah, Miglas Welay Gebregergis, Lemma Getacher, Fataneh Ghadirian, Amir Ghaffari Jolfayi, Seyyed-Hadi Ghamari, Ramy Mohamed Ghazy, Artyom Urievich Gil, Tiffany K Gill, Elena V Gnedovskaya, Mahaveer Golechha, Davide Golinelli, Michal Grivna, Ashna Grover, Shi-Yang Guan, Giovanni Guarducci, Mohammed Ibrahim Mohialdeen Gubari, Avirup Guha, Damitha Asanga Gunawardane, Rahul Gupta, Rajeev Gupta, Sapna Gupta, Vivek Kumar Gupta, Roberth Steven Gutiérrez-Murillo, Najah R Hadi, Zahra Hadian, Nadia M Hamdy, Sajid Hameed, Mohammad Hamiduzzaman, Nasrin Hanifi, Graeme J Hankey, Allie Haq, Netanja I Harlianto, Josep Maria Haro, Faizul Hasan, Mohammad Hashem Hashempur, Md Saquib Hasnain, Amr Hassan, Nageeb Hassan, Rasmus J Havmoeller, Simon I Hay, Wen-Qiang He, Jeffrey J Hebert, Golnaz Heidari, Mehdi Hemmati, Yuta Hiraike, Nguyen Quoc Hoan, Mai Hoang, Ramesh Holla, Ashley Mark Hopkins, Sorin Hostiuc, Zin Wai Htay, Chengxi Hu, Junjie Huang, Mohamed Ibrahim Husseiny, Hong-Han Huynh, Ivo Iavicoli, Anel Ibrayeva, Olayinka Stephen Ilesanmi, Irena M Ilic, Milena D Ilic, Arit Inok, Lalu Muhammad Irham, Md. Rabiul Islam, Rakibul M Islam, Sheikh Mohammed Shariful Islam, Nahlah Elkudssiah Ismail, Hiroyasu Iso, Gaetano Isola, Mosimah Charles Ituka, Chinwe Juliana Iwu-Jaja, Assefa N Iyasu, Louis Jacob, Shabbar Jaffar, Haitham Jahrami, Akhil Jain, Mihajlo Jakovljevic, Mohamed Lamrana Jalloh, Syed Sarmad Javaid, Sathish Kumar Jayapal, Shubha Jayaram, Rime Jebai, Felix K Jebasingh, Alelign Tasew Jema, Jost B Jonas, Jobinse Jose, Nitin Joseph, Charity Ehimwenma Joshua, Jacek Jerzy Jozwiak, Mikk Jürisson, Ali Kabir, Ashish Kumar Kakkar, Sanjay Kalra, Sivesh Kathir Kamarajah, Saddam Fuad Kanaan, Samuel Berchi Kankam, Kehinde Kazeem Kanmodi, Neeti Kapoor, Paschalis Karakasis, Reema A Karasneh, Yeganeh Karimi, Joonas H Kauppila, Gbenga A Kayode, Dimitrios Kehagias, Jessica A Kerr, Ariz Keshwani, Emmanuelle Kesse-Guyot, Himanshu Khajuria, Pantea Khalili, Ajmal Khan, Maseer Khan, Moien AB Khan, Shaghayegh Khanmohammadi, Khaled Khatab, Moawiah Mohammad Khatatbeh, Feriha Fatima Khidri, Atulya Aman Khosla, Sepehr Khosravi, Mahmood Khosrowjerdi, Jagdish Khubchandani, Jinho Kim, Kwanghyun Kim, Min Seo Kim, Adnan Kisa, Ladli Kishore, Mika Kivimäki, Michail Kokkorakis, Oleksii Korzh, Sindhura Lakshmi Koulmane Laxminarayana, Kewal Krishan, Chong-Han Kua, Barthelemy Kuate Defo, Mukhtar Kulimbet, Vishnutheertha Kulkarni, Ashish Kumar, Setor K Kunutsor, Om P Kurmi, Maria Dyah Kurniasari, Dian Kusuma, Carlo La Vecchia, Ben Lacey, Chandrakant Lahariya, Daphne Teck Ching Lai, Hanpeng Lai, Iván Landires, Kamaluddin Latief, Nhi Huu Hanh Le, Paul H Lee, Sang-woong Lee, Wei-Chen Lee, Yongze Li, Queran Lin, Daniel Lindholm, Simin Liu, Erand Llanaj, Justin Lo, José Francisco López-Gil, Stefan Lorkowski, Giancarlo Lucchetti, Alessandra Lugo, Angelina M Lutambi, Ellina Lytvyak, Zheng Feei Ma, Monika Machoy, Javier A Magaña Gómez, Nastaran Maghbouli, Mehrdad Mahalleh, Elham Mahmoudi, Rituparna Maiti, Kashish Malhotra, Ahmad Azam Malik, Iram Malik, Deborah Carvalho Malta, Abdullah A Mamun, Marjan Mansourian, Emmanuel Manu, Hamid Reza Marateb, Mirko Marino, Abdoljalal Marjani, Ramon Martinez-Piedra, Miquel Martorell, Sammer Marzouk, Stefano Masi, Yasith Mathangasinghe, Fernanda Penido Matozinhos, Thushara Matthias, Steven M McPhail, Enkeleint A Mechili, Riffat Mehboob, Asim

Mehmood, Man Mohan Mehndiratta, Hadush Negash Meles, Walter Mendoza, Ritesh G Menezes, Emiru Ayalew Mengistie, Sultan Ayoub Meo, Tomislav Mestrovic, Chamila Dinushi Kukulege Mettananda, Sachith Mettananda, Ana Carolina Micheletti Gomide Nogueira de Sá, Ted R Miller, Awoke Misganaw, Madhukar Mittal, Mona Gamal Mohamed, Nouh Saad Mohamed, Taj Mohammad, Sakineh Mohammad-Alizadeh-Charandabi, Mustapha Mohammed, Shafiu Mohammed, Ali H Mokdad, Stefania Mondello, Mohammad Ali Moni, Maryam Moradi, Shane Douglas Morrison, Francesk Mulita, Erin C Mullany, Yanjinlkham Munkhsaikhan, Efren Murillo-Zamora, Christopher J L Murray, Sani Musa, Ghulam Mustafa, Sathish Muthu, Woojae Myung, Mohsen Naghavi, Gopal Nambi, Jobert Richie Nansseu, Gustavo G Nascimento, Mahmoud Nassar, Zuhair S Natto, Javaid Nauman, Samidi Nirasha Kumari Navaratna, Biswa Prakash Nayak, Md Fahad Shahariar Nayon, Masoud Negahdary, Ionut Negoi, Ruxandra Irina Negoi, Soroush Nematollahi, Samata Nepal, Marie Ng, Josephine W Ngunjiri, Cuong Tat Nguyen, Dang Nguyen, Duc Hoang Nguyen, Trang Nguyen, Robina Khan Niazi, Luciano Nieddu, Mahdieh Niknam, Jan Rene Nkeck, Mamoona Noreen, Masoud Noroozi, Nawsherwan, Jean Jacques Noubiap, Mehran Nouri, Chisom Adaobi Nri-Ezedi, Fred Nugen, Dieta Nurrika, Ogochukwu Janet Nzoputam, Bogdan Oancea, Erin M O'Connell, James Odhiambo Oguta, In-Hwan Oh, Osaretin Christabel Okonji, Andrew T Olagunju, Oladotun Victor Olalusi, Timothy Olusegun Olanrewaju, Arão Belitardo Oliveira, Gláucia Maria Moraes Oliveira, Hany A Omar, Marcel Opitz, Michal Ordak, Alberto Ortiz, Augustus Osborne, Alaa A M Osman, Wael M S Osman, Uchechukwu Levi Osuagwu, Adrian Otoiu, Amel Ouyahia, Mayowa O Owolabi, Irene Amoakoh Owusu, Kolapo Oyebola, Mahesh Padukudru P A, Alicia Padron-Monedero, Jagadish Rao Padubidri, Sujogya Kumar Panda, Songhomitra Panda-Jonas, Seithikurippu R Pandi-Perumal, Shahina Pardhan, Romil R Parikh, Roberto Passera, Hemal M Patel, Dimitrios Patoulias, George C Patton, Susan Paudel, Hamidreza Pazoki Toroudi, Umberto Pensato, Gavin Pereira, Marcos Pereira, Arokiasamy Perianayagam, Norberto Perico, Simone Perna, Ionela-Roxana Petcu, Fanny Emily Petermann-Rocha, Hoang Nhat Pham, Djordje S Popovic, Jalandhar Pradhan, Pranil Man Singh Pradhan, Manya Prasad, Akila Prashant, Elton Junio Sady Prates, Jagadeesh Puvvula, Jia-Yong Qiu, Venkatraman Radhakrishnan, Maja R Radojčić, Mohammad Hifz Ur Rahman, Saeed Rahmani, Ivano Raimondo, Jeffrey Pradeep Raj, Prashant Rajput, Mahmoud Mohammed Ramadan, Chitra Ramasamy, Shakthi Kumaran Ramasamy, Kritika Rana, Mithun Rao, Sowmya J Rao, Sina Rashedi, Mohammad-Mahdi Rashidi, Ashkan Rasouli-Saravani, Devarajan Rathish, Ilari Rautalin, Salman Rawaf, Elrashdy M. Moustafa Mohamed Redwan, Sanika Rege, Ana Reis-Mendes, Giuseppe Remuzzi, Hossein Rezazadeh, João Rocha Rocha-Gomes, Mónica Rodrigues, Thales Philipe Rodrigues da Silva, Jefferson Antonio Buendia Rodriguez, Leonardo Roever, Peter Rohloff, Debby Syahru Romadlon, Gholamreza Roshandel, Nitai Roy, Godfrey M Rwegerera, Aly M A Saad, Maha Mohamed Saber-Ayad, Cameron John Sabet, Kabir P Sadarangani, Basema Ahmad Saddik, Masoumeh Sadeghi, Umar Saeed, Sahar Saeedi Moghaddam, Dominic Sagoe, Fatemeh Saheb Sharif-Askari, Amirhossein Sahebkar, Soumya Swaroop Sahoo, Luciane B Salaroli, Marwa Rashad Salem, Vijaya Paul Samuel, Abdallah M Samy, Milena M Santric-Milicevic, Ushasi Saraswati, Aswini Saravanan, Dianis Wulan Sari, Tanmay Sarkar, Mohammad Sarmadi, Gargi Sachin Sarode, Sachin C Sarode, Michele Sassano, Susan M Sawyer, Ganesh Kumar Saya, Maria Inês Schmidt, Art Schuermans, Aletta Elisabeth Schutte, Siddharthan Selvaraj, Ashenafi Kibret Sendekie, Yigit Can Senol, Sadaf G Sepanlou, Yashendra Sethi, Allen Seylani, Mahan Shafie, Samiah Shahid, Moyad Jamal Shahwan, Muhammad Aaqib Shamim, Mehran Shams-Beyranvand, Anas Shamsi, Alfiya Shamsutdinova, Dan Shan, Mohd Shanawaz, Mohammed Shannawaz, Medha Sharath, Amin Sharifan, Anupam Sharma, Manoj Sharma, Ujjawal Sharma, Vishal Sharma, Ali Sheikhy, Rekha Raghuveer Shenoy, Pavanchand H Shetty, Min-Jeong Shin, Aminu Shittu, Seyed Afshin Shorofi, Kerem Shuval, Emmanuel Edwar Siddig, Ahmed

Kamal Siddiqi, Mithun Sikdar, Diego Augusto Santos Silva, Luís Manuel Lopes Rodrigues Silva, Harmanjit Singh, Jasvinder A Singh, Kalpana Singh, Surjit Singh, Valentin Yurievich Skryabin, Anna Aleksandrovna Skryabina, Amanda E Smith, Sameh S M Soliman, Soroush Soraneh, Michael Spartalis, Bahadar S Srichawla, Panagiotis Stachteas, Antonina V Starodubova, Pete Stubbs, Vetriselvan Subramaniyan, Muritala Odidi Suleiman Odidi, Aleksander Sulkowski, Anusha Sultan Meo, Chandan Kumar Swain, Lukasz Szarpak, Sree Sudha T Y, Fatemeh Sadat Tabatabaei, Shima Tabatabai, Celine Tabche, Mohammad Tabish, Stella Talic, Mircea Tampa, Jacques Lukenze Tamuzi, Ker-Kan Tan, Manoj Tanwar, Saba Tariq, Nathan Y Tat, Seyed Mohammad Tavangar, Mohamad-Hani Temsah, Reem Mohamad Hani Temsah, Dufera Rikitu Terefa, Jay Tewari, Tenaw Yimer Tiruye, Sojit Tomo, Marcello Tonelli, Mathilde Touvier, Marcos Roberto Tovani-Palone, Khaled Trabelsi, Mai Thi Ngoc Tran, Thang Huu Tran, Nguyen Tran Minh Duc, Domenico Trico, Thien Tan Tri Tai Truyen, Aristidis Tsatsakis, Gary Tse, Sok Cin Tye, Stefanos Tyrovolas, Aniefiok John Udoakang, Shahid Ullah, Muhammad Umair, Lawan Umar, Umar Muhammad Umar, Brigid Unim, Dinesh Upadhya, Era Upadhyay, Jibrin Sammani Usman, Damla Ustunsoz, Asokan Govindaraj Vaithinathan, Jef Van den Eynde, Joe Varghese, Tommi Juhani Vasankari, Balachandar Vellingiri, Narayanaswamy Venketasubramanian, Anjul Verma, Madhur Verma, Georgios-Ioannis Verras, Simone Vidale, Victor E Villalobos-Daniel, Manish Vinayak, Vasily Vlassov, Rade Vukovic, Mugi Wahidin, Mohammad Wahiduzzaman, Cong Wang, Shu Wang, Xingxin Wang, Yanzhong Wang, Mary Njeri Wanjau, Ahmed Bilal Waqar, Kosala Gayan Weerakoon, Fei-Long Wei, Dakshitha Praneeth Wickramasinghe, Peter Willeit, Marcin W Wojewodzic, Tewodros Eshete Wonde, Qing Xia, Kazumasa Yamagishi, Haiqiang Yao, Amir Yarahmadi, Dong Keon Yon, Naohiro Yonemoto, Deniz Yuce, Sojib Bin Zaman, Iman Zare, Michael Zastrozhin, Mohammed G M Zeariya, Casper J P Zhang, Xiaoyi Zhang, Zhiqiang Zhang, David X Zheng, Anthony Zhong, Claire Chenwen Zhong, Magdalena Zielińska, Ghazal Zoghi, Zhiyong Zou, and Sa'ed H Zyoud.

Managing the estimation or publication process

Catherine M Antony, Xiaochen Dai, Emmanuela Gakidou, Simon I Hay, Nicholas J Kassebaum, Jessica A Kerr, Paulina A Lindstedt, Justin Lo, Ali H Mokdad, Christopher J L Murray, Mohsen Naghavi, Erin M O'Connell, George C Patton, Susan M Sawyer, Amanda E Smith, and Stein Emil Vollset.
